# A Bambusuril Receptor Binds Charge Diffuse Anions in Water at Picomolar Concentrations

**DOI:** 10.1002/anie.202510912

**Published:** 2025-07-29

**Authors:** Surbhi Grewal, Petr Slávik, Vladimír Šindelář

**Affiliations:** ^1^ Department of Chemistry, Faculty of Science Masaryk University Kamenice 5, 625 00 Brno Czech Republic; ^2^ RECETOX, Faculty of Science Masaryk University Kamenice 5, 625 00 Brno Czech Republic

**Keywords:** Bambusurils, Binding in water, Chaotropic effect, Macrocycles, Supramolecular chemistry

## Abstract

Anionic pollutants are widespread in the environment and pose risks to human health. Due to the typically low concentrations of these species, the development of efficient receptors for their detection and removal from aqueous solutions is highly desirable. Here, we report the design and synthesis of a bambusuril receptor with markedly enhanced anion‐binding affinity in water, achieved by introducing trifluoromethyl electron‐withdrawing groups. The resulting fluorinated receptor is, to date, the most potent known receptor for several charge diffuse anions in aqueous media. The most stable 1:1 host–guest complex in water was observed with iodide, exhibiting a relative dissociation constant of 59 pM. This complex is over three orders of magnitude more stable than any previously reported complex between an artificial receptor and inorganic anion in water.

## Graphical abstract

Synthesis of a bambusuril macrocycle with unprecedented binding affinity for several charge diffuse anions, including iodide, perchlorate, nitrate, and perrhenate, in neutral phosphate buffer is reported. The macrocycle exhibits selectivity for charge diffuse over charge dense anions exceeding seven orders of magnitude.

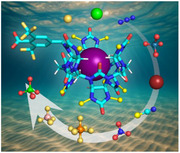

The selective recognition of anions in aqueous environments remains one of the major challenges in contemporary supramolecular chemistry.^[^ ^1^, ^2^ ^]^ The vast majority of reported anion receptors function effectively only in organic solvents, as such environments offer a more favorable setting for noncovalent interactions due to their lower polarity.^[^ ^3^ ^]^ In contrast, anion binding in water is associated with a high desolvation penalty, which must be compensated by strong and directional host–guest interactions. Additionally, receptor design is often complicated by limited solubility in pure water.

These issues were originally addressed by designing positively charged host molecules that utilize electrostatic interactions for anion binding while also ensuring good water solubility.^[^ ^4^, ^5^, ^6^, ^7^ ^]^ More recent examples of anion receptors take advantage of other noncovalent interactions, including hydrogen bonding motifs such as NH, C(sp^2^)H, and C(sp^3^)H, as well as halogen and chalcogen bonding. Highly potent anion receptors functioning in water, including metal‐organic cages,^[^ ^8^, ^9^ ^]^ organic cages,^[^ ^10^, ^11^, ^12^, ^13^, ^14^ ^]^ and macrocyclic compounds,^[^ ^15^, ^16^, ^17^, ^18^, ^19^ ^]^ have been developed by incorporating several of these binding motifs into their structures. Furthermore, binding affinity can be enhanced by shielding the anion binding site from water molecules.^[^ ^15^, ^18^, ^20^, ^21^ ^]^

A limited number of deep cavitands, such as bambus[6]urils and biotin[6]urils, are known to enhance anion binding in water by expelling high‐energy water molecules from their cavities upon anion binding.^[^ ^15^, ^18^, ^22^ ^]^ These macrocycles are especially well‐suited for binding large anions with a low volume‐to‐charge ratio, such as I⁻, ClO₄⁻, and TcO₄⁻, which are also classified as water‐structure breakers known as charge diffuse anions or chaotropes.^[^ ^23^ ^]^ These toxic anions are environmental contaminants that pose substantial risks to human health and ecosystems.^[^ ^24^, ^25^, ^26^ ^]^ For instance, ClO₄⁻ is released into water systems from propellants and solid rocket fuels, while radioisotopes such as ^129^I⁻, ^131^I⁻, and ^99^TcO₄⁻ are associated with nuclear waste. Consequently, the sensing and removal of these anions are of significant interest.

Previously, we reported that bambus[6]urils (Figure 1) can bind charge diffuse anions at submicromolar concentrations in both pure and buffered water.^[^ ^15^, ^16^, ^17^, ^27^ ^]^ However, these affinities are still insufficient for effective detection and removal of such contaminants from their highly diluted environmental sources. In this work, we applied our recently developed strategy^[^ ^28^ ^]^ to significantly improve the anion‐binding affinity of bambusurils in water. We report the synthesis of novel bambusuril derivatives that demonstrate binding constants in the picomolar range for inorganic anions in water, even in the presence of competitive salts.

We started our investigation with the molecular design of a new macrocycle. We decided to utilize the structure of our previously reported bambusuril **BU1** (Figure 1) which bears 12 benzyl groups with attached carboxylic groups.^[^ ^15^ ^]^ It was shown that this macrocycle is soluble in neutral buffered water at millimolar concentrations. Previously, we also showed that installing electron‐withdrawing groups on the benzyl substituent enhances affinity of bambusurils for inorganic anions in acetonitrile.^[^ ^28^ ^]^ We hypothesized that introducing two electron‐withdrawing trifluoromethyl groups on each of the carboxylbenzyl substituents of **BU1** would increase in the binding affinity of the macrocycle, while the presence of the carboxyl groups would maintain the solubility of the macrocycle in water. This hypothesis was supported by results from molecular modeling (Figure 1). The presence of CF₃ groups in **BU2** caused a substantial shift of electron density from the methine proton region inside the macrocyclic cavity towards the benzyl substituents on the portals, as compared to **BU1**. Thus, **BU2** was expected to bind anions inside its cavity more strongly than **BU1**.

**Figure 1:**
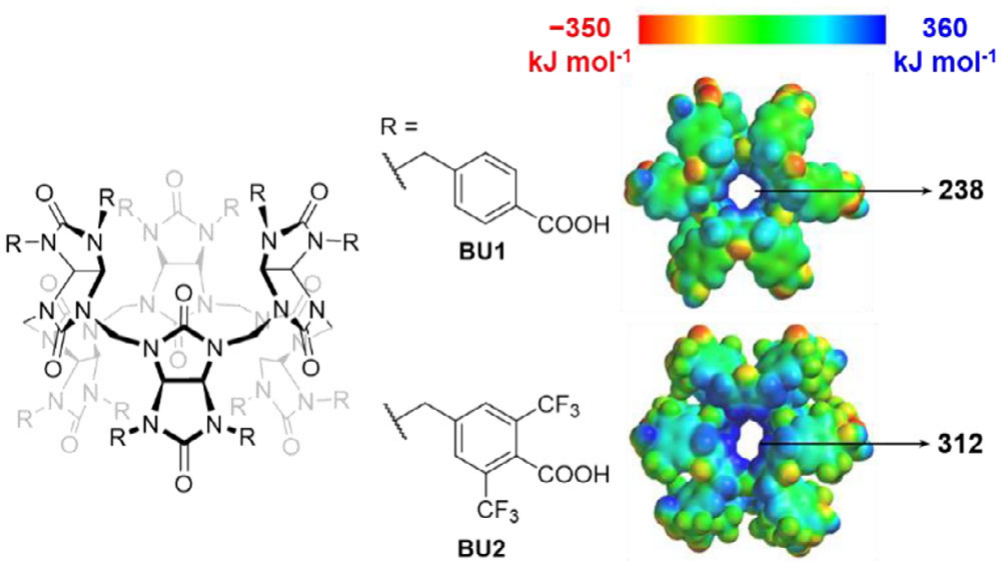
Structure and electrostatic potential map of **BU1** and **BU2**.

The synthesis of bambusuril **BU2** started with the preparation of the benzyl derivative **1** and the glycoluril derivatives **2** (Scheme 1). Compound **1** was synthesized starting from 1,3‐bis(trifluoromethyl)benzene by a multistep synthesis (see Supporting Information). We originally prepared the methyl ester instead of the *tert*‐butyl ester of this compound. However, only partial conversion of the methyl ester to the carboxylic acid was observed, while the conversion of the *tert*‐butyl ester **4** to **5** reaction proceeded without complication. Glycoluril **2** was prepared according to a previously published protocol.^[^ ^29^ ^]^

**Scheme 1:**
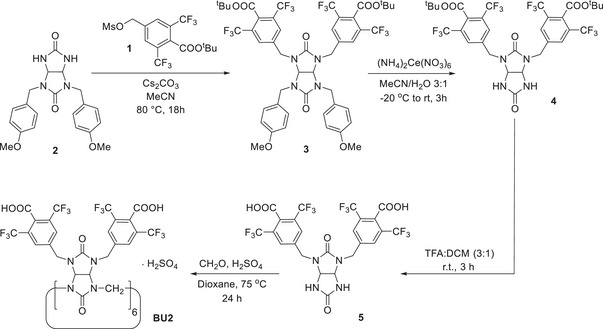
Synthesis of HSO₄⁻⊂**BU2**.

The alkylation of glycoluril **2** by **1** was performed in the presence of Cs₂CO₃ in CH₃CN at 70 °C, yielding the tetrasubstituted glycoluril **3** (Scheme 1).^[^ ^30^ ^]^ After deprotection of the 4‐methoxybenzyl groups, hydrolysis of the *tert*‐butyl ester using trifluoroacetic acid (TFA) in DCM was carried out to obtain glycoluril **5**. Alternatively, glycoluril **5** could also be prepared by the traditional approach from the corresponding bis(alkyl)urea and 4,5‐dihydroxyimidazolidin‐2‐one.^[^ ^31^ ^]^ However, this approach led to the formation of side products such as hydantoins, which reduced the yield of the desired glycoluril. The final step was the macrocyclization reaction of glycoluril **5** with paraformaldehyde. The reaction was performed in 1,4‐dioxane in the presence of sulfuric acid, acting both as an acid catalyst and as a source of HSO₄⁻ anions to template the six‐membered macrocycle. As a result, **BU2** was obtained as its complex with HSO₄⁻ in 70% yield. Attempts to remove the bound HSO_4_^−^ anion prior to binding studies were unsuccessful and resulted in significant material loss. Therefore, we decided to use **BU2** as its complex with HSO₄⁻ for subsequent supramolecular studies.

First, we followed the binding of **BU2** and anions using ¹H NMR spectroscopy. The measurements were performed in D₂O containing 30 mM K₂DPO₄ at a pD of 7.1, which ensured good solubility for the macrocycle. When 0.5 equivalents of tetramethylammonium (TMA) bromide were added to the HSO₄⁻⊂**BU2** solution, new signals corresponding to the complex of **BU2** with Br⁻ were clearly observed alongside those of the original HSO₄⁻ complex (Figure 2). After the addition of 1.0 equivalent of NaBr, only signals corresponding to the new complex were detected in the NMR spectra. This observation is consistent with the formation of a host–guest complex with 1:1 stoichiometry. Moreover, it indicated that the complexation is slow on the NMR time scale. We observed a similar binding mode and slow exchange on the NMR time scale for other anions, including I⁻, ClO₄⁻, and NO₃⁻ (Figures S14–S17). Only in the case of Cl⁻ was the binding fast on the NMR time scale, likely due to its weaker interaction with **BU2** compared to the other tested anions (Figure S18).

**Figure 2:**
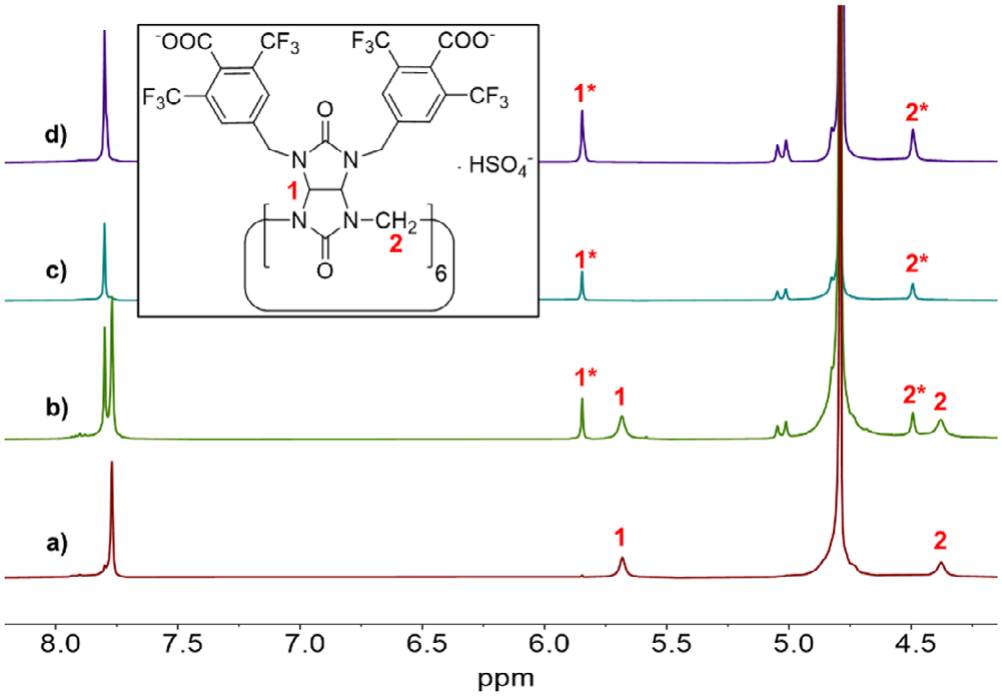
^1^H NMR (D_2_O, 30 mM K_2_DPO_4_, 500 MHz) spectra of the HSO_4_⁻⊂**BU2** complex (1 mM) a) in the absence and in the presence of b) 0.5 equiv, c) 1 equiv, and d) 1.2 equiv of tetramethylammonium (TMA) bromide. *Signals of the Br⁻⊂**BU2** complex.

Isothermal titration calorimetry (ITC) was used to quantify the binding affinity of the **BU2** complexes with anions, as well as the thermodynamic parameters of the host–guest interactions. The measurements were carried out in a phosphate buffer (30 mM K₂HPO₄, pH 7.1) at 25 °C, thus, the obtained values of the association constants for the host–guest complexes are apparent. For detailed information about the ITC measurements see the Supporting Information. ITC measurements confirmed that all anions form complexes with **BU2** of 1:1 stoichiometry. **BU2** forms the weakest complexes with fluoride and chloride, and the most stable complexes with I⁻, ClO₄⁻, and BF₄⁻ (Table 1). Thus, the stability of the complexes correlates with the solvation energy of the anions, as shown in Figure S20. Small anions with a low volume‐to‐charge ratio are more strongly solvated, and the large desolvation penalty is responsible for their lower binding affinity to the macrocycle compared to larger anions.

**Table 1:** Apparent association constants (*K*_a_), and thermodynamical parameters (Δ*H*, *T*Δ*S*, Δ*G*) of the sodium anion⊂**BU2** complexes determined by ITC in 30 mM aq. K_2_HPO_4_ (pH 7.1) at 298.15 K

Anion	*K*_a_ (M^−1^)	Δ*H* (kJ mol^−1^)	*T*Δ*S* (kJ mol^−1^)	Δ*G* (kJ mol^−1^)
F^−^	(4.1 ± 0.6) × 10^2^	−14.3	0.75	−15.0
Cl^−^	(4.5 ± 0.1) × 10^4^	−45.0	−18.5	−26.6
Br^−^	(3.1 ± 0.1) × 10^7^	−62.8	−20.1	−42.8
I^−^	(1.7 ± 0.2) × 10^10^	−87.0	−28.5	−58.5
N_3_^−^	(8.4 ± 0.4) × 10^5^	−70.1	−36.2	−33.9
ReO_4_^−^	(1.3 ± 0.1) × 10^7^	−73.8	−33.1	−40.7
SCN^−^	(3.3 ± 0.1) × 10^7^	−67.0	−24.0	−43.0
NO_3_^−^	(6.0 ± 0.4) × 10^7^	−63.4	−19.0	−44.5
PF_6_^−^	(8.3 ± 0.2) × 10^8^	−82.9	−31.9	−51.0
BF_4_^−^	(2.3 ± 0.1) × 10^9^	−83.4	−29.8	−53.6
ClO_4_^−^	(9.9 ± 0.4) × 10^9^	−86.6	−29.5	−57.2

Our investigation showed that anion binding with HSO₄⁻⊂**BU2** is driven by enthalpy, while it is compensated by an entropic factor (Figure 3). The observed enthalpy–entropy compensation can be explained by differences between the anions competing for the **BU2** cavity. During ITC experiments, a competing anion is added to a solution of the macrocycle, which is already occupied by HSO₄⁻ or potentially by HPO₄^2^⁻/H₂PO₄⁻ present in the buffered aqueous solution. Charge dense anions (also known as kosmotropes) such as SO₄^2^⁻ (generated upon the HSO₄⁻ release to water), HPO₄^2^⁻, and H₂PO₄⁻ are characterized by high hydration energies and highly ordered water molecules in their hydration shells. Replacing these charge dense anions with more charge‐diffuse anions, characterized by lower hydration energies and more disordered hydration shells, results in an enthalpically driven process that is compensated by an unfavorable entropy change. Therefore, differences in anion hydration are a major driving force for anion binding with bambusurils, along with multiple C–H···anion hydrogen bonding interactions between the methine hydrogen atoms and the anion inside the electron‐deficient cavity of the macrocycle.

**Figure 3:**
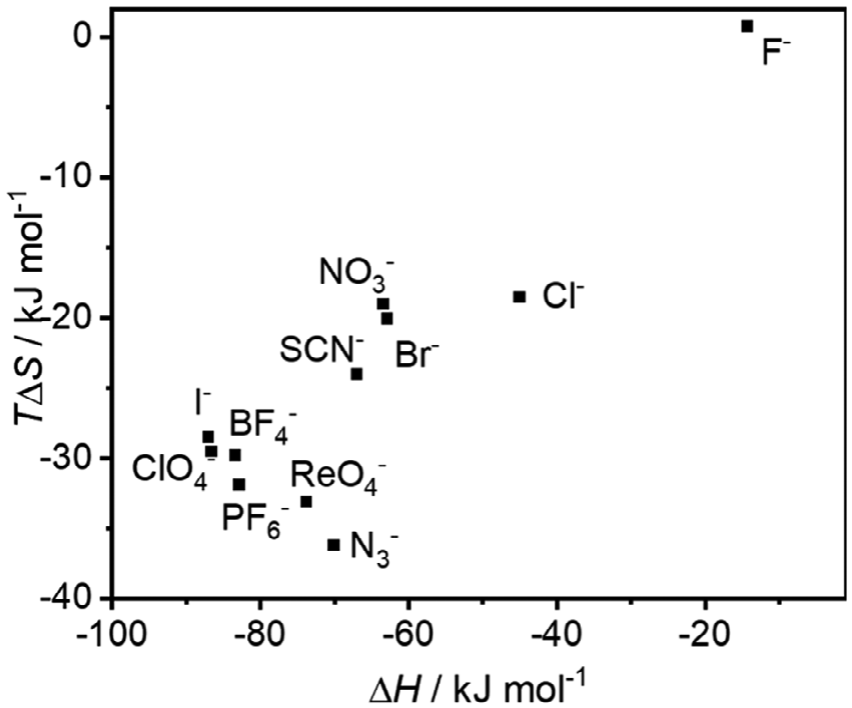
Enthalpy–entropy compensation plot of **BU2** complexed with anions.

The observed dependence of relative association constants and thermodynamic parameters on anion solvation and size is consistent with trends reported for other bambusuril derivatives when anion binding was performed in buffered water.^[^ ^15^, ^16^, ^17^, ^27^, ^32^, ^33^ ^]^ However, **BU2** is a significantly more potent receptor for inorganic anions in water than any previously reported bambusurils. Notably, the complexes between **BU2** and ReO₄⁻, Br⁻, SCN⁻, NO₃⁻, PF₆⁻, BF₄⁻, ClO₄⁻, and I⁻ showed surprisingly high stability, with association constants ranging from 77 nM to 59 pM. This makes **BU2** by far the most potent receptor for all these charge diffuse anions in water. As **BU2** differs from **BU1** by the presence of two CF₃ groups on each of the 12 benzyl substituents, we can directly observe the influence of these electron‐withdrawing groups on the binding properties of the macrocycle. Figure 4 shows that the difference in relative association constant values for the complexes of **BU1** and **BU2** is significantly less pronounced for complexes with more strongly solvated anions compared to those with charge diffuse anions such as Br⁻ and I⁻. These differences in binding result in pronounced selectivity of **BU2** for charge diffuse over charge dense anions. For example, **BU2** binds I⁻ over F⁻ and Cl⁻ with selectivities (deriving from *K*_a_ values of corresponding complexes) of 41 × 10⁶ and 0.4 × 10⁶, respectively.

**Figure 4:**
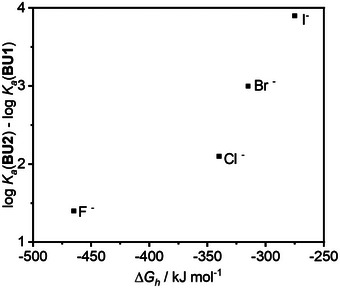
Dependence of the difference in log *K*_a_ values for **BU1** and **BU2** halide complexes on halide solvation.

It should be noted that the observed high binding affinity and selectivity of HSO₄⁻⊂**BU2** for anions were achieved in the presence of 30 mM K₂HPO₄. Furthermore, the macrocycle used for ITC measurements was in the form of its complex with HSO₄⁻. During titration with a solution of the selected salt, the HSO₄⁻ anion was expelled from the cavity of the macrocycle and converted into SO₄^2^⁻. Thus, all competing anions present in the solution are characterized by their high solvation energy and therefore do not effectively compete with the investigated charge diffuse anions. This was demonstrated using chloride, one of the most strongly solvated anions tested. The log *K*_a_ values for its complex with **BU2**, measured at K₂HPO₄ concentrations ranging from 10 mM to 1 M, varied by less than 10% (Figure S21).

In conclusion, this study demonstrated that the binding affinity of an anion receptor in water can be significantly enhanced by introducing electron‐withdrawing groups into its structure. We prepared **BU2**, containing 24 CF₃ groups, which enhanced the binding potency of the macrocycle by up to 1700 times compared to its non‐fluorinated analog, **BU1**. The enhancement in binding was especially pronounced for charge diffuse anions, resulting in unprecedentedly high stability of anion–host complexes in water, exemplified by a *K*_a_ value of 1.7 × 10¹⁰ M⁻¹ for the **BU2** complex with I⁻. These results highlight the importance of deep cavitands for effective binding in aqueous environments. Bambusurils represent rare examples of deep cavitands, where C(sp^3^)–H···anion interactions can be more effective because of the shielding of the binding site from bulk water. Furthermore, the choice of substituents at the macrocycle portals enables fine‐tuning of binding properties, while differences in anion hydration determine their preferential uptake by **BU2**. As a result, charge diffuse anions with low hydration energies and a tendency to disrupt the structure of bulk water are preferentially bound within the bambusuril cavity. Binding efficiency is further enhanced by complete anion desolvation and effective shielding from water molecules once the anion is enclosed within the cavity. Therefore, the demonstrated advantages of deep cavitands for efficient anion binding in water should be carefully considered in the design of future anion receptors.

## Conflict of Interests

The authors declare no conflict of interest.

## Supporting information



Supporting Information

## Data Availability

The data that support the findings of this study are available in the Supporting Information of this article.
