# Supplementary material for: A Bambusuril Receptor Binds Charge Diffuse Anions in Water at Picomolar Concentrations
Source: Angew Chem Int Ed Engl. 2025 Jul 29;64(37):e202510912. doi: 10.1002/anie.202510912 (PMC12416460; doi:10.1002/anie.202510912)
Supplement: Supplementary file 1 — Supporting Information [file ANIE-64-e202510912-s001.pdf]

Supporting Information

A Bambusuril Receptor Binds Charge Diffuse Anions in Water at Picomolar Concentrations

Surbhi Grewal,[a,b] Petr Slávik,[a,b] and Vladimír Šindelář*[a,b]

[a]*Department of Chemistry, Faculty of Science, Masaryk University, Kamenice 5, 625 00 Brno, Czech Republic*

[b]*RECETOX, Faculty of Science, Masaryk University, Kamenice 5, 625 00 Brno, Czech Republic, Email: sindelar@chemi.muni.cz*

TABLE OF CONTENTS

1. **Materials and Methods** **S2**
2. **Synthesis of Compounds** **S4**
3. **Isothermal Titration Calorimetry (ITC) Studies** **S10**
4. **NMR Titrations Showing Anion Binding of BU2** **S26**
5. **Dependence of Apparent Association Constants on Different Parameters** **S31**
6. **^{1}H, ^{19}F, and ^{13}C NMR Spectra of Synthesized Compounds** **S33**

1. Materials and Methods

All reagents and solvents were purchased from commercial suppliers and used without further purification. 4,5-Dihydroxyimidazolidin-2-one and 2,4-bis(4-methoxybenzyl)glycoluril **(2)** were prepared according to previously published procedures.[1,2] Mili-Q grade water was prepared by a Barnstead™ MicroPure™ Water Purification System. All reactions that require increased temperature were heated with a DrySyn heating block on an electromagnetic stirrer. Mixing of the reaction mixtures was done by a magnetic stirrer.

The NMR spectra were measured on the following spectrometer at 25 °C: Bruker Avance III 500 MHz (^{1}H: 500 MHz, ^{13}C: 126 MHz, ^{19}F: 471 MHz). Chemical shifts (δ) are reported in parts per million (ppm) and coupling constants (J) are given in Hertz (Hz). The ^{1}H and ^{13}C NMR spectra were referenced to the solvent residual signals; ^{19}F NMR spectra were unreferenced. The NMR spectra were processed using MestReNova 6.0.2.

HRMS spectra were recorded on an Agilent 6224 Accurate-Mass TOF mass spectrometer. Samples were ionized by electrospray ionization (ESI) or atmospheric-pressure chemical ionization (APCI). Matrix assisted laser desorption ionization with detection of time of flight (MALDI-TOF) mass spectra were measured on the MALDI-TOF MS UltrafleXtreme (Bruker Daltonics). Samples were ionized by Nd-YAG laser (355 nm) from 2,5-dihydroxybenzoic acid (DHB) matrix.

ITC analysis was performed on PEAQ - Auto-iTC200 from Malvern at 298.15 K in solution of K_2HPO_4 (10, 30, 300 mM, and 1M) in milli-Q water. The top graph in each figure (in the chapter 3.3. Isothermal Titration Calorimetry (ITC) Studies) displays the recorded heat responses during the titration. Each spike represents an injection of 1.3 µL of salt solution into the cell containing **BU2**. The number of injections for each measurement was 30. The lower graph illustrates the cumulative heat released as a function of the total concentration of the ligand. The solid line on the graph corresponds to the best-fit line obtained through a least-squares analysis of the data. Specific conditions for each measurement are provided in the legend of the corresponding figure.

<u>Direct titration</u>

Host–guest complexation between **BU2** and Cl^- was studied by injecting a NaCl solution into a cell containing the **BU2** solution. To analyse the integrated heat effects, a single-site model was employed, and MicroCal PEAQ-ITC analysis software was used. The association constant K_a and the standard binding enthalpy ΔH° were achieved using experimental data matched to a theoretical titration curve. The standard free energy ΔG° and standard entropy ΔS° were obtained through the equation: $\Delta G^\circ = \Delta H^\circ - T\Delta S^\circ = -RT \ln K_a$, where T is the absolute temperature and R is the molar gas constant (8.3145 J mol^{-1} K^{-1}).

<u>Competitive titration</u>

Complexes between **BU2** and other investigated anions but chloride, were determines by competition experiments. NaCl or NaBr were used as competitive salts. The anion of stronger affinity toward the macrocycle placed in syringe was injected into titration cell which contained the solution of **BU2** and the weaker binding anion. In the case of the $F^-\subset$**BU2** complex, a solution

containing both NaF and **BU2** was titrated with a NaCl solution. Complexes of **BU2** with iodide were evaluated by injecting a solution of NaI into a solution containing **BU2** and NaBr. Complexes of **BU2** with all remaining anions were evaluated by injecting a solution of the sodium salt of the respective anion into a solution containing **BU2** and NaCl. We employed the model supplied with the instrument that takes into consideration the change of concentration of all reagents. The use of two step determination of association constants by competition experiment increases the uncertainty of obtained absolute values, but it mitigates the influence of dilution of competing guest which has to be present in higher concentration in the measuring cell.

The electrostatic potential was calculated in Spartan'18 1.4.4 after the structures were optimized with the semi-empirical PM6 method in vacuum.

2. Synthesis of Compounds

2.1 Synthesis of 2,6-bis(trifluoromethyl)benzoic acid 2a

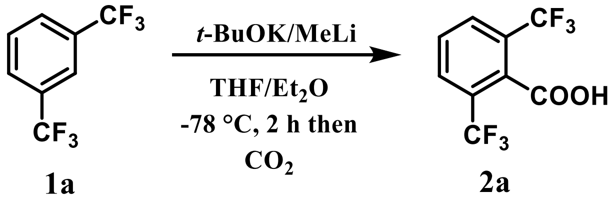

1a **2a**

Potassium *tert*-butoxide (3.1 g, 27.63 mmol, 1.12 eq) was transferred to an oven-dried three-neck 250-ml RBF which was flushed with argon. Then, THF (50 ml, anhydrous) was added, and the flask was cooled to -78 °C with dry ice bath. Methyllithium (1.6 M in diethyl ether, 20.5 ml, 28.80 mmol, 1.17 eq) was added to the flask dropwise over 15 minutes, giving a clear yellow solution. Further, 1,3-bis(trifluoromethyl)benzene **1a** (5.3 g, 24.64 mmol, 3.9 ml, 1.00 eq) was added dropwise over 15 minutes, the colour changed from yellow to deep purple with the first drop. The resulting purple solution was stirred at –78 °C for 3 h. To the reaction mixture, dry ice (1.3 g) was added in one portion (exothermic reaction was observed) and the solution changed to a black slurry which was swirled manually. The flask was then allowed to warm up to room temperature and stirred for another 1 hour. The reaction mixture was quenched with water (30 ml) and concentrated on rotary evaporator to remove THF. The mixture was washed with diethyl ether (5×30 ml). Further, the remaining aqueous part was acidified with HCl (35%) until pH 1 followed by extraction with DCM (4×30 ml). Combined organic layers were washed with brine (40 ml) followed by drying with anhydrous magnesium sulphate. The drying agent was filtered off and the filtrate was concentrated on ROTAVAP to give a yellow solid which was dried *in vacuo* (4.3 g, 68%).

^{1}H NMR (500 MHz, $CDCl_3$): δ = 9.27 (br, 1H, -COOH), 7.96 (d, J = 8 Hz, 2H; Ar-H), 7.75 (t, J = 8 Hz, 1H; Ar-H). **^{13}C{^{1}H} NMR** (125 MHz, $CDCl_3$): δ = 170.2, 130.7, 130.1 (q, J = 4.4 Hz), 129.5 (app. m), 129.3 (q, J = 32.7 Hz), 122.9 (q, J = 272.6 Hz). **^{19}F NMR** (471 MHz, $CDCl_3$): δ = −59.56. **HRMS** (APCI): m/z calcd for $C_9H_4F_6O_2$-H^-: 257.0043 $[M-H]^-$; found: 257.0045.

2.2 Synthesis of 4-bromo-2,6-bis(trifluoromethyl)benzoic acid 3a

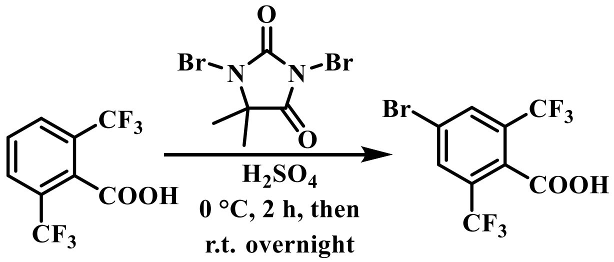

2a **3a**

Sulphuric acid (96%, 40 ml) was transferred to a 100-ml RBF containing 2,6-bis(trifluoromethyl)benzoic acid **2a** (10 g, 38.74 mmol, 1.01 eq). The mixture was cooled to 0 °C and DBDMH (11 g, 38.47 mmol, 1.00 eq) was added in one portion, creating a beige dense slurry. The cooling bath was removed after 2 hours and the slurry was stirred at room temperature overnight. The beige slurry was poured into cold water (600

ml), resulting in beige coloured precipitate. The mixture was stored in the fridge for 3 hours. The precipitate was filtered out, washed with water (50 ml) and dried *in vacuo* (10.5 g, 80%).

^{1}H NMR (500 MHz, $CDCl_3$): δ = 8.08 (s, 2H; Ar-H). **^{13}C{^{1}H} NMR** (125 MHz, $CDCl_3$): δ = 169.3, 133.3 (q, *J* = 4.6 Hz), 130.9 (q, *J* = 33.1 Hz), 128.4 (app. m), 124.9, 122 (q, *J* = 273.2 Hz). **^{19}F NMR** (471 MHz, $CDCl_3$): δ = −59.74. **HRMS** (ESI): m/z calcd for $C_9H_3BrF_6O_2$-H^-: 334.9148 $[M-H]^-$; found: 334.9148.

2.3 Synthesis of *tert*-butyl 4-bromo-2,6-bis(trifluoromethyl)benzoate 4a

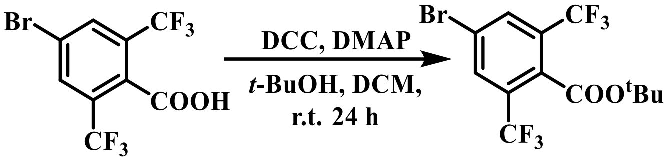

3a 4a

A three-neck 25-ml RBF was flushed with argon and charged with 4-bromo-2,6-bis(trifluoromethyl)benzoic acid **3a** (337 mg, 1.0 mmol, 1 eq), DCC (412 mg, 2.0 mmol, 2 eq) and DMAP (24 mg, 0.20 mmol, 0.2 eq) and DCM (2.0 ml) were added. Then, *tert*-butyl alcohol (741 mg, 10.0 mmol, 10 eq) was added. The mixture was stirred under argon at room temperature for 24 h. The resulting mixture was filtered to remove insoluble material. The filtrate was concentrated, and the obtained crude material was purified by flash column chromatography (gradient elution, cyclohexane/ethyl acetate: 0-50%) to yield a white solid (205 mg, 52%).

^{1}H NMR (500 MHz, $CDCl_3$): δ = 8.01 (s, 2H; Ar-H), 1.57 (s, 9H; -$C(CH_3)_3$). **^{13}C{^{1}H} NMR** (125 MHz, $CDCl_3$): δ = 163.0, 133.0 (q, *J* = 4.8 Hz), 131.4 (app. m), 130.5 (q, *J* = 33 Hz), 123.5, 122.5 (q, *J* = 273.4 Hz), 85.3, 27.7. **^{19}F NMR** (471 MHz, $CDCl_3$): δ = −59.43. **HRMS** (ESI): m/z calcd for $C_{13}H_{11}BrF_6O_2$-H^-: 390.9774 $[M-H]^-$; found: 390.9775.

2.4 Synthesis of *tert*-butyl 4-formyl-2,6-bis(trifluoromethyl)benzoate 5a

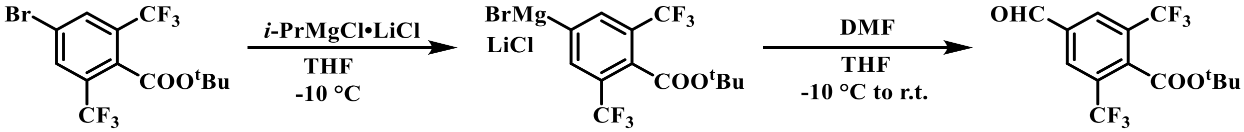

4a 5a

100-ml RBF containing the starting ester **4a** (1.5 g, 3.83 mmol, 1 eq) was flushed with argon. THF (20 ml, anhydrous) was added, giving a clear solution. The mixture was cooled to –10 °C with ice/acetone bath. Turbo Grignard (1.3 M in THF) was added to the reaction mixture dropwise (3.62 ml, 4.71 mmol, 1.23 eq) over 35 minutes, giving a brownish green solution. The resulting solution was stirred at –10 °C for 1.5 hour. Then, DMF (1.5 ml, anhydrous) was added to the reaction mixture dropwise over 10 minutes at –5 °C, the cooling bath was removed and the resulting brown solution was stirred at room temperature for 1 hour. Saturated solution of NH_4Cl was added to the reaction mixture. The yellow biphasic solution was stirred vigorously at room temperature for 1 hour. The THF was removed on rotary evaporator and the aqueous

solution was extracted with DCM (3×50 ml). Combined organic layers were washed with brine (50 ml) and dried over anhydrous magnesium sulphate. The drying agent was filtered off and the yellow filtrate was concentrated on rotary evaporator to give an orange oil. After that, the obtained crude product was purified using flash column chromatography (gradient elution, cyclohexane/ethyl acetate: 0-50%) to yield pale yellow solid (980 mg, 75%).

^{1}H NMR (500 MHz, $CDCl_3$): δ = 10.12 (s, 1H; -CHO), 8.38 (s, 2H; Ar-H), 1.59 (s, 9H; $-C(CH_3)_3$). **^{13}C{^{1}H} NMR** (125 MHz, $CDCl_3$): δ = 188.6, 162.6, 136.9, 136.6, 130.6 (q, *J* = 4.6 Hz), 130.3 (q, *J* = 33.1 Hz), 122.6 (q, *J* = 273.2 Hz), 85.9, 27.7. **^{19}F NMR** (471 MHz, $CDCl_3$): δ = −59.50. **HRMS** (APCI): m/z calcd for $C_{14}H_{12}F_6O_3$: 342.0696 $[M]^-$; found: 342.0691.

2.5 Synthesis of *tert*-butyl 4-(hydroxymethyl)-2,6-bis(trifluoromethyl)benzoate 6a

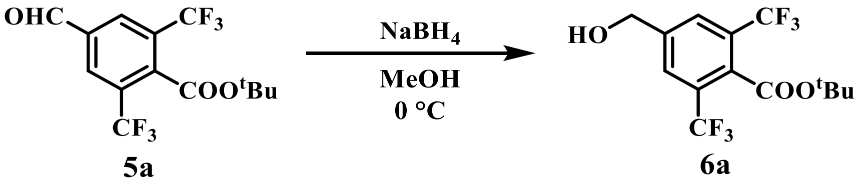

The starting aldehyde **5a** (1.05 g, 3.1 mmol, 1 eq) and MeOH (14 ml, HPLC grade) were added to a 100-ml RBF. Then, the mixture was cooled to 0 °C with an ice/water bath, and sodium borohydride (175.2 mg, 4.63 mmol, 1.51 eq) was added to the reaction mixture in one portion. The resulting solution was stirred at 0 °C for 4 hours. TLC after 4 hours indicated the disappearance of the starting material. Saturated solution of NH_4Cl was added to the mixture and a milky white precipitate formed. The cooling bath was removed, and the mixture was stirred at RT for 1 hour. The flask was then stored in the fridge for 2 hours. The white precipitate was filtered out, washed with water (50 ml) and dried *in vacuo* (780 mg, 74%).

^{1}H NMR (500 MHz, $CDCl_3$): δ = 7.88 (s, 2H; Ar-H), 4.85 (d, *J* = 5.8 Hz, 2H; $-CH_2$), 1.96 (t, *J* = 5.8 Hz, 1H; -OH), 1.58 (s, 9H; $-C(CH_3)_3$). **^{13}C{^{1}H} NMR** (125 MHz, $CDCl_3$): δ = 163.9, 143.3, 131.4 (br), 129.0 (q, *J* = 32.3 Hz), 127.6 (q, *J* = 4.6 Hz), 123.2 (q, *J* = 272.8 Hz), 84.8, 63.5, 27.7. **^{19}F NMR** (471 MHz, $CDCl_3$): δ = −59.20. **HRMS** (APCI): m/z calcd for $C_{14}H_{14}F_6O_3+Cl^-$: 379.0541 $[M+Cl]^-$; found: 379.0539.

2.6 Synthesis of *tert*-butyl 4-(((methylsulfonyl)oxy)methyl)-2,6-bis(trifluoromethyl)benzoate 1

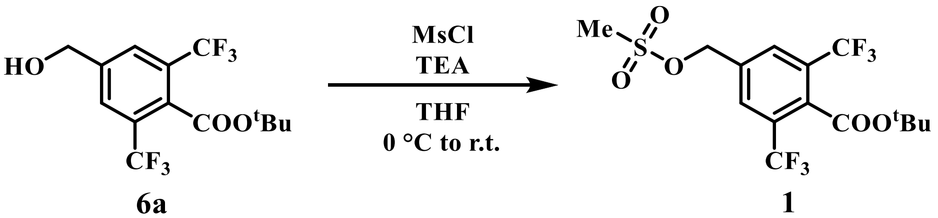

The starting alcohol **6a** (500 mg, 1.45 mmol, 1eq) was transferred to a 50-ml RBF. The flask was flushed with argon followed by addition THF (5 ml, anhydrous), giving a clear yellow solution. Triethylamine (300 µl) was added in one portion. The mixture was cooled to 0 °C with an ice bath. After that, methanesulfonyl chloride (170 µl, 2 mmol, 1.49 eq) was added in one portion, giving a milky yellow solution and the solution was stirred at room temperature. After 3 hours TLC indicated the disappearance of the starting material. The reaction mixture was concentrated on rotary evaporator to give yellow paste. The mixture was diluted with saturated solution of NH_4Cl followed by extraction with EtOAc (3×30 ml). Combined organic layers were dried over anhydrous magnesium sulphate, the drying agent was filtered off and the filtrate was concentrated on rotary evaporator to give a pale-yellow oil. The extract was dried *in vacuo*, giving a pale-yellow oil (490 mg, 80%).

^{1}H NMR (500 MHz, $CDCl_3$): δ = 7.91 (s, 2H; Ar-H), 5.31 (s, 2H; $-CH_2$), 3.09 (s, 3H; $-CH_3$), 1.58 (s, 9H; -$C(CH_3)_3$). **^{13}C{^{1}H} NMR** (125 MHz, $CDCl_3$): δ = 163.2, 136.2, 133.0 (br), 129.7 (q, J = 32.6 Hz), 129.4 (q, J = 4.7 Hz), 122.8 (q, J = 273.1 Hz), 85.3, 68.1, 38.4, 27.6. **^{19}F NMR** (471 MHz, $CDCl_3$): δ = −59.31.

2.7 Synthesis of glycoluril (3)

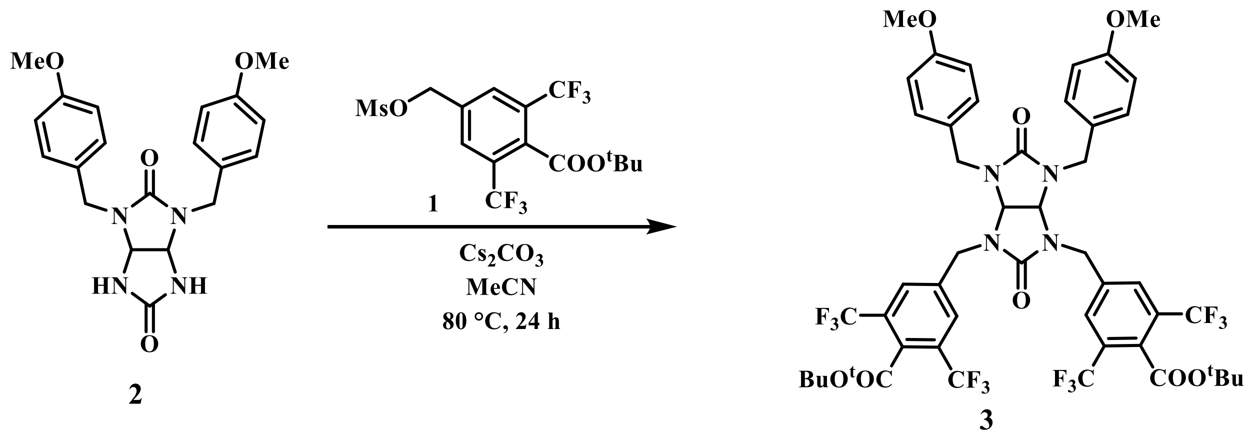

A mixture of glycoluril **2** (700 mg, 1.83 mmol, 1 eq), Cs_2CO_3 (1.5 g, 4.76 mmol, 2.60 eq), and CH_3CN (24 ml) was stirred at 70 °C for 1 h, and then *tert*-butyl 4-(((methylsulfonyl)oxy)methyl)-2,6-bis(trifluoromethyl)benzoate **1** (1.7 g, 4.03 mmol, 2.2 eq) as solution in acetonitrile (6 ml) was added. The mixture was stirred at 70 °C for 24 h. Then, the reaction mixture was filtered through a celite pad. The filtrate was concentrated, and flash column chromatography was performed [DCM/MeOH (0.2:1)] to yield white solid (1.6 g, 85%).

^{1}H NMR (500 MHz, CD_3CN): δ = 7.71 (s, 4H; Ar-H), 7.04-7.01 (m, 4H; Ar-H), 6.81-6.78 (m, 4H; Ar-H), 5.06 (s, 2H; -CH), 4.60 (d, J = 5.2 Hz, 2H; $-CH_2$), 4.56 (d, J = 4.2 Hz, 2H; $-CH_2$), 4.36 (d, J = 16.8 Hz, 2H; $-CH_2$), 4.2 (d, J = 15.9 Hz, 2H; $-CH_2$), 3.74 (s, 6H; $-CH_3$), 1.55 (s, 18H; -$C(CH_3)_3$). **^{13}C{^{1}H} NMR** (125 MHz, CD_3CN): δ = 164.6, 160.5, 160.2, 159.9, 141.9, 131.9 (br), 130.0 (q, J = 4.7 Hz),129.72, 129.68, 129.0 (q, J = 32.3 Hz), 124.2 (q, J = 272.5 Hz), 115.0, 85.7, 70.1, 55.8, 47.5, 46.9, 27.8. **^{19}F NMR** (471 MHz, CD_3CN): δ = −59.61. **HRMS** (APCI): m/z calcd for $C_{40}H_{31}F_{12}N_4O_8$+ H^+: 923.1950 $[M+H]^+$; found: 923.1937.

2.8 Synthesis of glycoluril (4)

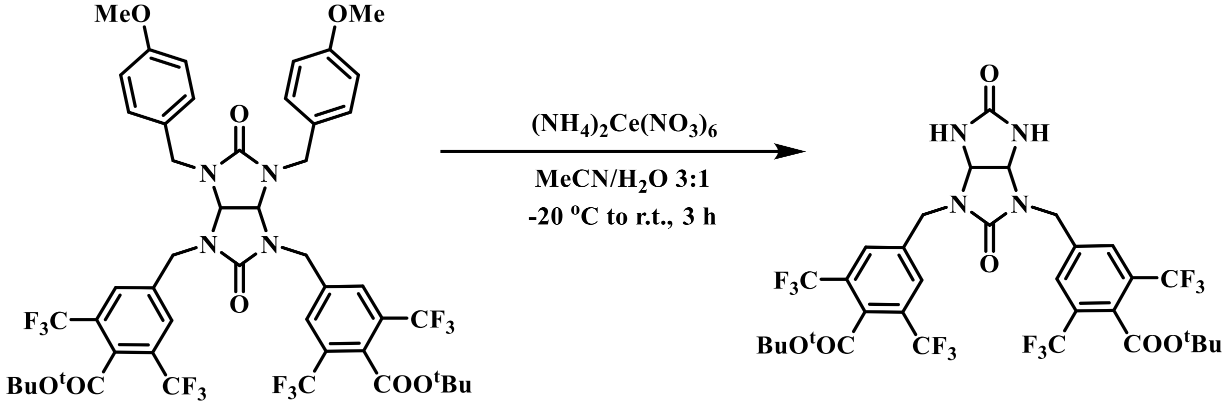

3 4

A cold solution of $(NH_4)_2Ce(NO_3)_6$ (4.2 g, 7.73 mmol, 4 eq) in H_2O (13.26 ml) was added dropwise over 30 min to a solution of **3** (2 g, 1.93 mmol, 1 eq) in CH_3CN (40 ml) at −20 °C. The mixture was stirred at room temperature for 3 h, and then CH_3CN was removed via evaporation under reduced pressure. Water (20 ml) was added, and the aqueous phase was extracted with EtOAc (3×30 ml). The collected organic layers were washed with brine (10 ml), dried over anhydrous magnesium sulphate, filtered, and concentrated under reduced pressure. Flash column chromatography was performed [DCM/MeOH (0.2:1)] to obtain **4** as a white solid (690 mg, 52 % yield).

^{1}H NMR (500 MHz, CD_3CN): δ = 7.96 (s, 4H; Ar-H), 6.00 (s, 2H; -NH), 5.22 (s, 2H; -CH), 4.66 (d, J = 16.4 Hz, 2H; $-CH_2$), 4.45 (d, J = 16.4 Hz, 2H; $-CH_2$) 1.55 (s, 18H; $-C(CH_3)_3$). **^{13}C{^{1}H} NMR** (125 MHz, CD_3CN): δ = 164.7, 161.6, 159.2, 142.4, 131.8 (br), 130.7 (q, J = 4.6 Hz), 129.0 (q, J = 32.1 Hz), 124.3 (q, J = 272.0 Hz), 85.7, 67.8, 45.5, 27.8. **^{19}F NMR** (471 MHz, CD_3CN): δ = −59.63.

2.9 Synthesis of glycoluril (5)

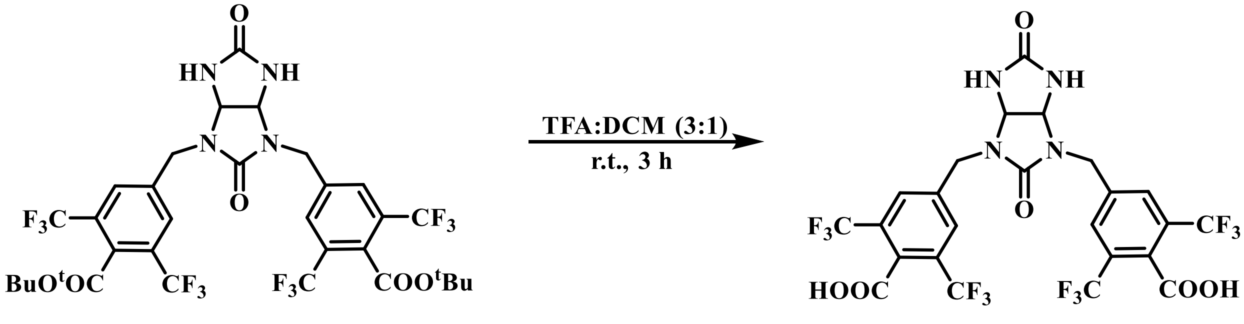

4 5

Glycoluril **4** (200 mg, 0.252 mmol) was dissolved in TFA:dichloromethane mixture (3:1, 3 ml) and stirred for 3 h at room temperature. After evaporating the solvent on rotary evaporator, the white solid was co-

evaporated with dichloromethane (2×5 ml) and toluene (5 ml) and dried in vacuo to obtain glycoluril **5** in quantitative yield.

^{1}H NMR (500 MHz, Acetone-d$_6$): δ = 8.10 (s, 4H; Ar-H), 5.58 (s, 2H; -NH), 4.88 (d, *J* = 16.3 Hz, 2H; -CH$_2$), 4.65 (d, *J* = 16.2 Hz, 2H; -CH$_2$). **^{13}C{^{1}H} NMR** (125 MHz, Acetone-d$_6$): δ = 166.4, 161.5, 159.1, 142.6, 131.3 (br), 130.6 (q, *J* = 4.6 Hz), 129.1 (q, *J* = 32.4 Hz), 124.1 (q, *J* = 272.0 Hz), 67.7, 45.4. **^{19}F NMR** (471 MHz, Acetone-d$_6$): δ = −59.99. **HRMS** (APCI): m/z calcd for $C_{24}H_{14}F_{12}N_4O_6+H^+$: 683.0794 [M+H]$^+$; found: 683.0790.

2.10 Synthesis of Bambusuril (BU2)

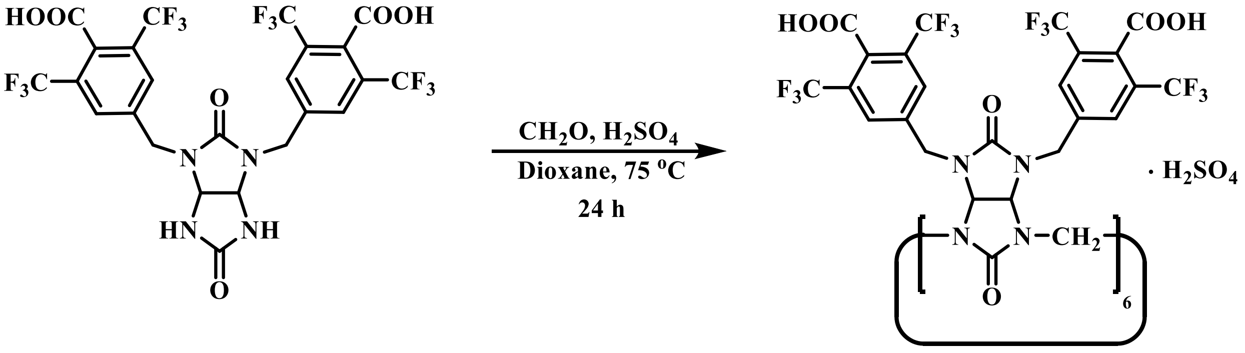

5 **BU2**

Paraformaldehyde (11 mg, 0.36 mmol, 1.45 eq) and the starting glycoluril **5** (171 mg, 0.25 mmol, 1 eq) were transferred to a 5 ml RBF. Dioxane (500 µl, anhydrous) was added, giving a beige suspension. Sulphuric acid (42 µl) was added to the reaction mixture which immediately turned into transparent brown solution. The resulting solution was stirred at 75 °C for 24 hours. The reaction mixture was cooled down to room temperature and milliQ water was added. Beige coloured precipitate was filtered and obtained solid was washed with milliQ water twice. Precipitate was washed with diethyl ether (2×10 ml), dichloromethane (2×10 ml) and pentane (2×10 ml) to obtain pure product (120 mg, 70 % yield).

^{1}H NMR (500 MHz, Acetone-d$_6$): δ = 8.15 (s, 24H; Ar-H), 6.02 (s, 12H; -CH), 5.18 (dd, *J* = 16.7, 38.7 Hz 24H; -CH$_2$), 4.59 (s, 12H; -CH$_2$). **^{13}C{^{1}H} NMR** (125 MHz, Acetone-d$_6$): δ = 166.2, 161.9, 160.5, 143.6, 131.1 (br), 129.42 (q, *J* = 32.4 Hz), 129.4 (br), 124.0 (q, *J* = 272.0 Hz), 71.8, 48.8. **^{19}F NMR** (471 MHz, Acetone-d$_6$): δ = −60.23. **MALDI-TOF-MS, DHB**: m/z calcd for $[C_{150}H_{86}F_{72}N_{24}O_{40}S-H]^-$: 4261.393; found: 4261.472.

3. Isothermal Titration Calorimetry (ITC) Studies

The host–guest complexation between Cl^- and HSO_4^-⊂BU2 was studied by direct titration of TBA^+Cl^- into a solution of HSO_4^-⊂BU2. The other host–guest complexes were studied using competitive ITC titrations, in which the anion of interest was added to a HSO_4^-⊂BU2 solution already containing a competing anion (Cl^- or Br^-). Thus, two- or three-step competition models were used to evaluate these complexes. To account for propagated error, each second-stage titration was refitted twice—once using the lower bound (K_d-low) and once using the upper bound (K_d-up) of the reference K_d—and the resulting variation was reported. The obtained K_d values were then converted to the corresponding K_a values, and the propagated errors were reported as standard deviations.

Table S1. Apparent dissociation (K_d) and association (K_a) constants of the anion⊂BU2 complexes determined by ITC in 30 mM aq. K_2HPO_4 (pH 7.1) at 298.15 K

**binding of chloride**		
	K_d (M)	K_a (M^{-1})
Cl-1	2.22E-05	4.50E+04
Cl-2	2.24E-05	4.46E+04
Cl-3	2.17E-05	4.61E+04
final value		$(4.5\pm0.1)\times10^4$
**binding of bromide (by competition with chloride)**		
	K_d (M)	K_a (M^{-1})
Br-1-up	3.30E-08	3.03E+07
Br-1-low	3.21E-08	3.12E+07
Br-2-up	3.22E-08	3.11E+07
Br-2-low	3.13E-08	3.19E+07
final value		$(3.1\pm0.1)\times10^7$
**binding of iodide (by competition with bromide)**		
	K_d (M)	K_a (M^{-1})
I-1-up	5.50E-11	1.82E+10
I-1-low	5.28E-11	1.89E+10
I-2-up	6.61E-11	1.51E+10
I-2-low	6.34E-11	1.58E+10
final value		$(1.7\pm0.2)\times10^{10}$
**binding of perchlorate (by competition with bromide)**		
	K_d (M)	K_a (M^{-1})
ClO_4-1-up	1.05E-10	9.52E+09
ClO_4-1-low	1.01E-10	9.90E+09
ClO_4-2-up	1.00E-10	1.00E+10
ClO4-2-low	9.61E-11	1.04E+10

final value		$(9.9\pm0.4)\times10^{9}$
**binding of BF_4^- (by competition with bromide)**		
	K_d (M)	K_a (M^{-1})
BF_4-1-up	4.32E-10	2.31E+09
BF4-1-low	4.14E-10	2.42E+09
BF_4-2-up	4.40E-10	2.27E+09
BF4-2-low	4.23E-10	2.36E+09
final value		$(2.3\pm0.1)\times10^{9}$
**binding of PF_6^- (by competition with bromide)**		
	K_d (M)	K_a (M^{-1})
PF_6-1-up	1.23E-09	8.13E+08
PF6-1-low	1.18E-09	8.47E+08
PF6-2-up	1.24E-09	8.06E+08
PF6-2-low	1.19E-09	8.40E+08
final value		$(8.3\pm0.2)\times10^{8}$
**binding of ReO_4^- (by competition with chloride)**		
	K_d (M)	K_a (M^{-1})
ReO_4-1-up	7.56E-08	1.32E+07
ReO4-1-low	7.35E-08	1.36E+07
ReO4-2-up	8.25E-08	1.21E+07
ReO4-2-low	8.02E-08	1.25E+07
final value		$(1.3\pm0.1)\times10^{7}$
**binding of SCN^- (by competition with chloride)**		
	K_d (M)	K_a (M^{-1})
SCN-1-up	2.98E-08	3.36E+07
SCN-1-low	2.90E-08	3.45E+07
SCN-2-up	3.08E-08	3.25E+07
SCN-2-low	3.00E-08	3.33E+07
final value		$(3.3\pm0.1)\times10^{7}$
**binding of NO_3^- (by competition with chloride)**		
	K_d (M)	K_a (M^{-1})
NO_3-1-up	1.59E-08	6.29E+07
NO_3-1-low	1.55E-08	6.45E+07
NO_3-2-up	1.79E-08	5.59E+07
NO_3-2-low	1.75E-08	5.71E+07
final value		$(6.0\pm0.4)\times10^{7}$
**binding of F^- (by competition with chloride)**		
	K_d (M)	K_a (M^{-1})

F-1-up	2.83E-03	3.53E+02
F-1-low	2.31E-03	4.33E+02
F-2-up	2.47E-03	4.05E+02
F-2-low	2.05E-03	4.88E+02
final value		$(4.1\pm0.6)\times10^2$
**binding of N_3^-**		
	K_d (M)	K_a (M^{-1})
N_3-1	1.15E-06	8.70E+05
N_3-2	1.23E-06	8.13E+05
final value		$(8.4\pm0.4)\times10^5$

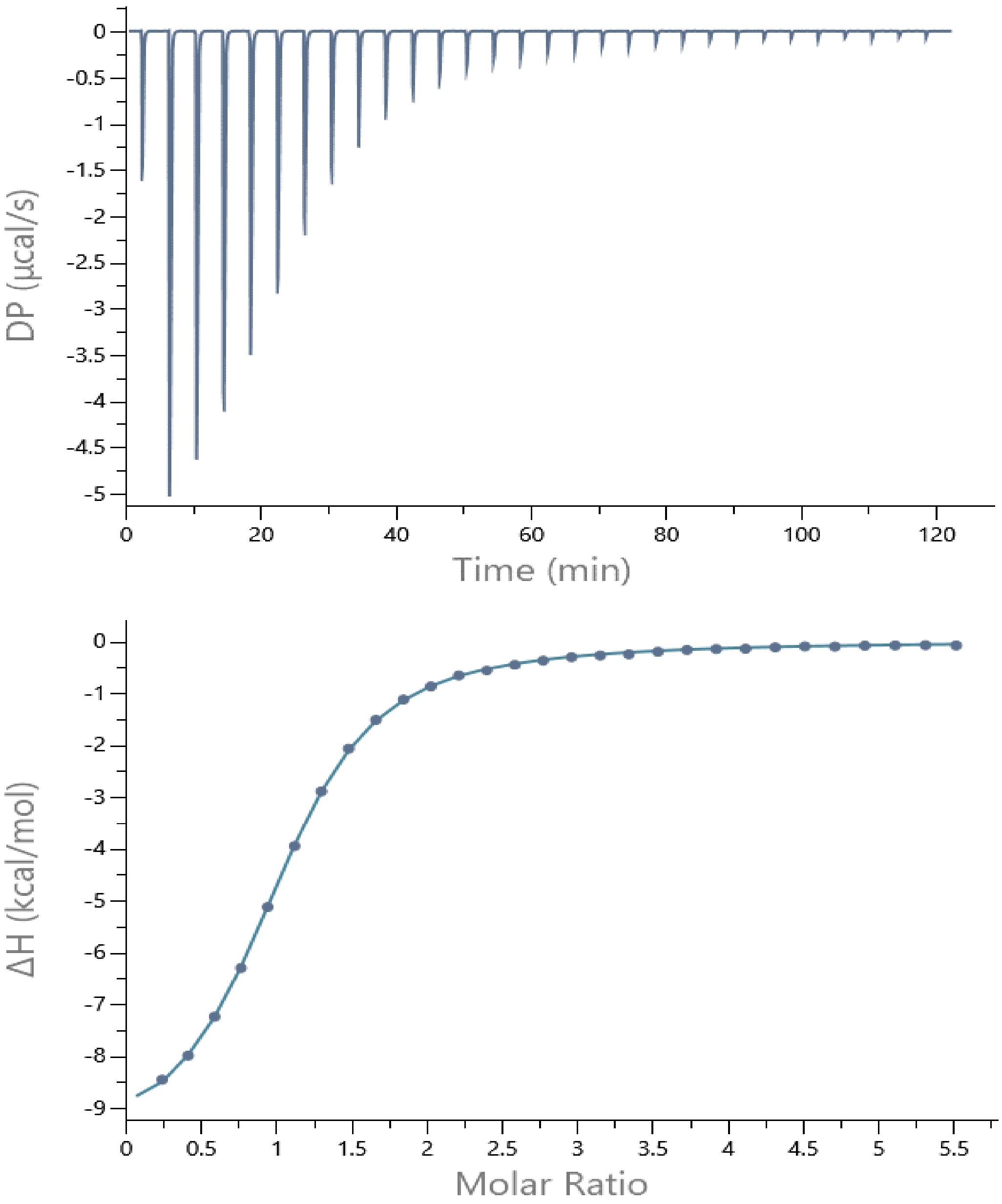

Figure S1. Reverse ITC titration for weaker binding of HSO_4^-⊂BU2 (0.2 mM) with sodium fluoride (5.45 mM) in the presence of sodium chloride (0.6 mM) in 30 mM aq. K_2HPO_4 (pH 7.1) at 298.15 K.

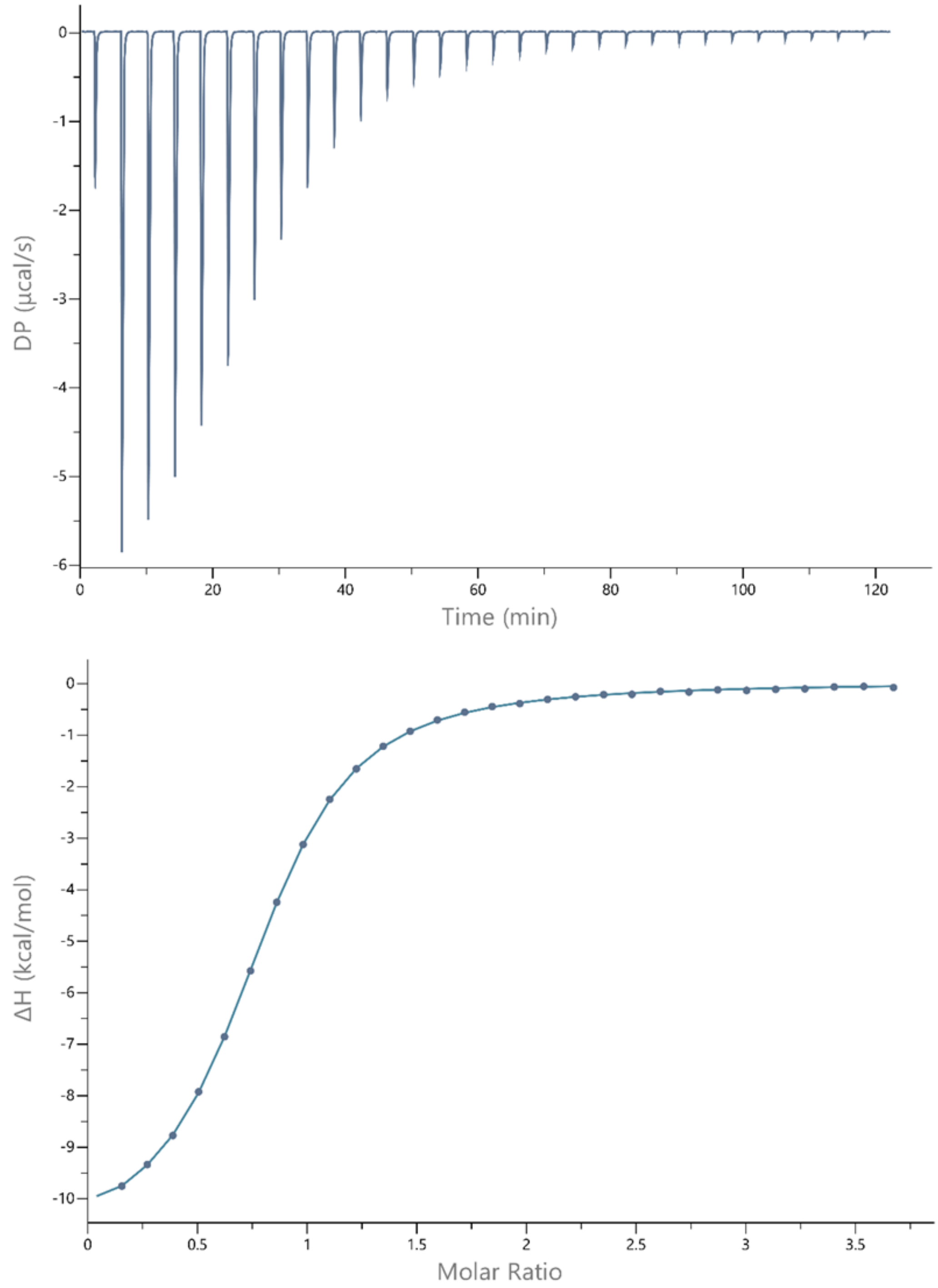

Figure S2. ITC titration of HSO_4^-⊂BU2 (0.3 mM) with sodium chloride (5.45 mM) in 30 mM aq. K_2HPO_4 (pH 7.1) at 298.15 K.

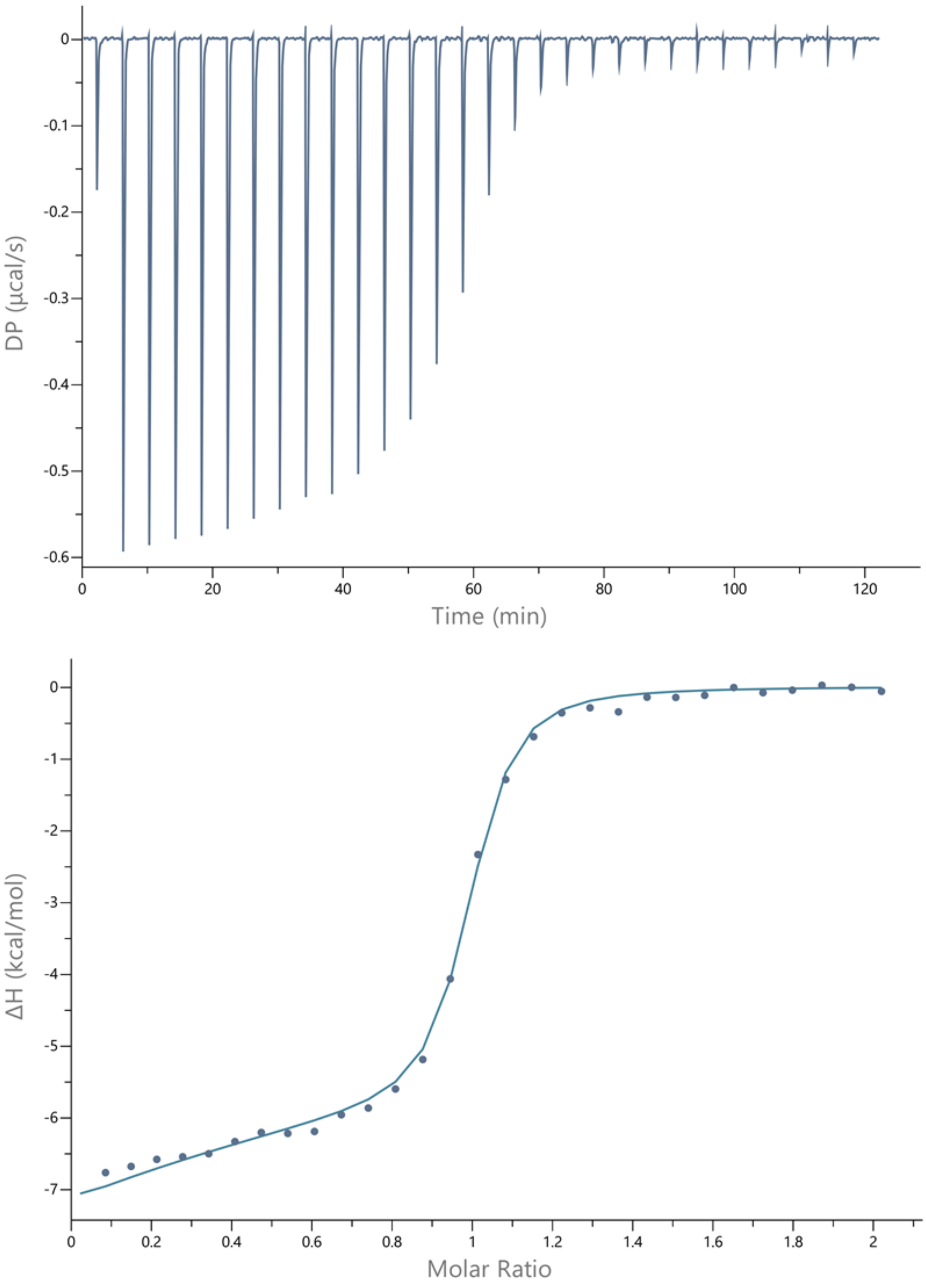

Figure S3. ITC titration of HSO_4^-⊂BU2 (0.075 mM) with sodium bromide (0.75 mM) in the presence of sodium chloride (0.15 mM) in 30 mM aq. K_2HPO_4 (pH 7.1) at 298.15 K.

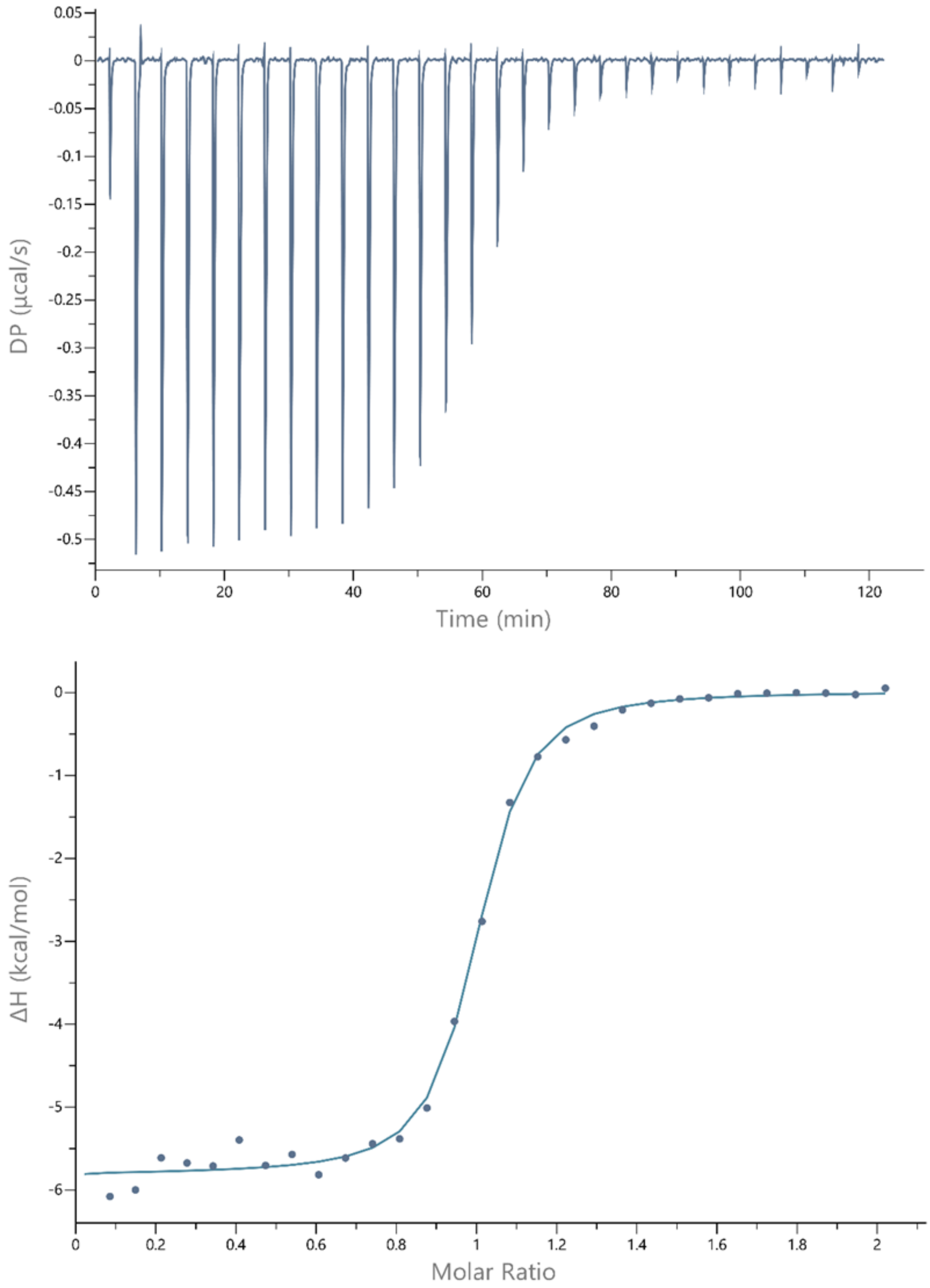

Figure S4. ITC titration of HSO_4^-⊂BU2 (0.075 mM) with sodium iodide (0.75 mM) in the presence of sodium bromide (0.15 mM) in 30 mM aq. K_2HPO_4 (pH 7.1) at 298.15 K.

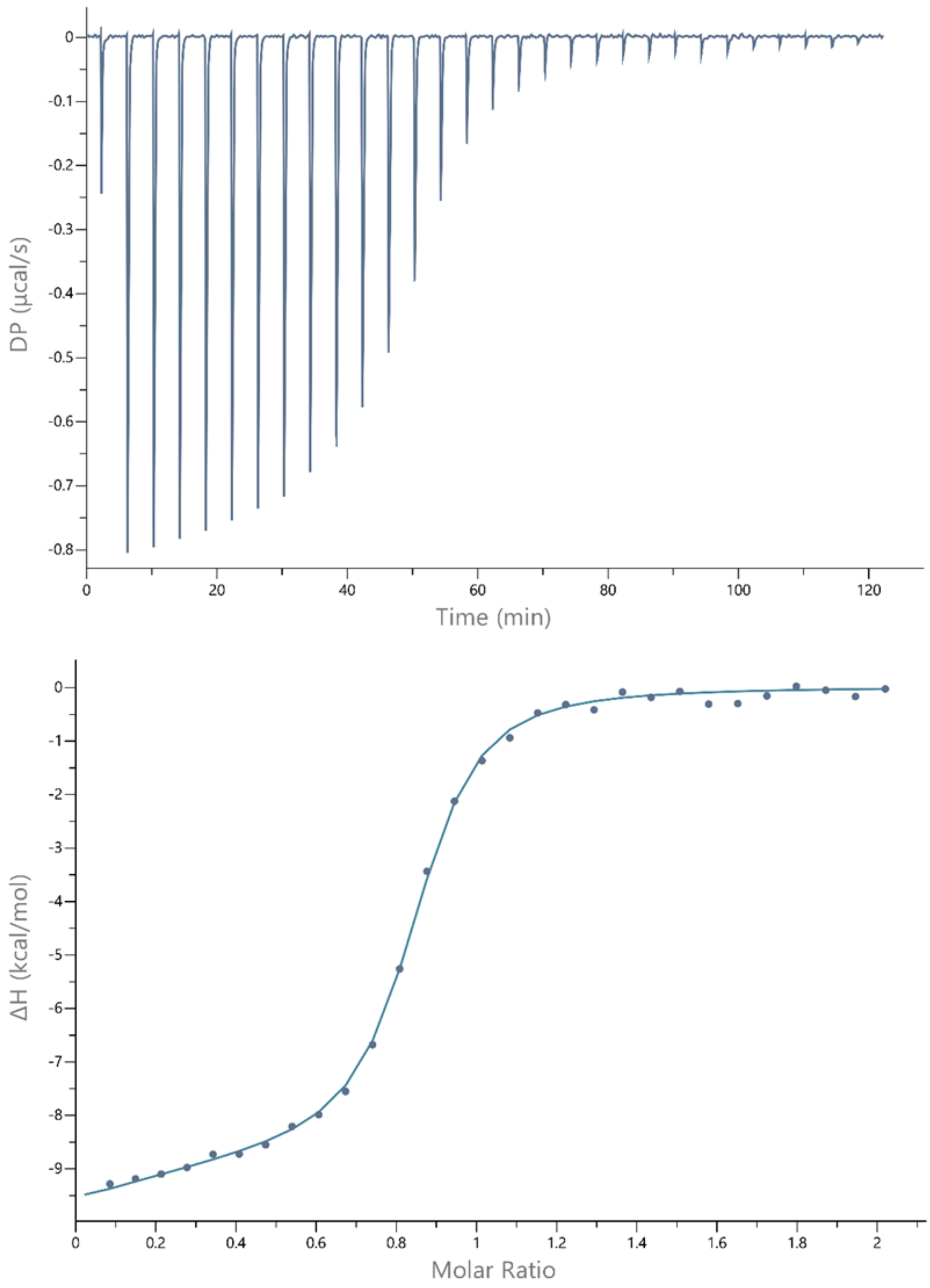

Figure S5. ITC titration of HSO_4^-⊂BU2 (0.075 mM) with sodium perrhenate (0.75 mM) in the presence of sodium chloride (0.15 mM) in 30 mM aq. K_2HPO_4 (pH 7.1) at 298.15 K.

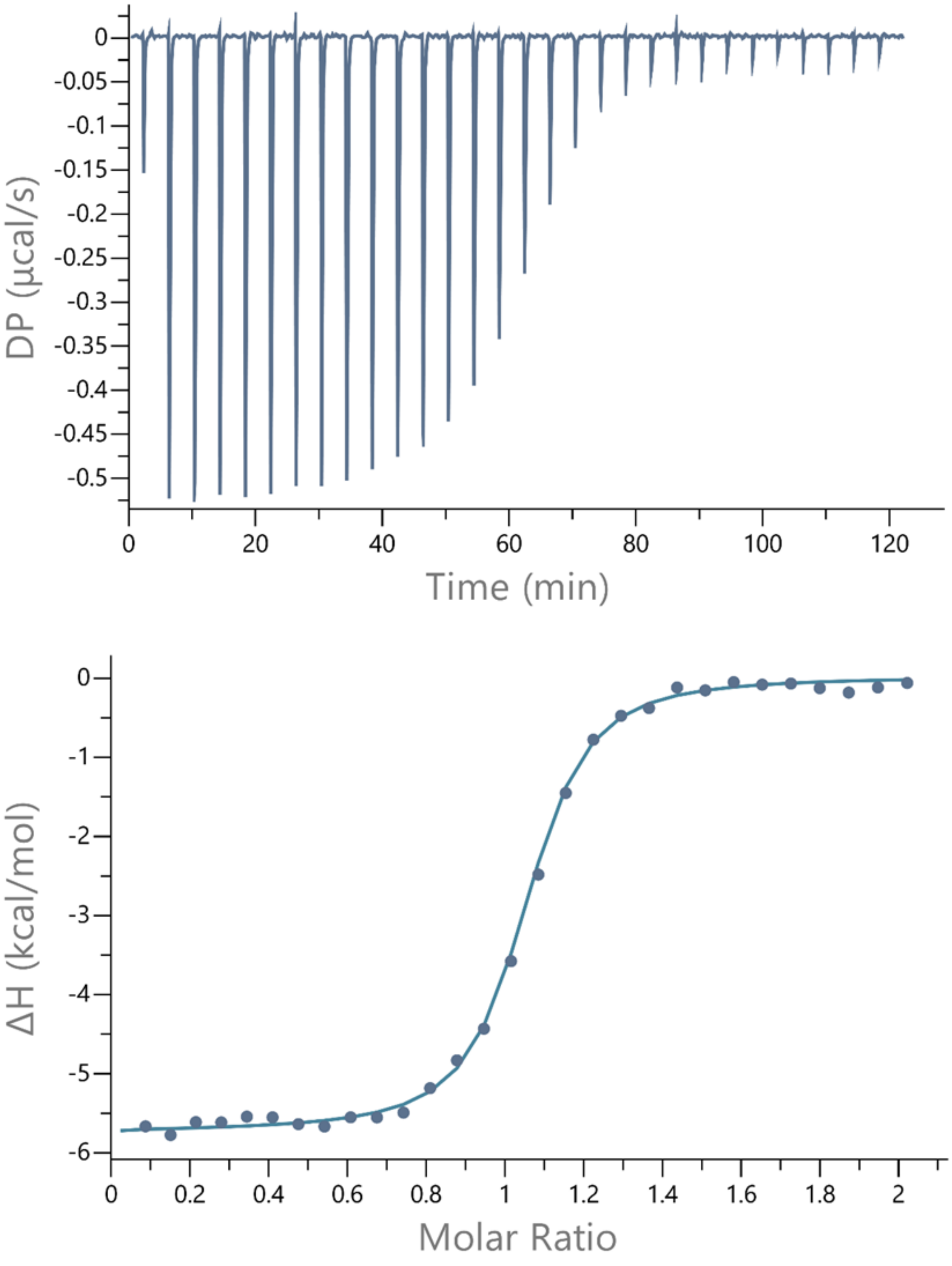

Figure S6. ITC titration of HSO_4^-⊂BU2 (0.075 mM) with sodium perchlorate (0.75 mM) in the presence of sodium bromide (0.15 mM) in 30 mM aq. K_2HPO_4 (pH 7.1) at 298.15 K.

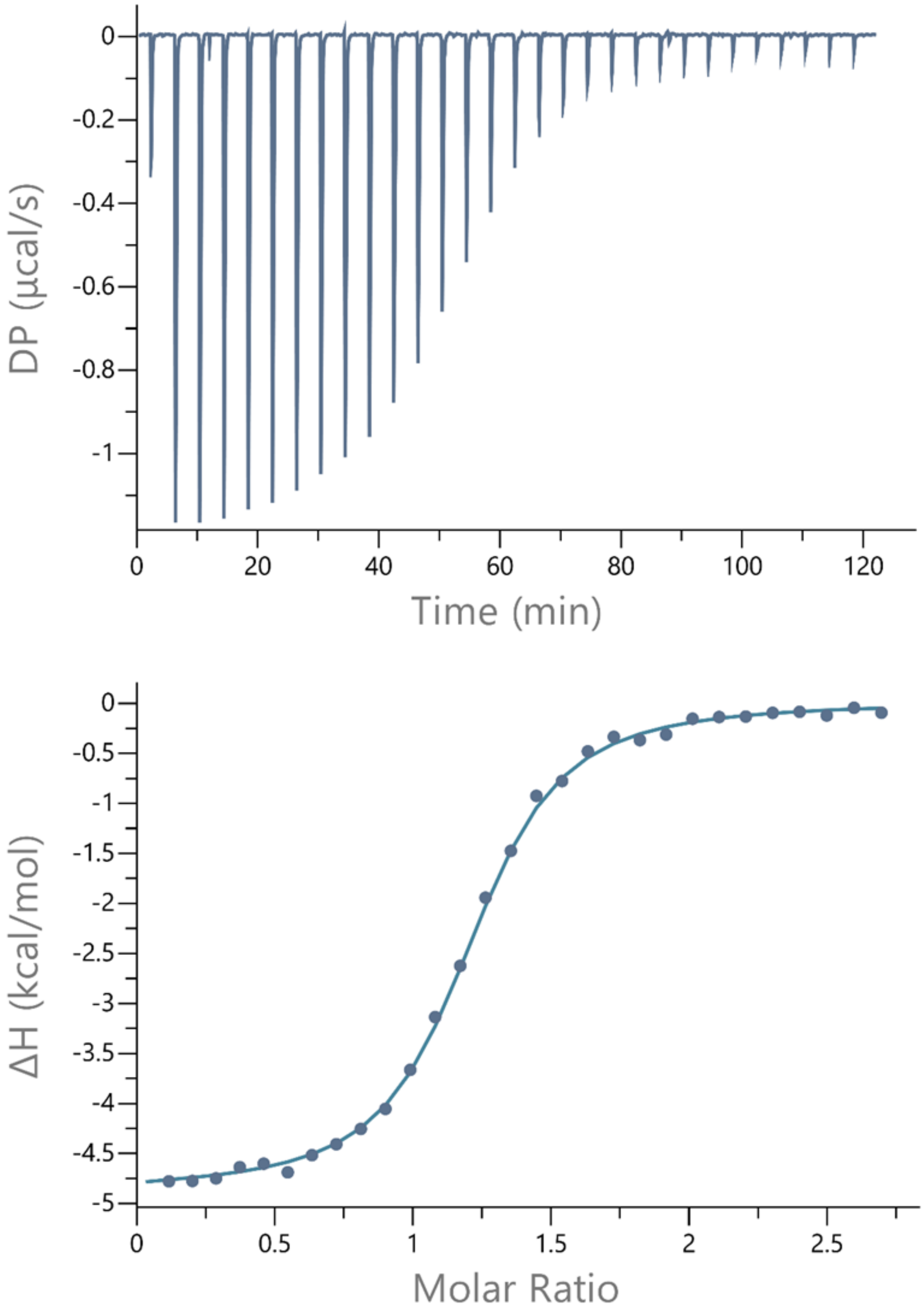

Figure S7. ITC titration of HSO_4^-⊂BU2 (0.15 mM) with sodium tetrafluoroborate (2.00 mM) in the presence of sodium bromide (0.3 mM) in 30 mM aq. K_2HPO_4 (pH 7.1) at 298.15 K.

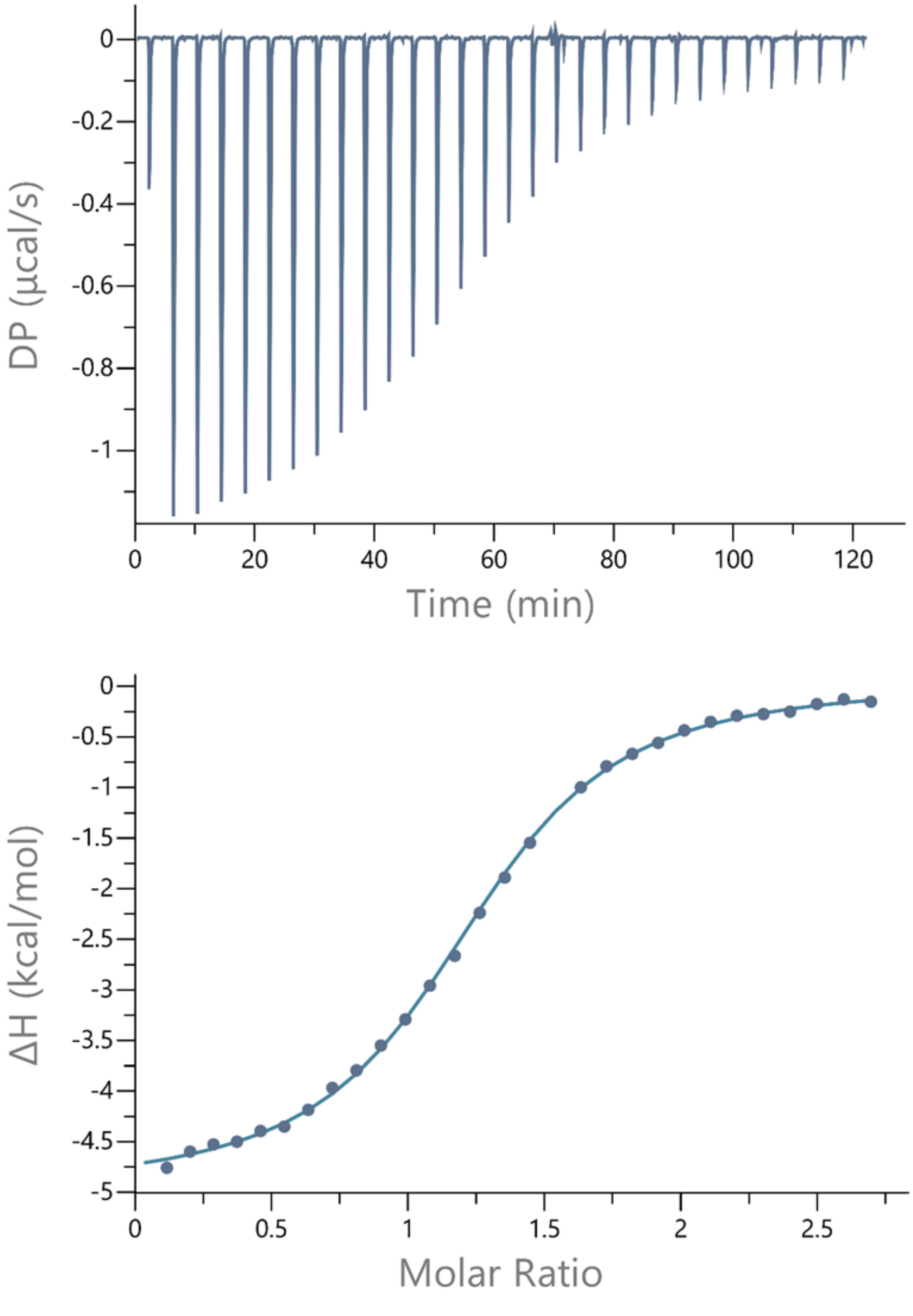

Figure S8. ITC titration of HSO_4^-⊂BU2 (0.15 mM) with sodium hexafluorophosphate (2.00 mM) in the presence of sodium bromide (0.3 mM) in 30 mM aq. K_2HPO_4 (pH 7.1) at 298.15 K.

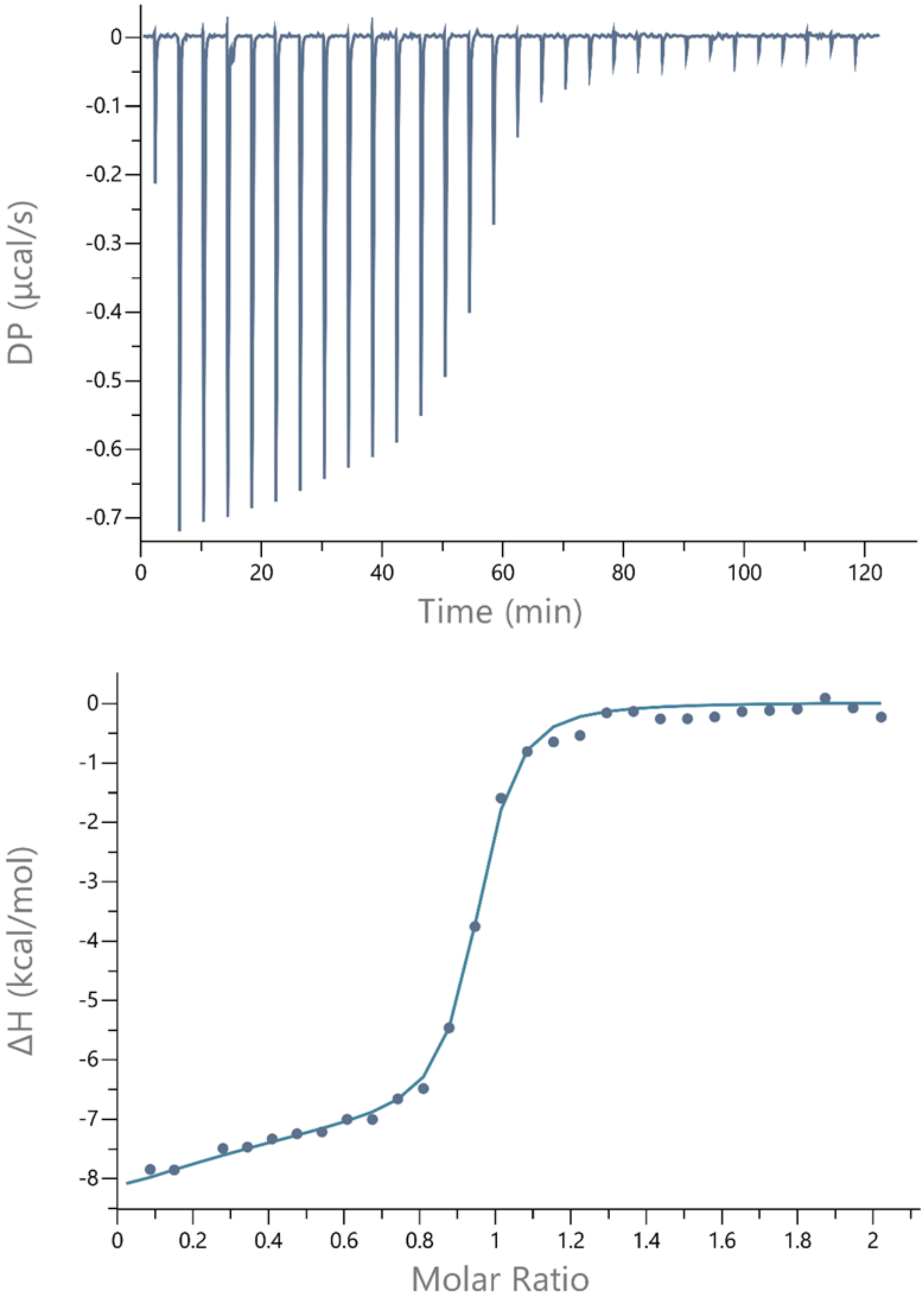

Figure S9. ITC titration of HSO_4^-⊂BU2 (0.075 mM) with sodium thiocyanate (0.75 mM) in the presence of sodium chloride (0.15 mM) in 30 mM aq. K_2HPO_4 (pH 7.1) at 298.15 K.

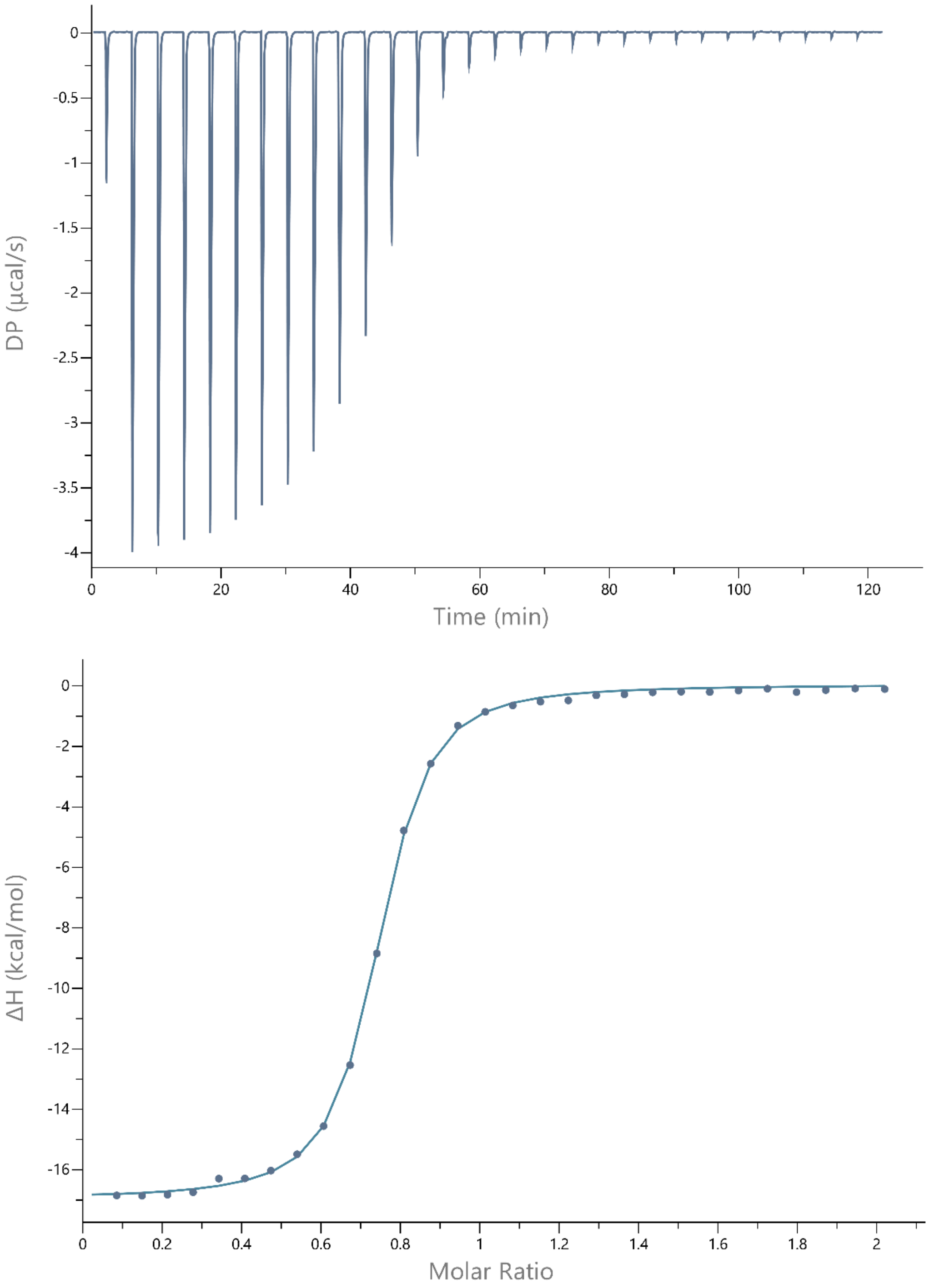

Figure S10. ITC titration of HSO_4^-⊂BU2 (0.075 mM) with sodium azide (0.75 mM) in 30 mM aq. K_2HPO_4 (pH 7.1) at 298.15 K.

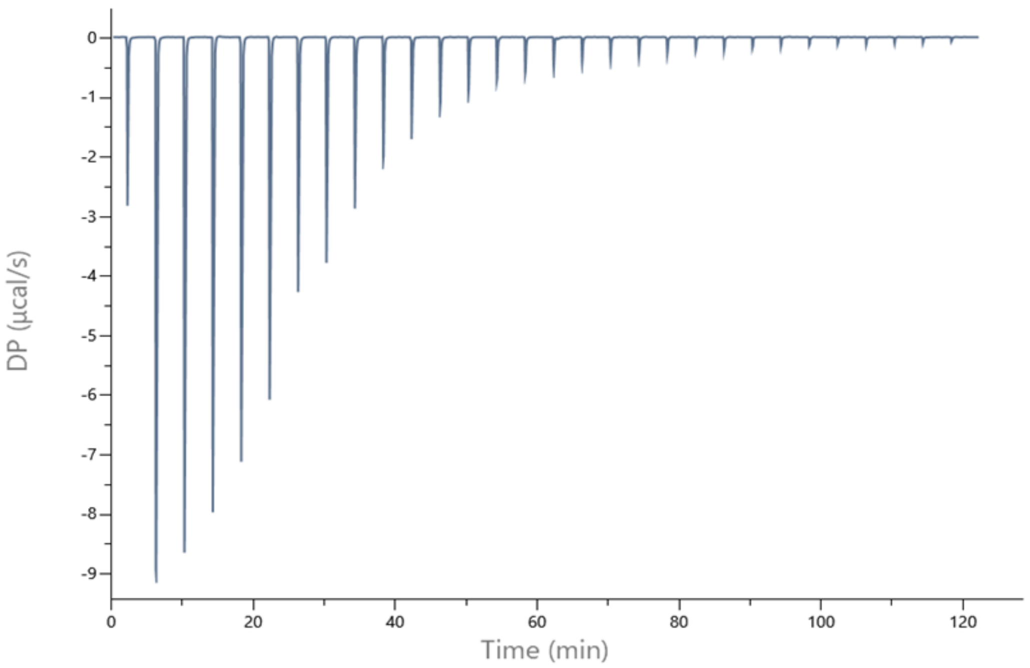

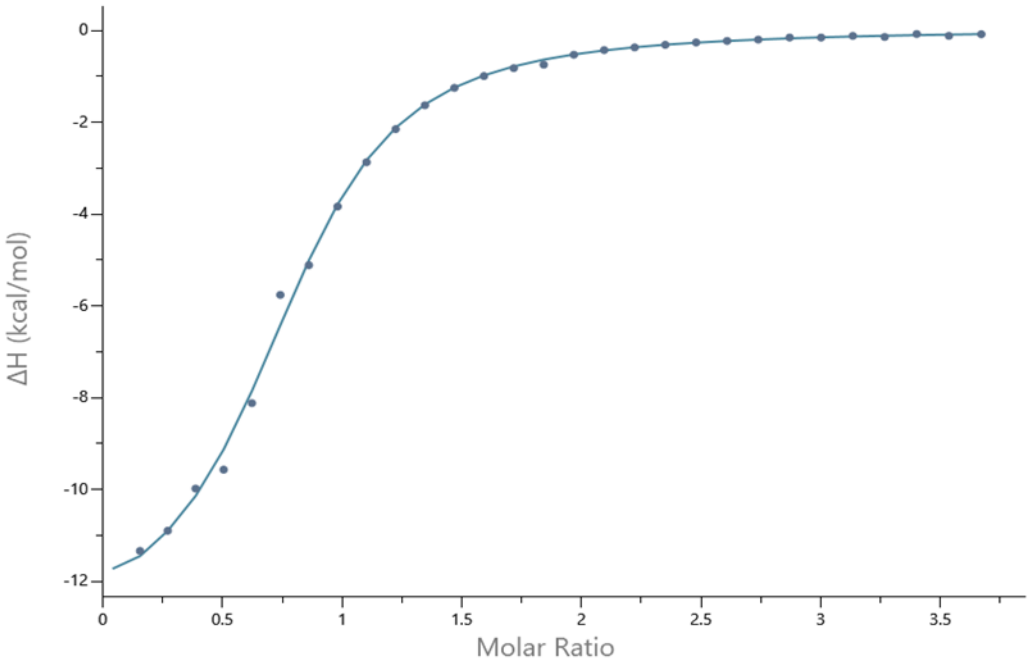

Figure S11. ITC titration of HSO_4^-⊂BU2 (0.3 mM) with sodium chloride (5.45 mM) in 10 mM aq. K_2HPO_4 (pH 7.1) at 298.15 K.

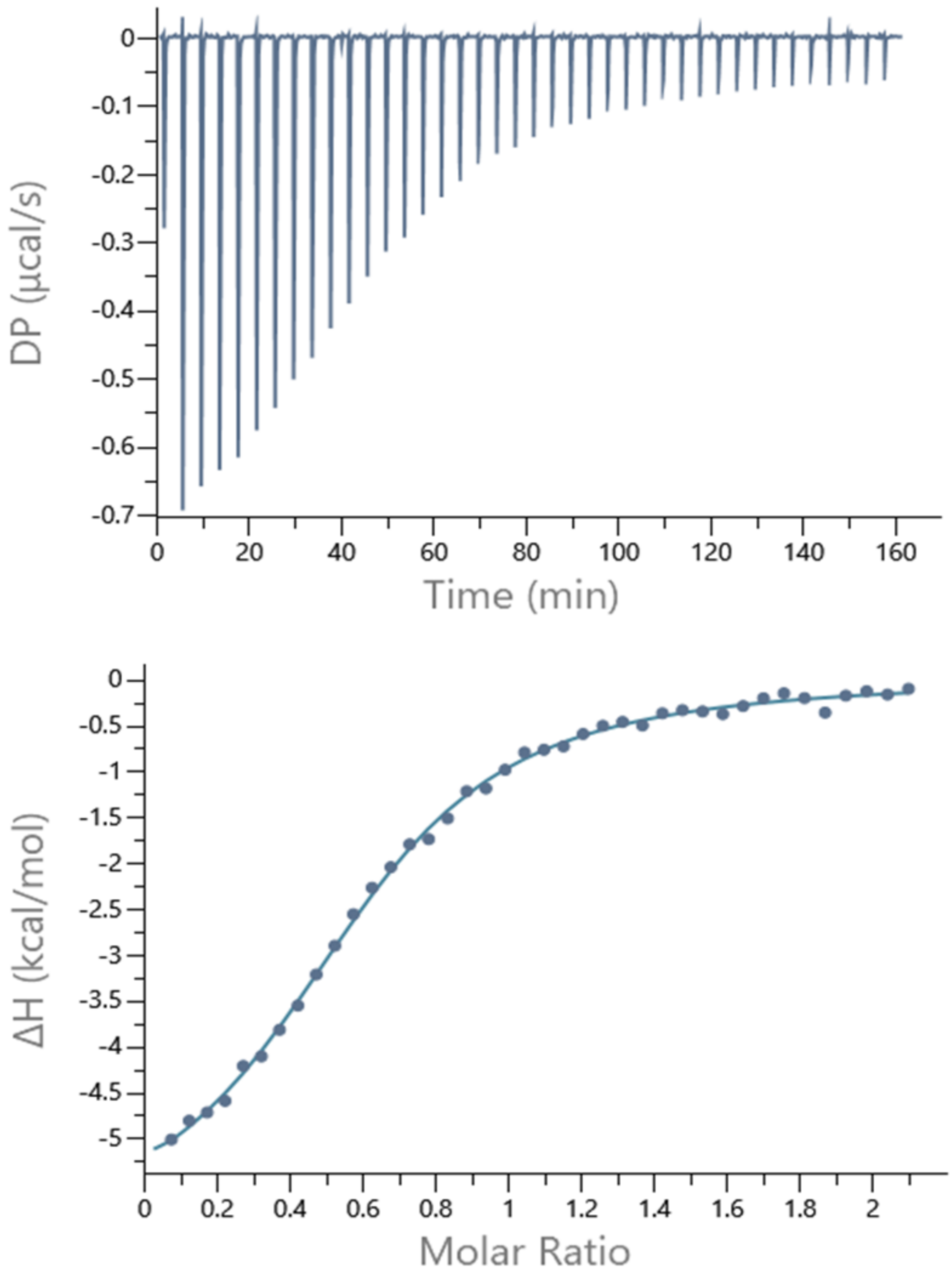

Figure S12. ITC titration of HSO_4^-⊂BU2 (0.150 mM) with sodium chloride (1.5 mM) in 300 mM aq. K_2HPO_4 (pH 7.1) at 298.15 K.

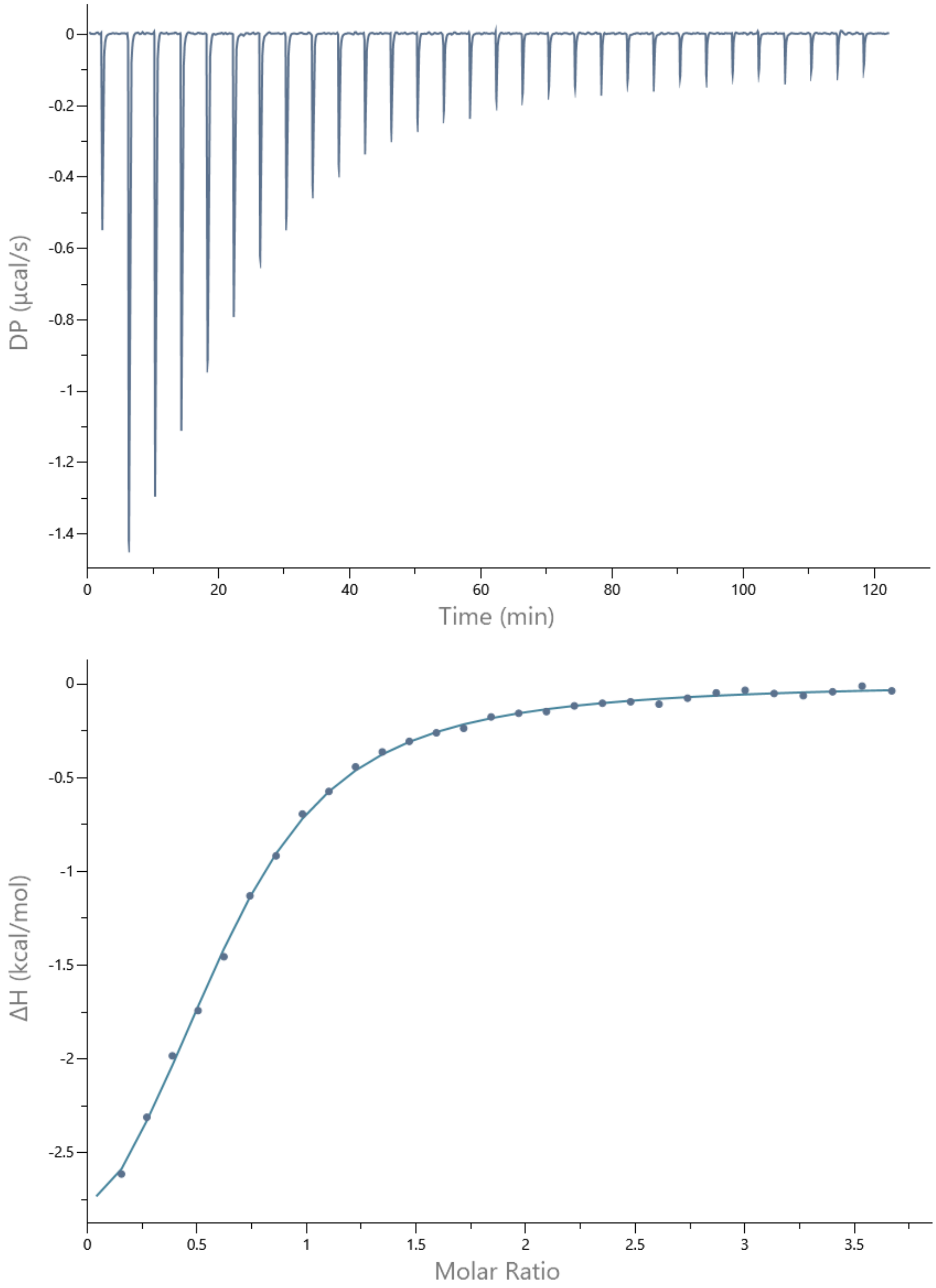

Figure S13. ITC titration of HSO_4^-⊂BU2 (0.3 mM) with sodium chloride (5.45 mM) in 1 M aq. K_2HPO_4 (pH 7.1) at 298.15 K.

4. NMR Titrations Showing Anion Binding of BU2

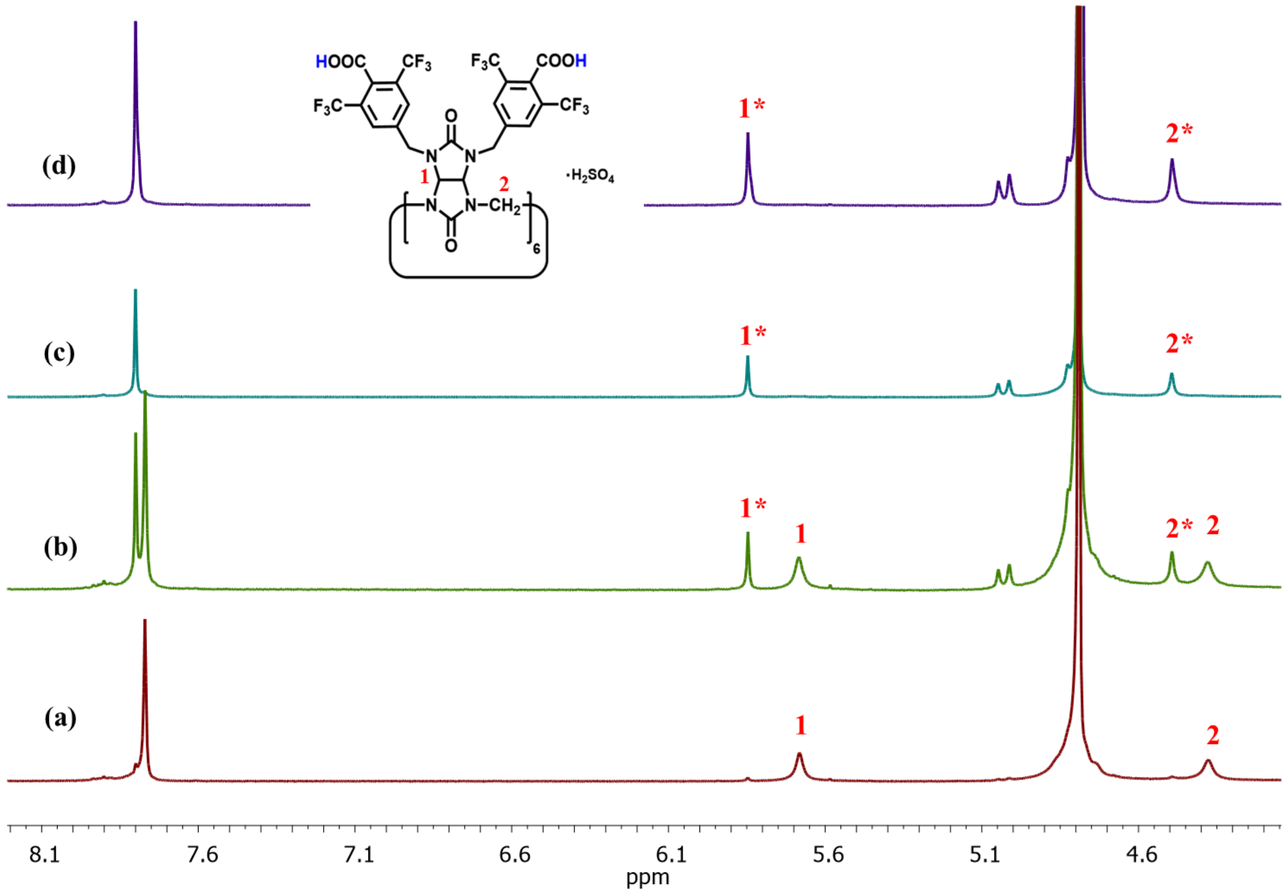

Figure S14: ^{1}H NMR (D_2O, 30 mM K_2DPO_4, 500 MHz) spectra of the HSO_4^-⊂BU2 complex (1 mM) (a) in the absence and in the presence of (b) 0.5 eq, (b) 1 eq, and (c) 1.2 eq of TMABr. Here, 1,2 are signals of the HSO_4^-⊂BU2 complex and 1*,2* are signals of the TMABr⊂ **BU2** complex.

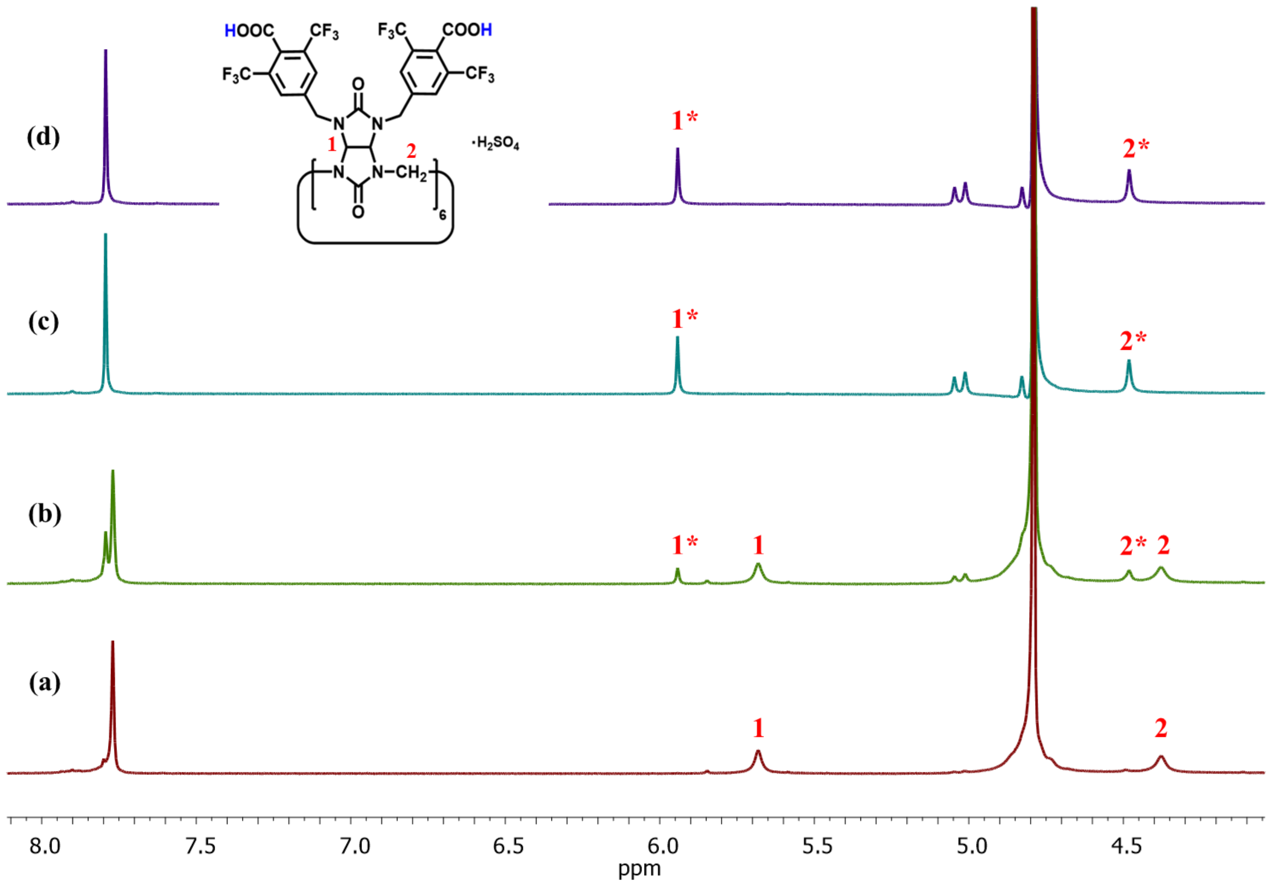

Figure S15: ^{1}H NMR (D_2O, 30 mM K_2DPO_4, 500 MHz) spectra of the HSO_4^-⊂BU2 complex (1 mM) (a) in the absence and in the presence of (b) 0.5 eq, (b) 1 eq, and (c) 1.2 eq of TMAI. Here, 1,2 are signals of the HSO_4^-⊂BU2 complex and 1*,2* are signals of the TMAI⊂ **BU2** complex.

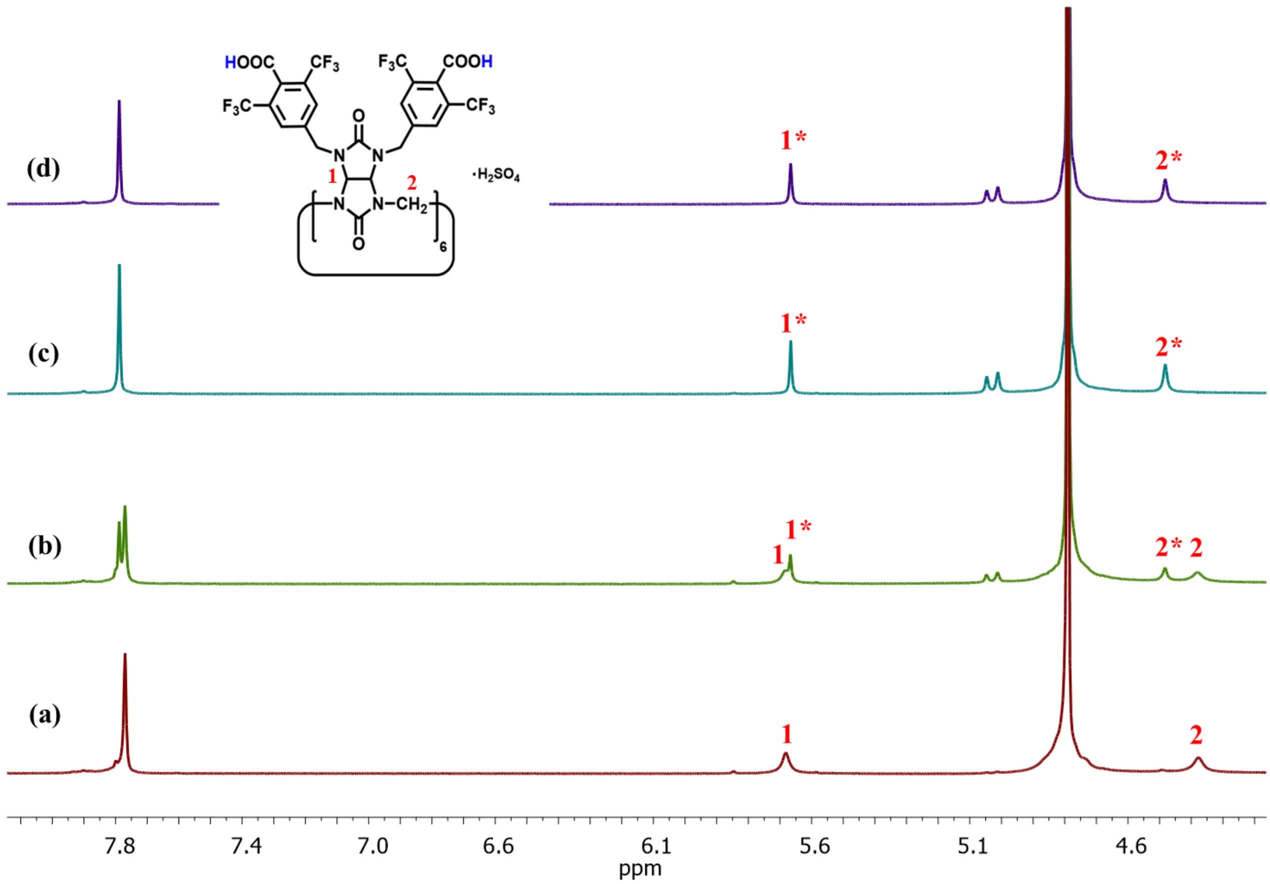

Figure S16: ^{1}H NMR (D_2O, 30 mM K_2DPO_4, 500 MHz) spectra of the $HSO_4^- \subset$BU2 complex (1 mM) (a) in the absence and in the presence of (b) 0.5 eq, (b) 1 eq, and (c) 1.2 eq of $TMANO_3$. Here, 1,2 are signals of the $HSO_4^- \subset$BU2 complex and 1*,2* are signals of the $TMANO_3 \subset$ **BU2** complex.

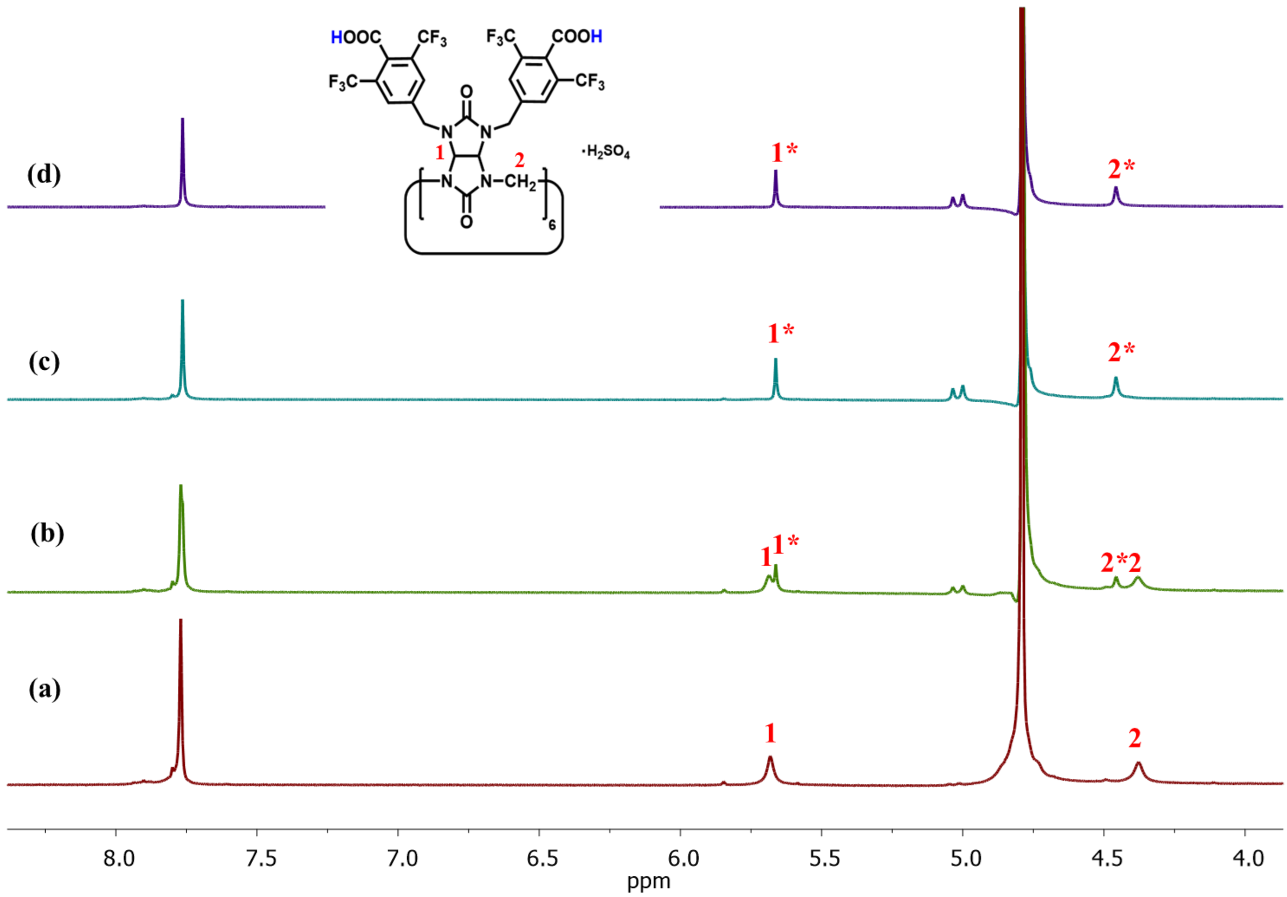

Figure S17: ^{1}H NMR (D_2O, 30 mM K_2DPO_4, 500 MHz) spectra of the HSO_4^-⊂BU2 complex (1 mM) (a) in the absence and in the presence of (b) 0.5 eq, (b) 1 eq, and (c) 1.2 eq of $TMAClO_4$. Here, 1,2 are signals of the HSO_4^-⊂BU2 complex and 1*,2* are signals of the $TMAClO_4$⊂ **BU2** complex.

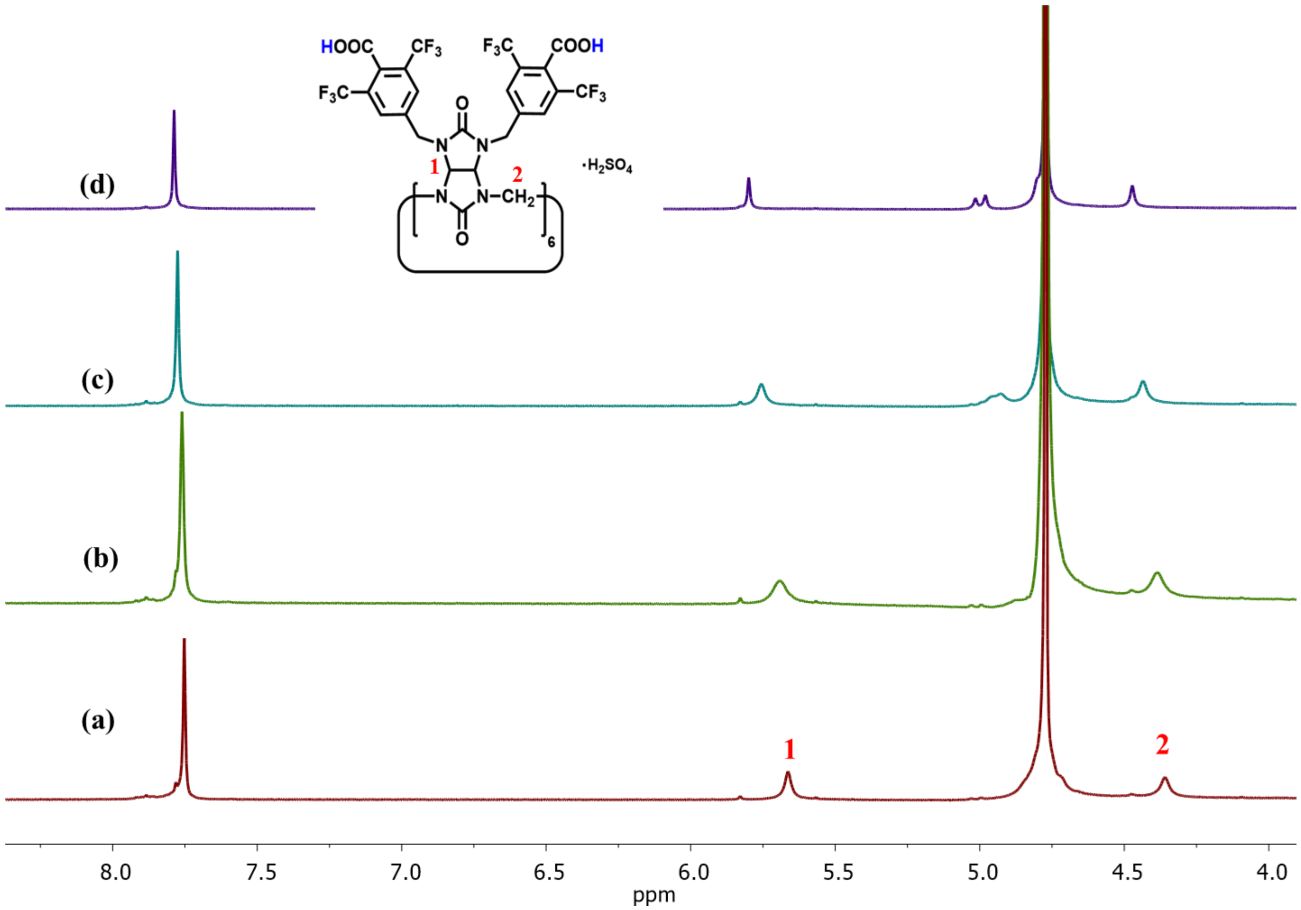

Figure S18: ^{1}H NMR (D_2O, 30 mM K_2DPO_4, 500 MHz) spectra of the $HSO_4^{-}\subset$BU2 complex (1 mM) (a) in the absence and in the presence of (b) 0.5 eq, (b) 1 eq, and (c) 1.2 eq of TMACl.

5. Dependence of Apparent Association Constants on Different Parameters

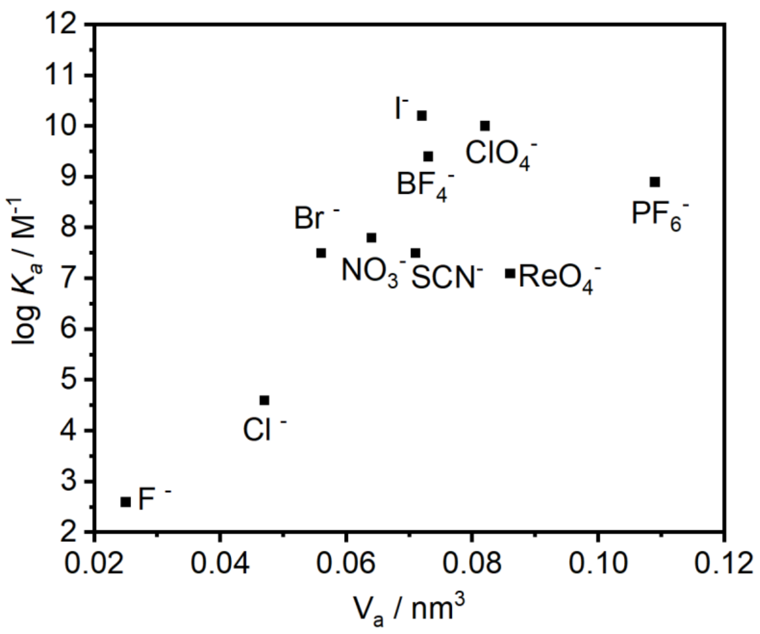

Figure S19: Dependence of apparent association constants on anions size.

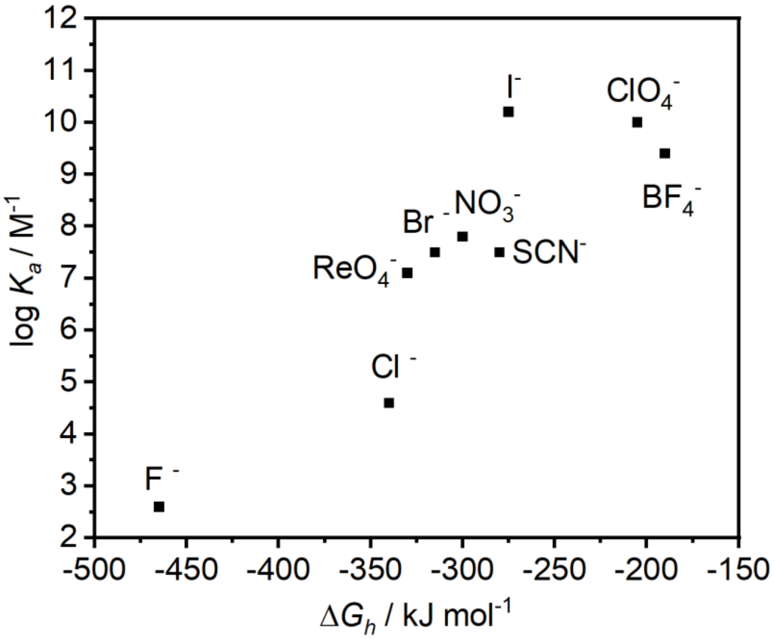

Figure S20: Dependence of apparent association constants on solvation of anions.

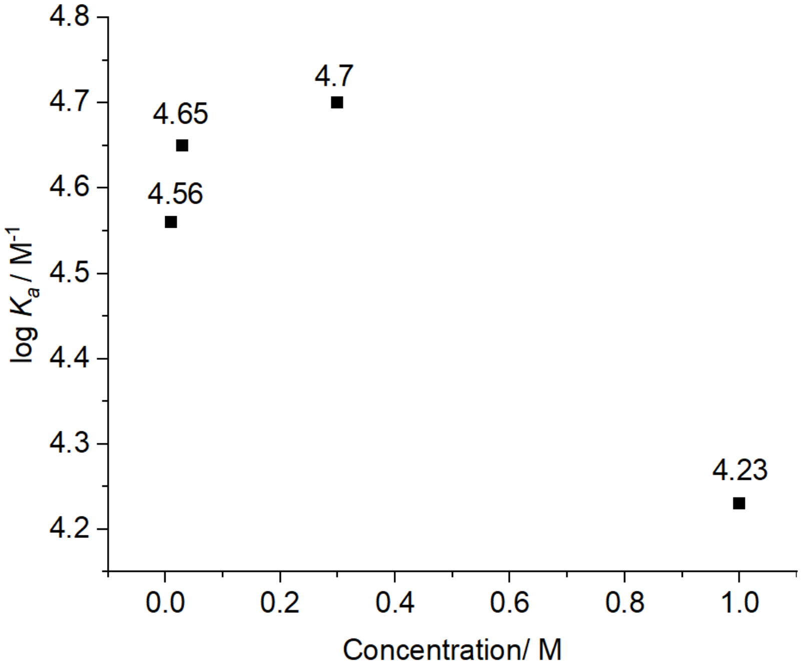

Figure S21: Dependence of apparent association constants of the TMACl⊂**BU2** complex on different concentration of buffer

6. ^{1}H, ^{19}F, and ^{13}C NMR Spectra of Synthesized Compounds

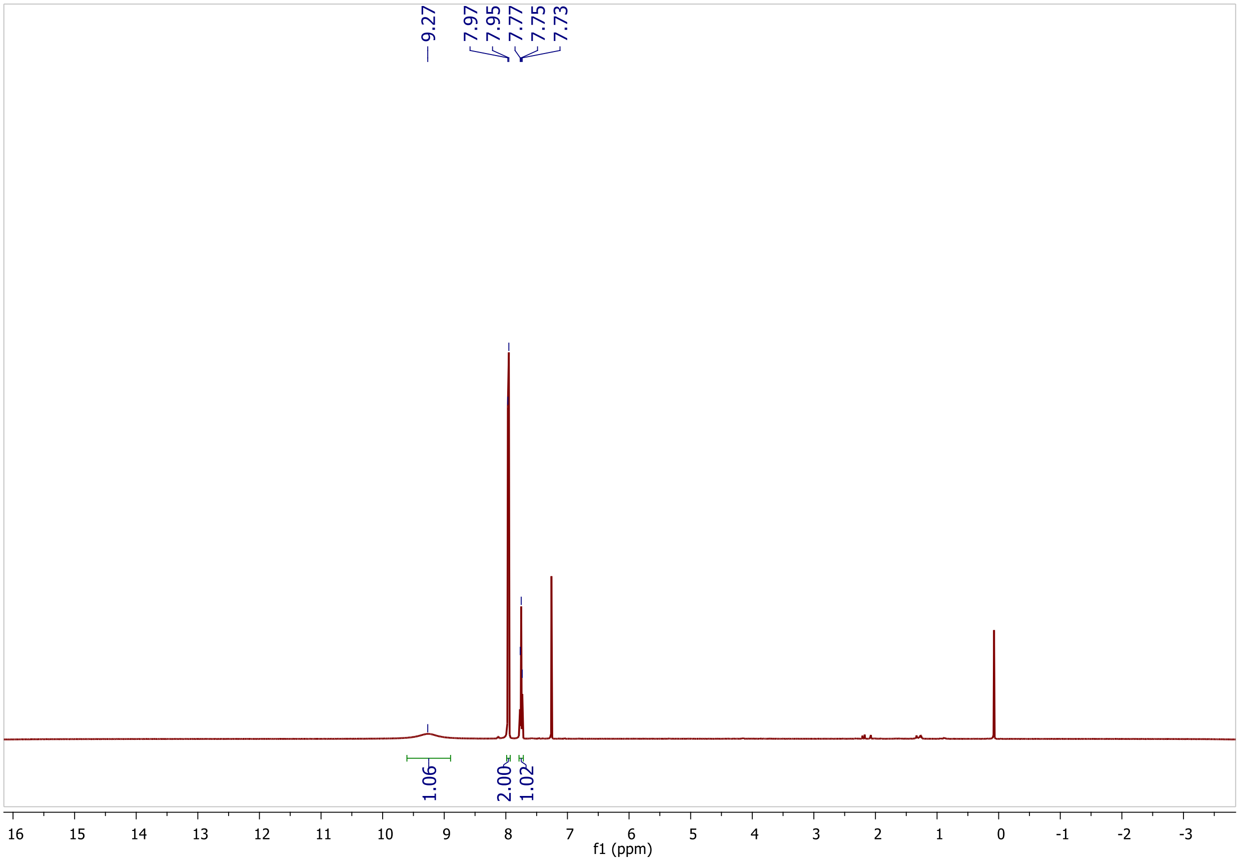

Figure S22: ^{1}H NMR spectrum of **2a** (500 MHz, $CDCl_3$).

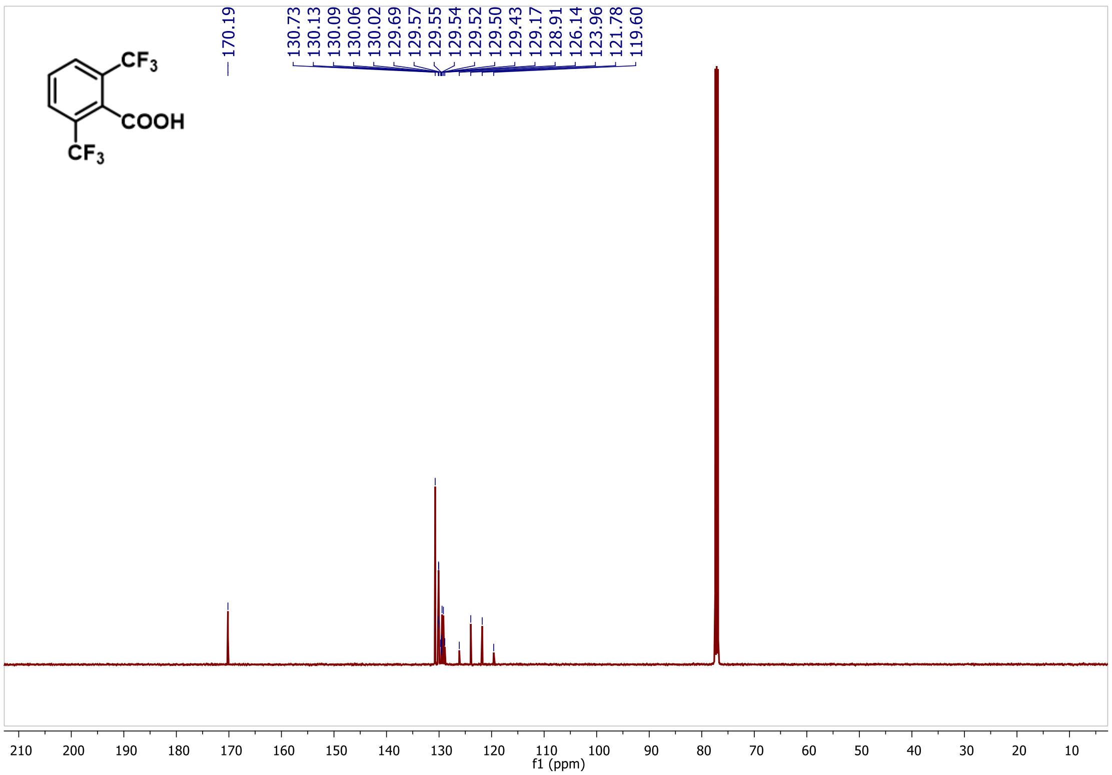

Figure S23: $^{13}C\{^{1}H\}$ NMR spectrum of **2a** (125 MHz, $CDCl_3$).

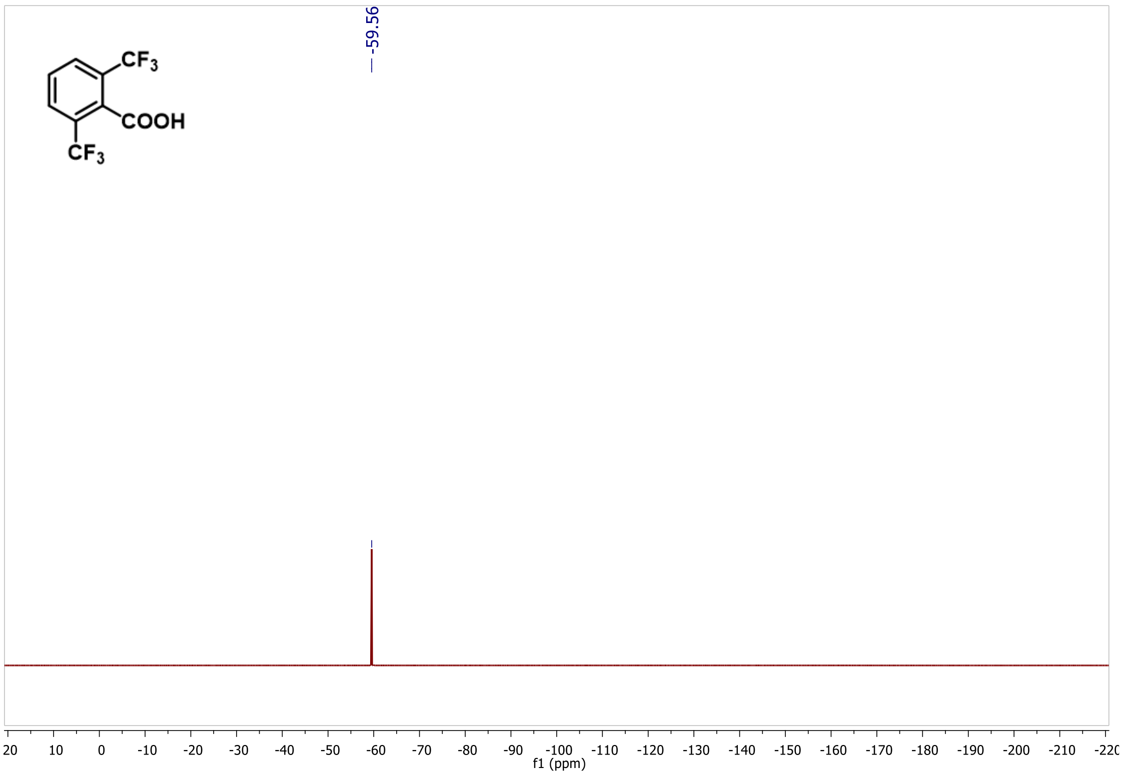

Figure S24: ^{19}F NMR spectrum of **2a** (471 MHz, $CDCl_3$).

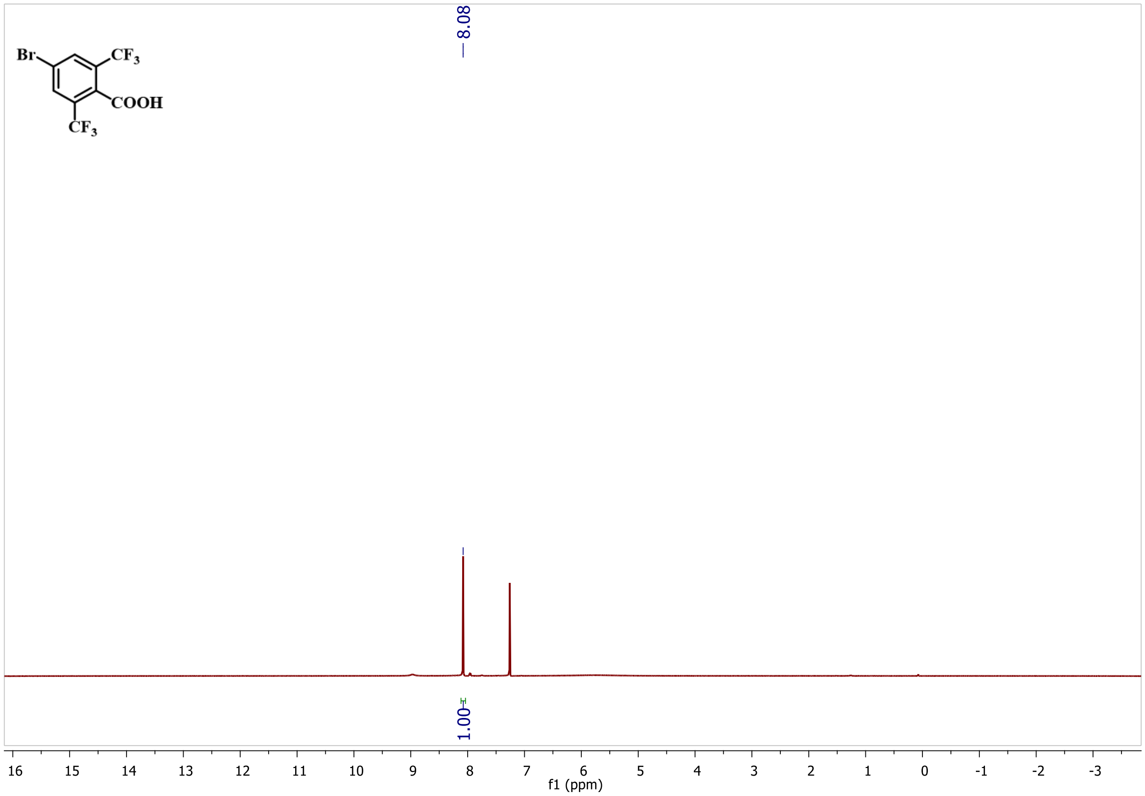

Figure S25: ^{1}H NMR spectrum of **3a** (500 MHz, $CDCl_3$).

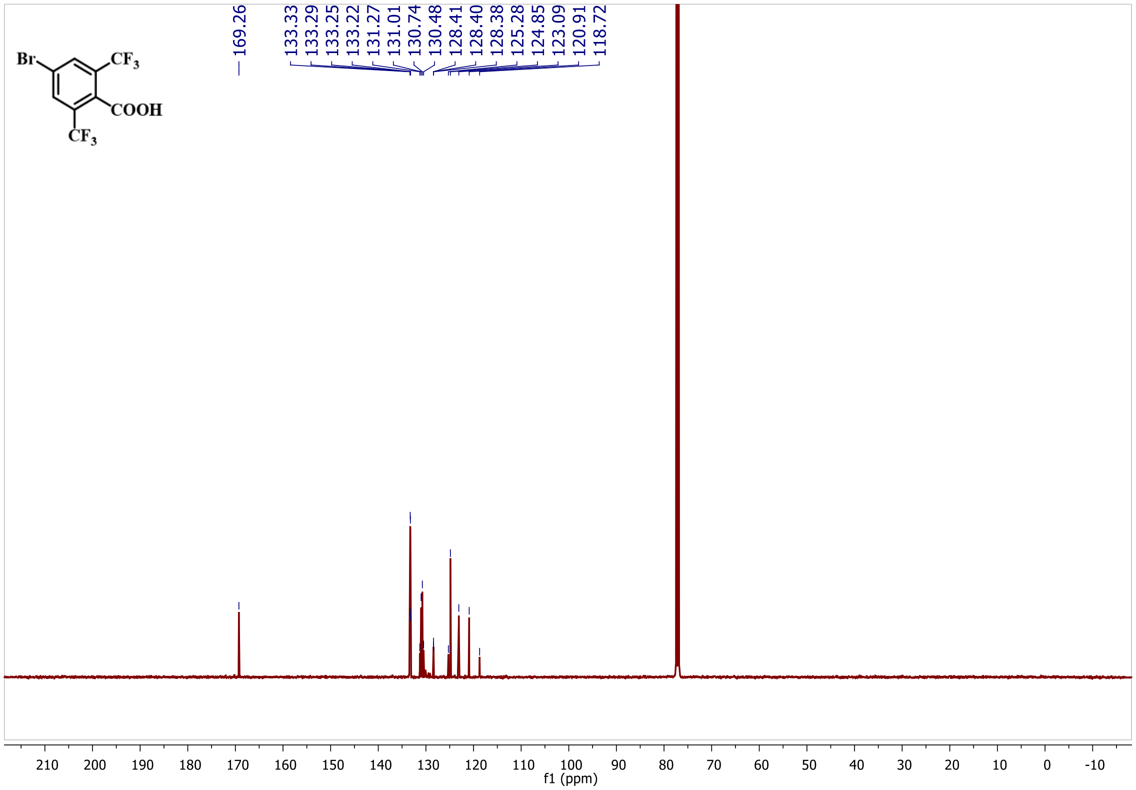

Figure S26: ^{13}C{^{1}H} NMR spectrum of **3a** (125 MHz, $CDCl_3$).

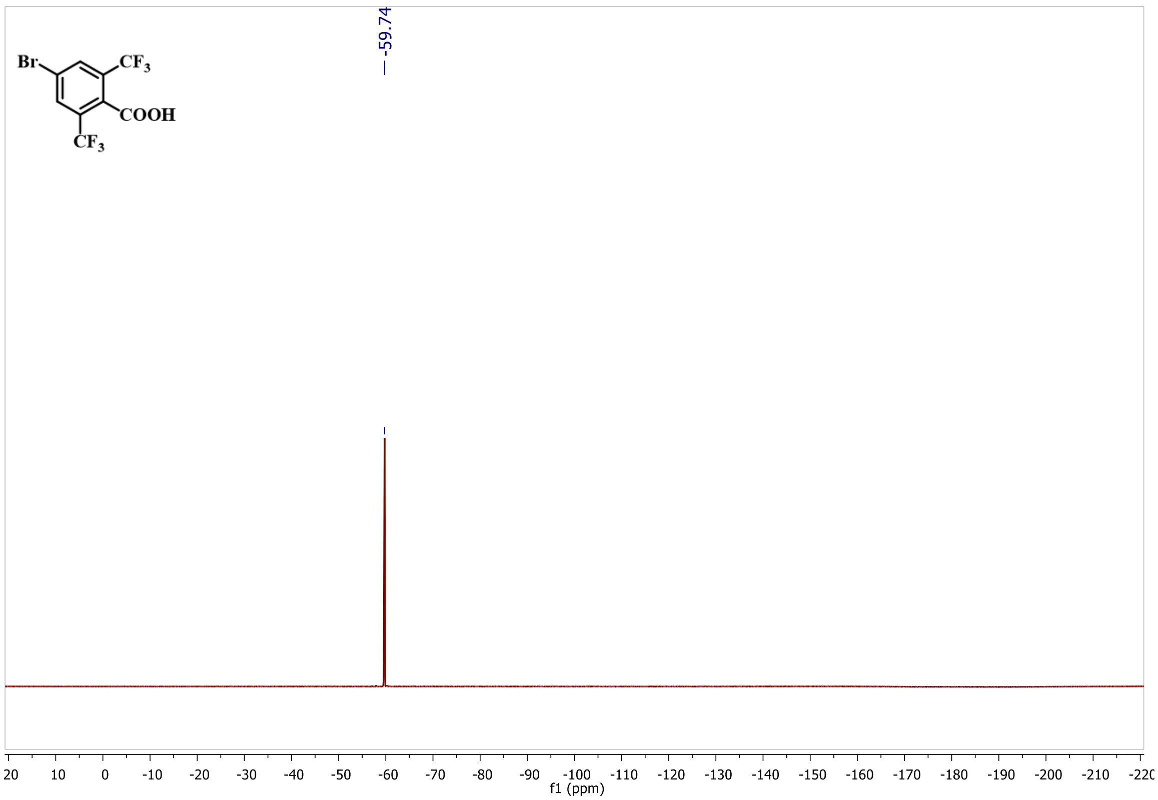

Figure S27: ^{19}F NMR spectrum of **3a** (471 MHz, $CDCl_3$).

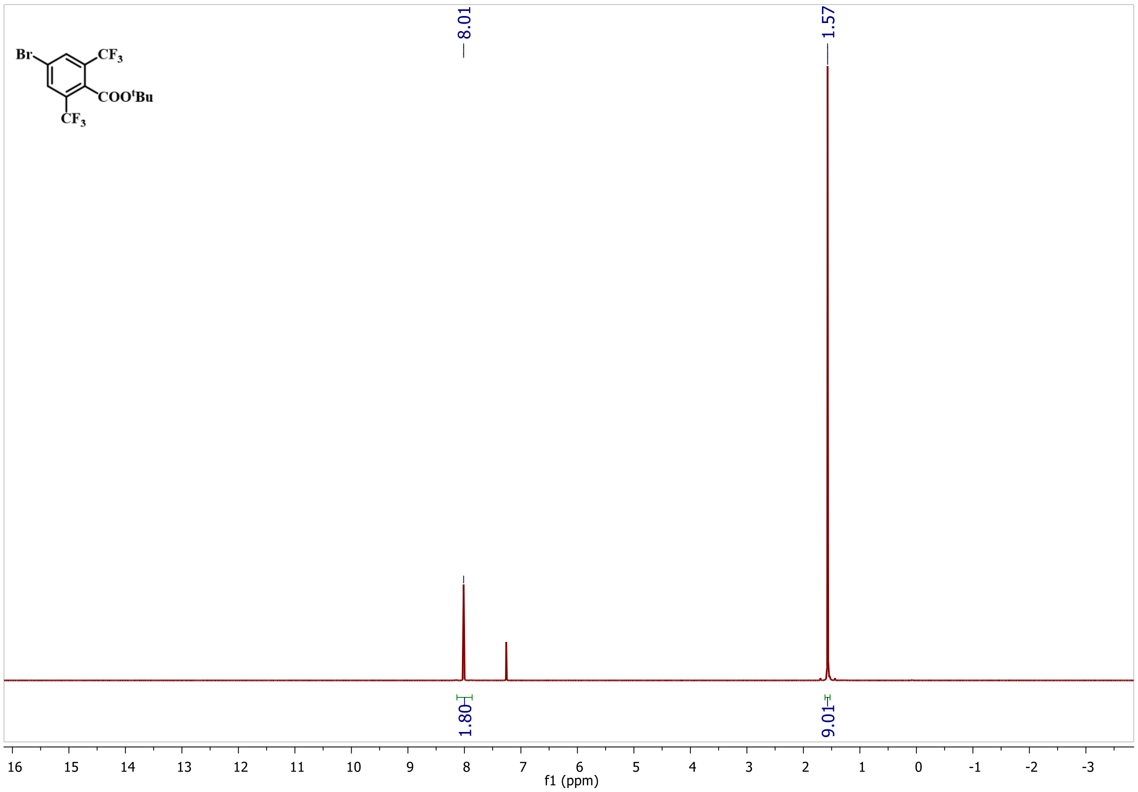

Figure S28: ^{1}H NMR spectrum of **4a** (500 MHz, $CDCl_3$).

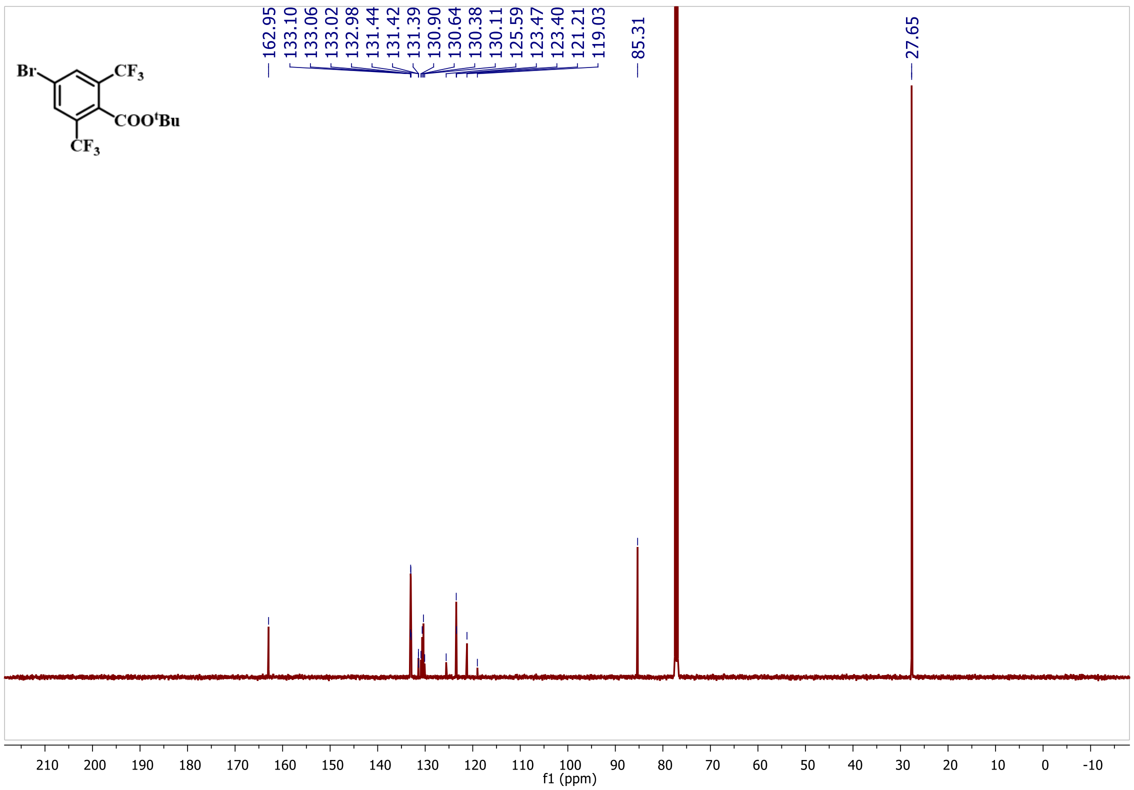

Figure S29: $^{13}C\{^{1}H\}$ NMR spectrum of **4a** (125 MHz, $CDCl_3$).

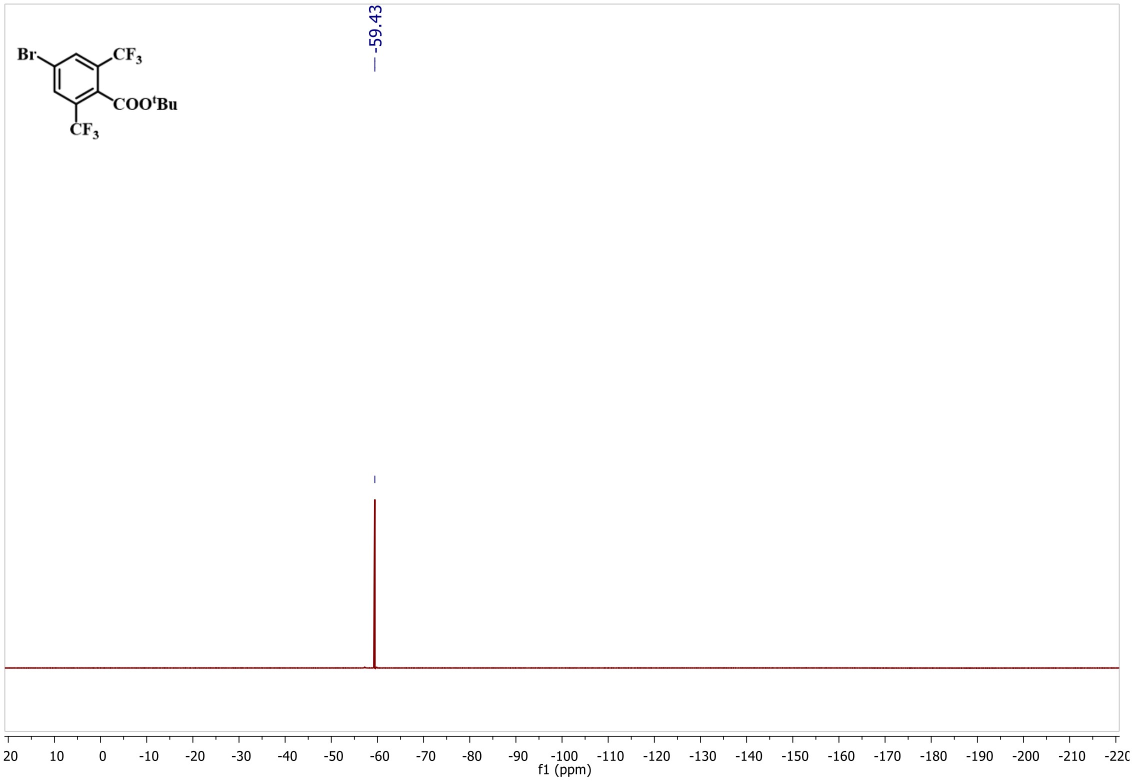

Figure S30: ^{19}F NMR spectrum of **4a** (471 MHz, $CDCl_3$).

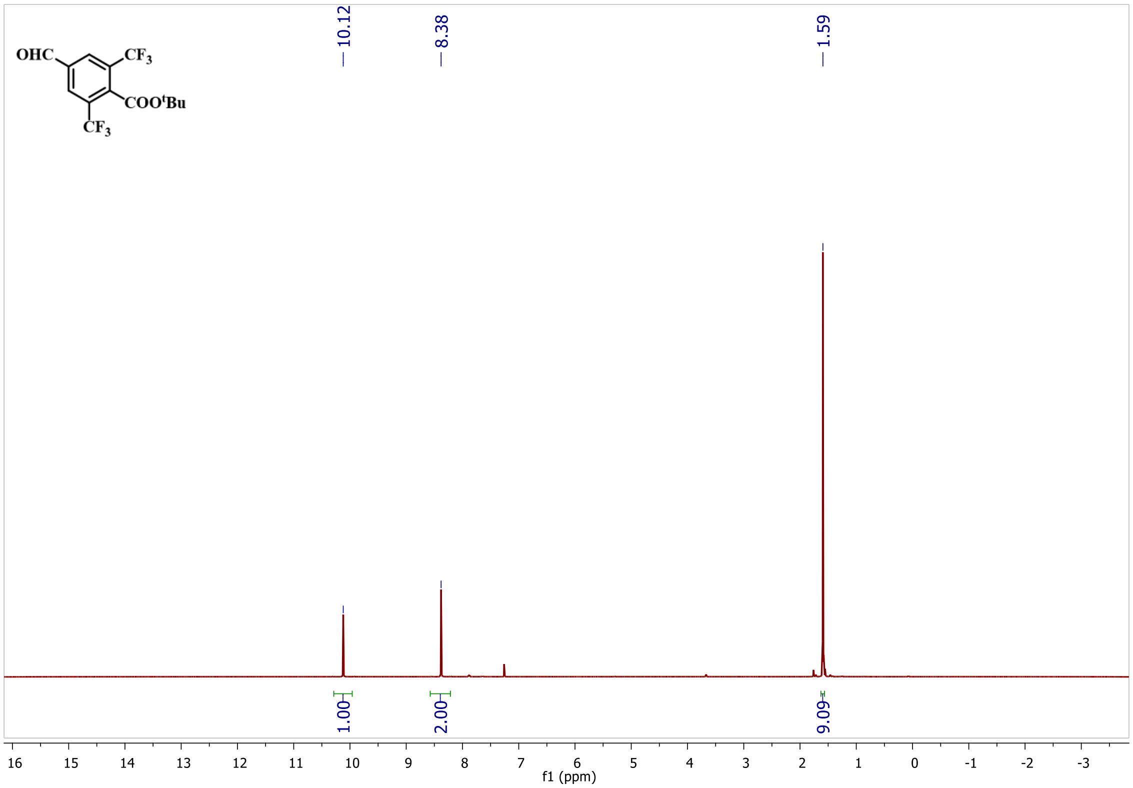

Figure S31: ^{1}H NMR spectrum of **5a** (500 MHz, $CDCl_3$).

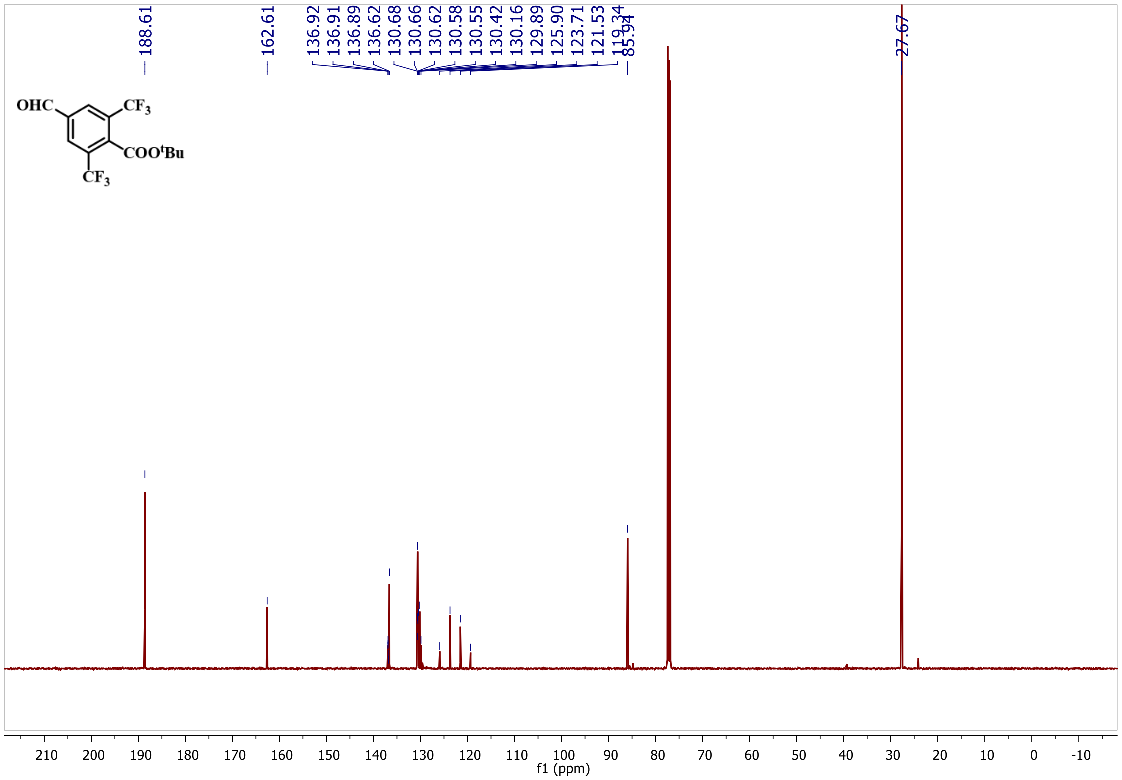

Figure S32: $^{13}C\{^{1}H\}$ NMR spectrum of **5a** (125 MHz, $CDCl_3$).

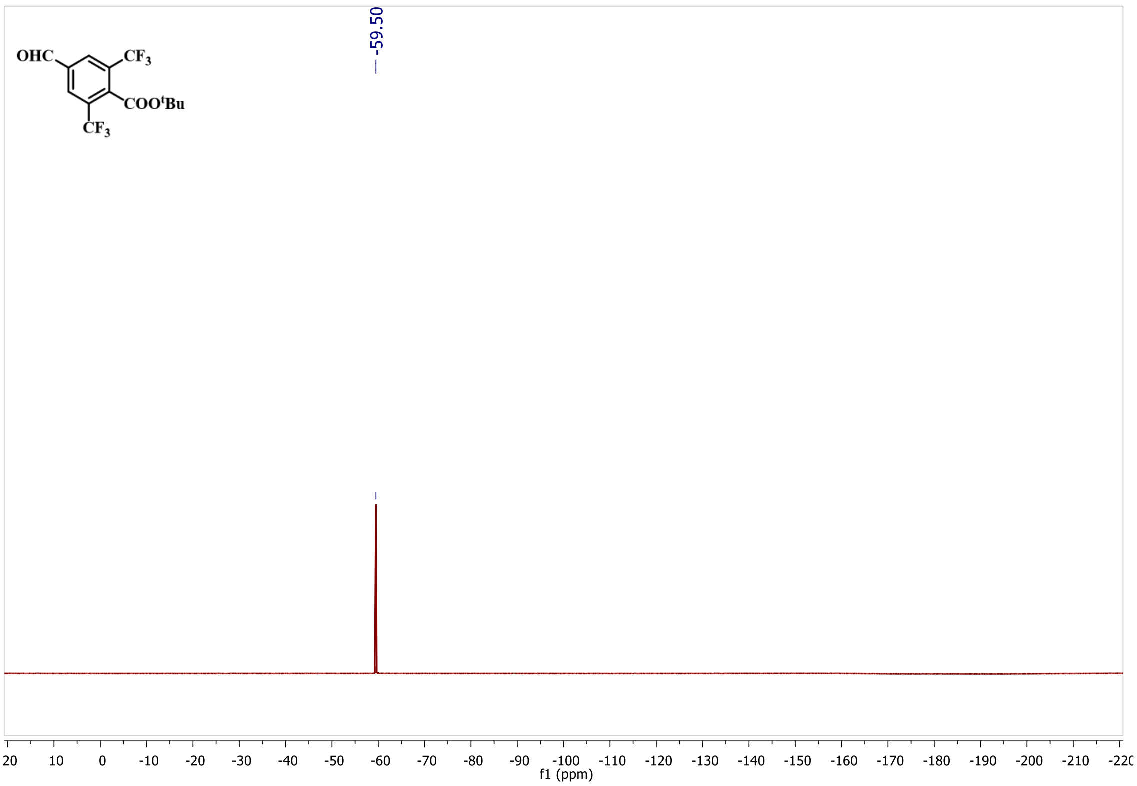

Figure S33: ^{19}F NMR spectrum of **5a** (471 MHz, $CDCl_3$).

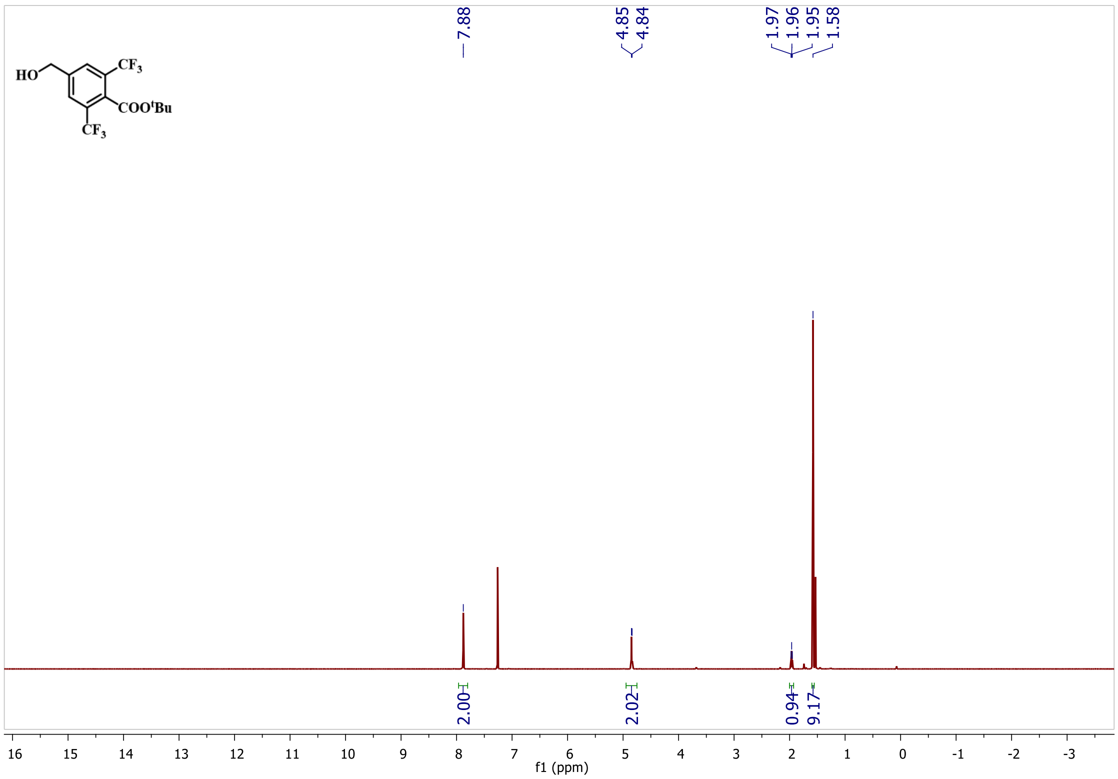

Figure S34: ^{1}H NMR spectrum of **6a** (500 MHz, $CDCl_3$).

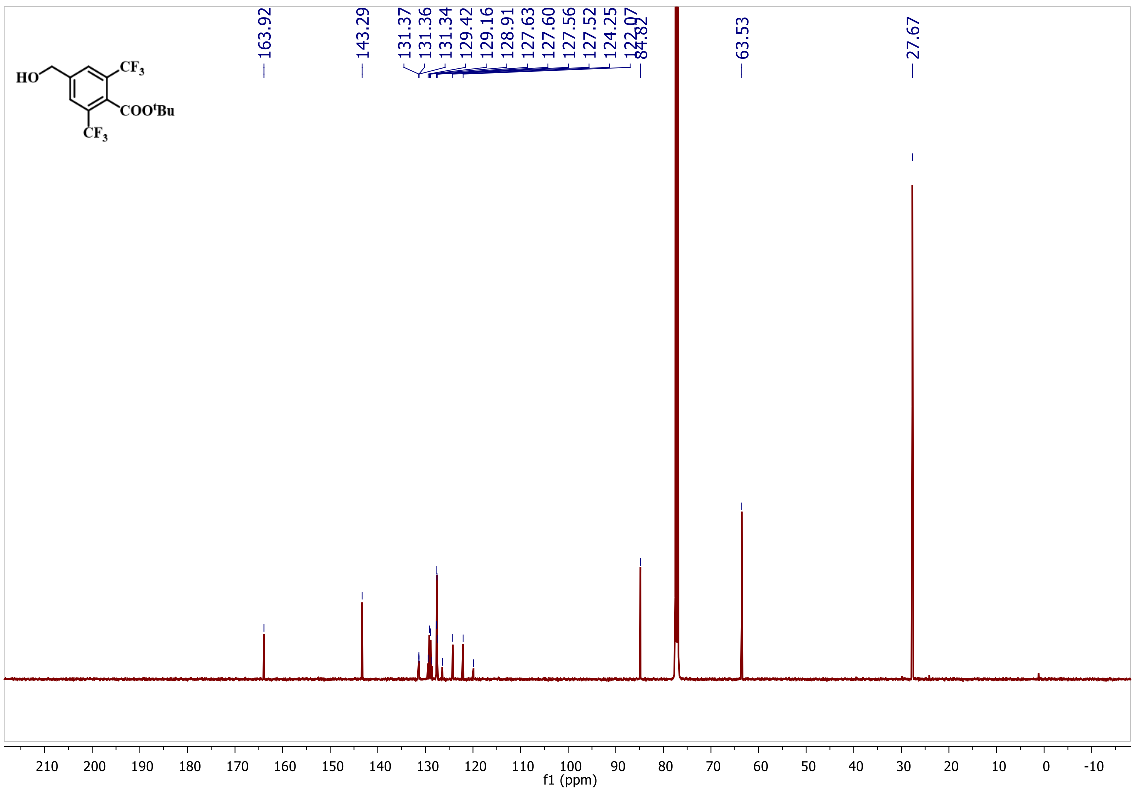

Figure S35: $^{13}C\{^{1}H\}$ NMR spectrum of **6a** (125 MHz, $CDCl_3$).

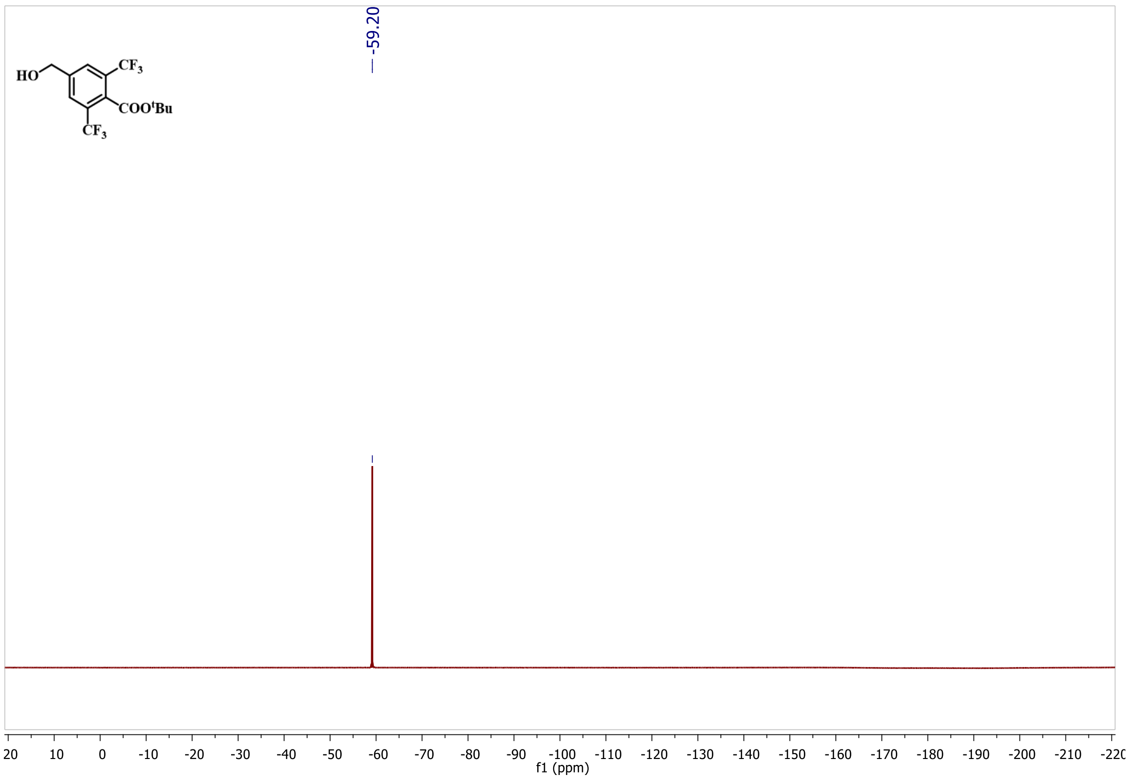

Figure S36: ^{19}F NMR spectrum of **6a** (471 MHz, $CDCl_3$).

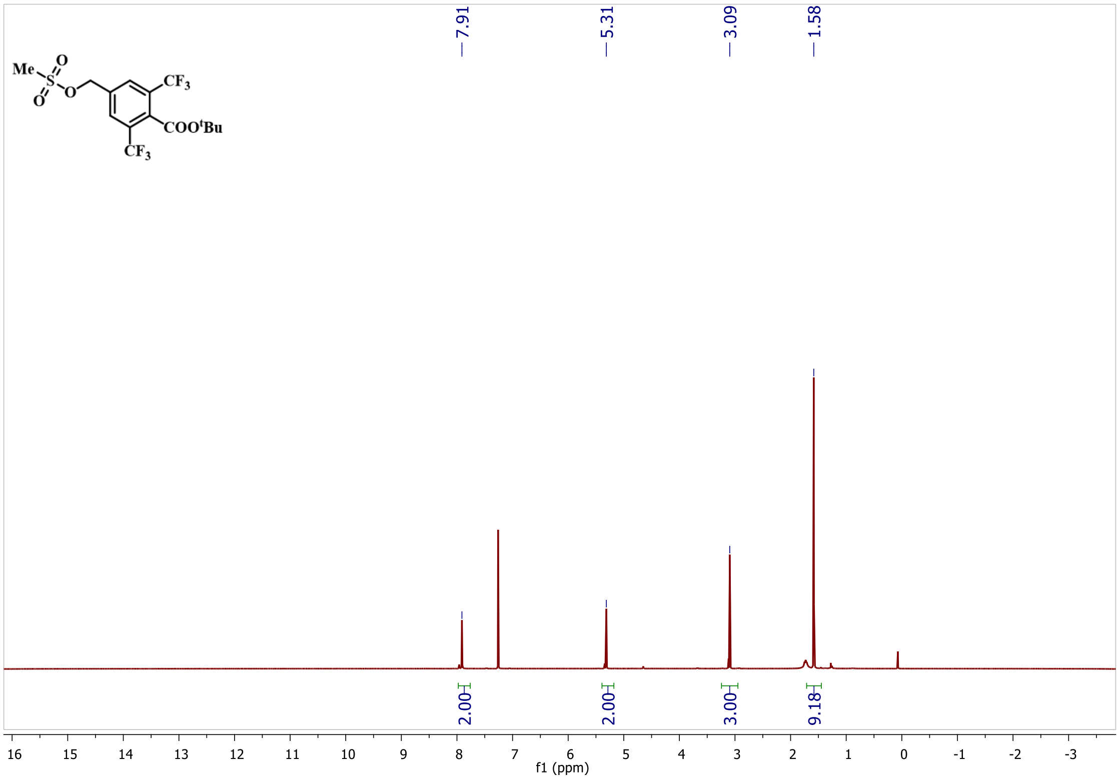

Figure S37: ^{1}H NMR spectrum of **1** (500 MHz, $CDCl_3$).

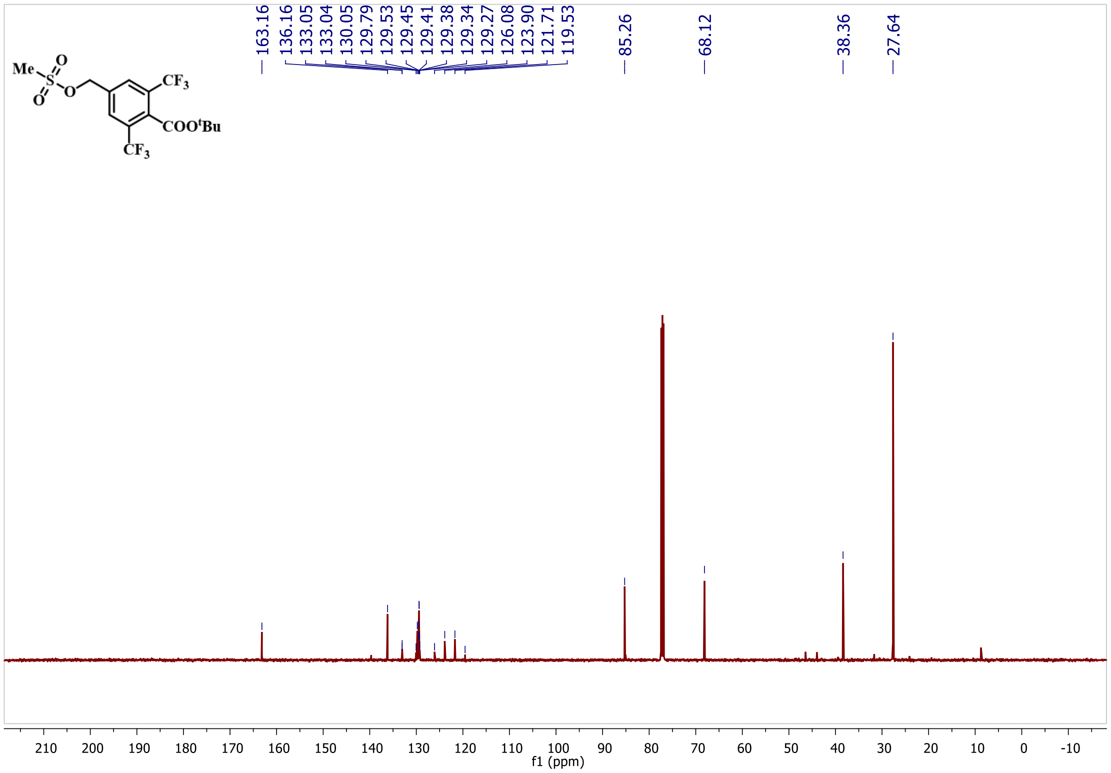

Figure S38: $^{13}C\{^{1}H\}$ NMR spectrum of **1** (125 MHz, $CDCl_3$).

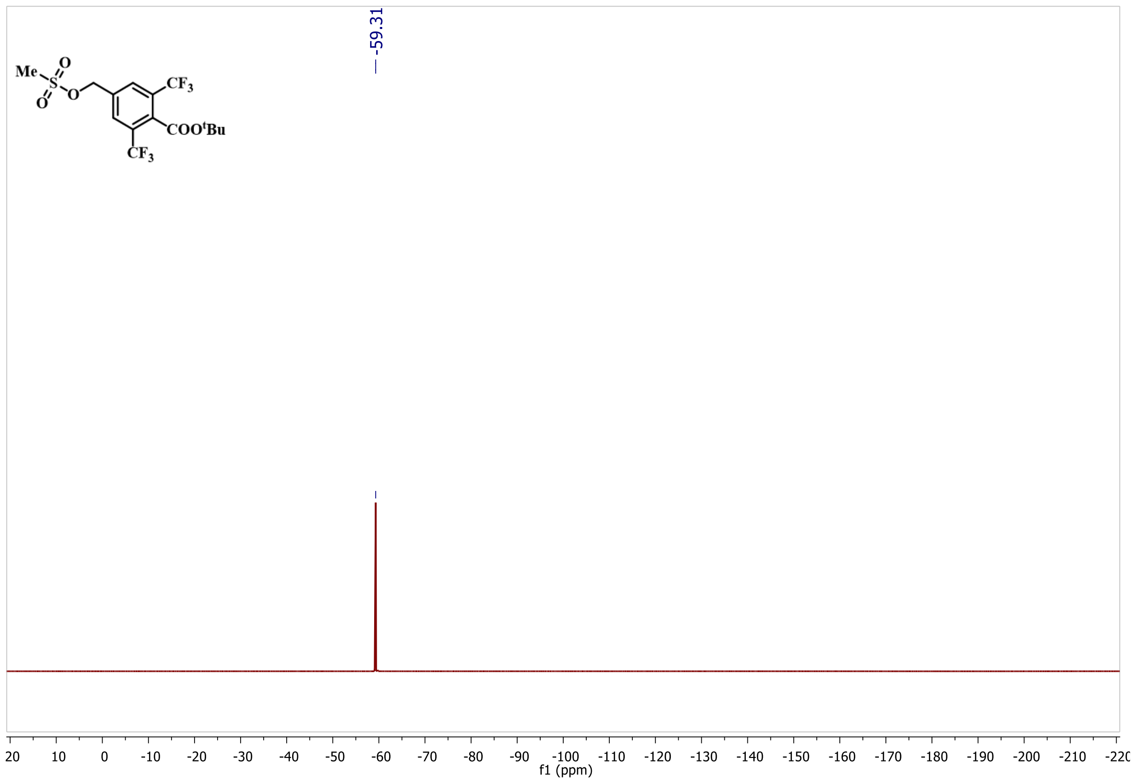

Figure S39: ^{19}F NMR spectrum of **1** (471 MHz, $CDCl_3$).

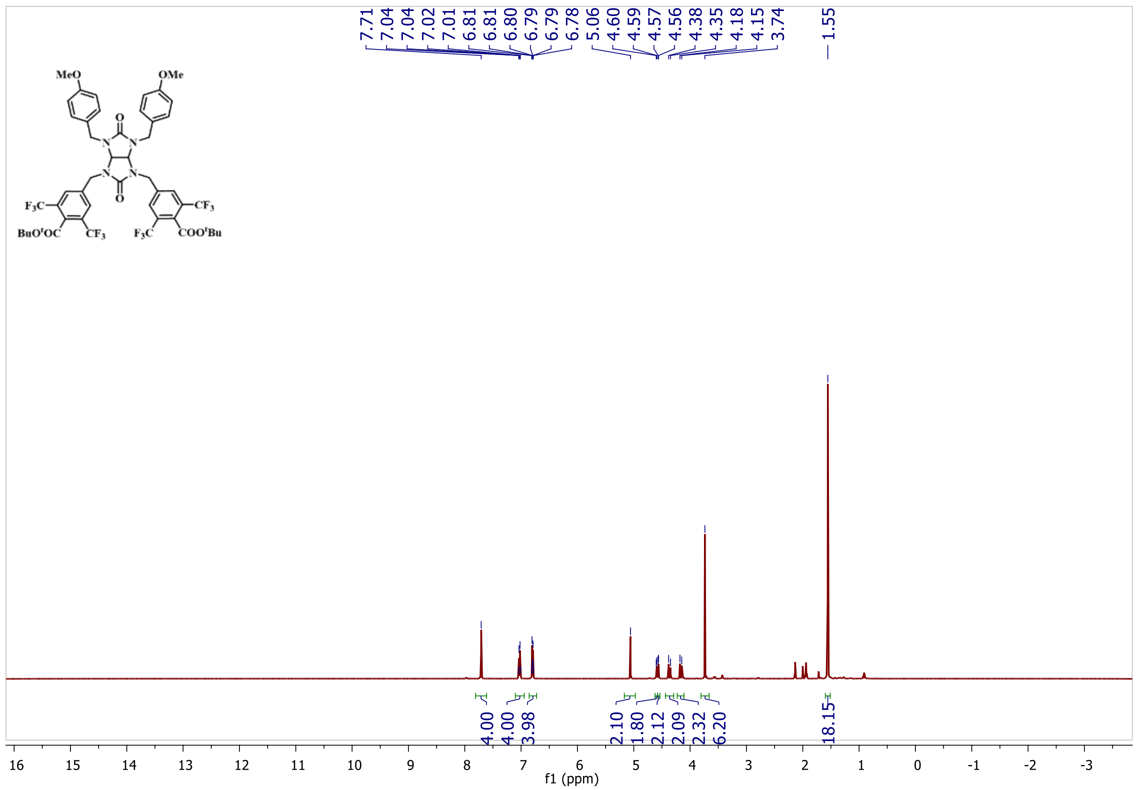

Figure S40: ^{1}H NMR spectrum of **3** (500 MHz, CD_3CN).

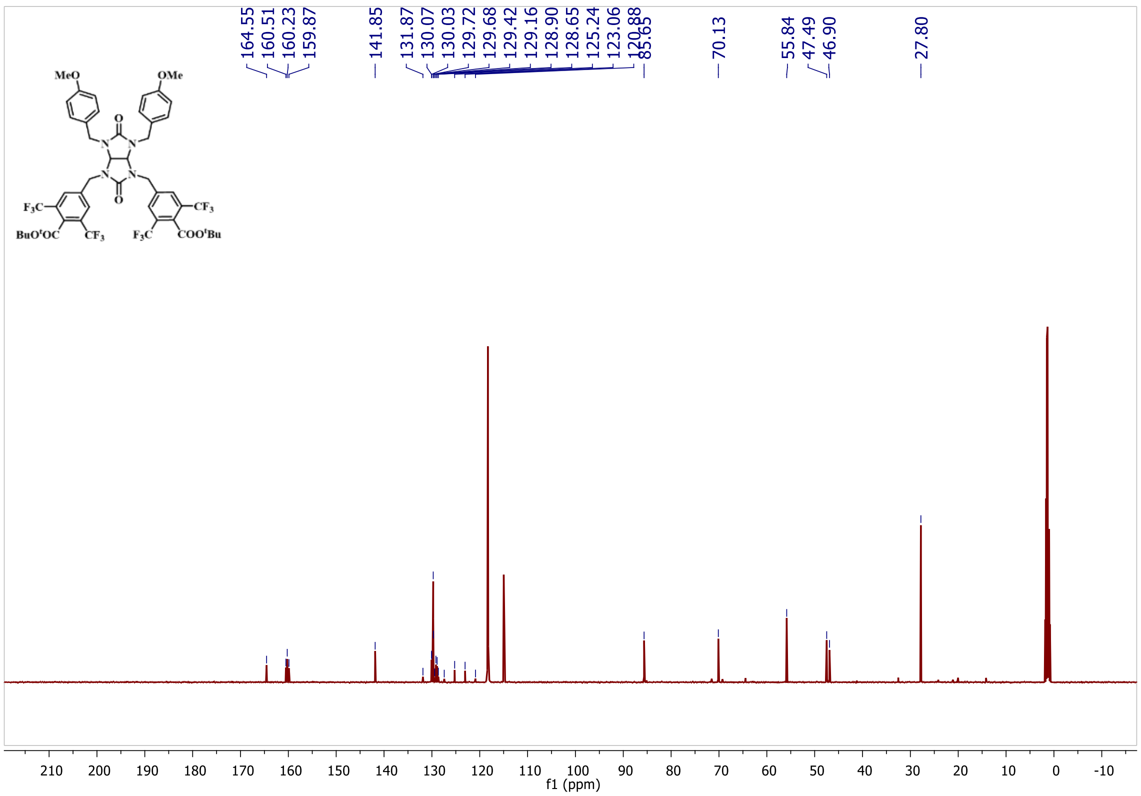

Figure S41: $^{13}C\{^{1}H\}$ NMR spectrum of **3** (125 MHz, CD_3CN).

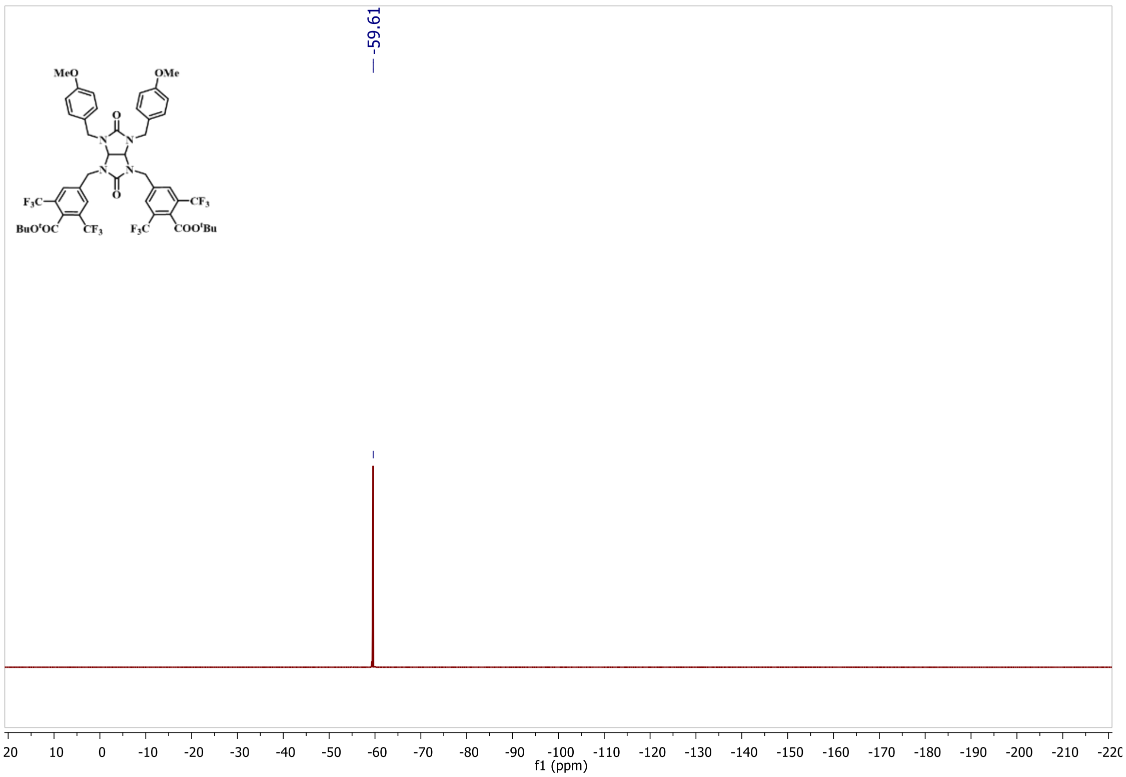

Figure S42: ^{19}F NMR spectrum of **3** (471 MHz, CD_3CN).

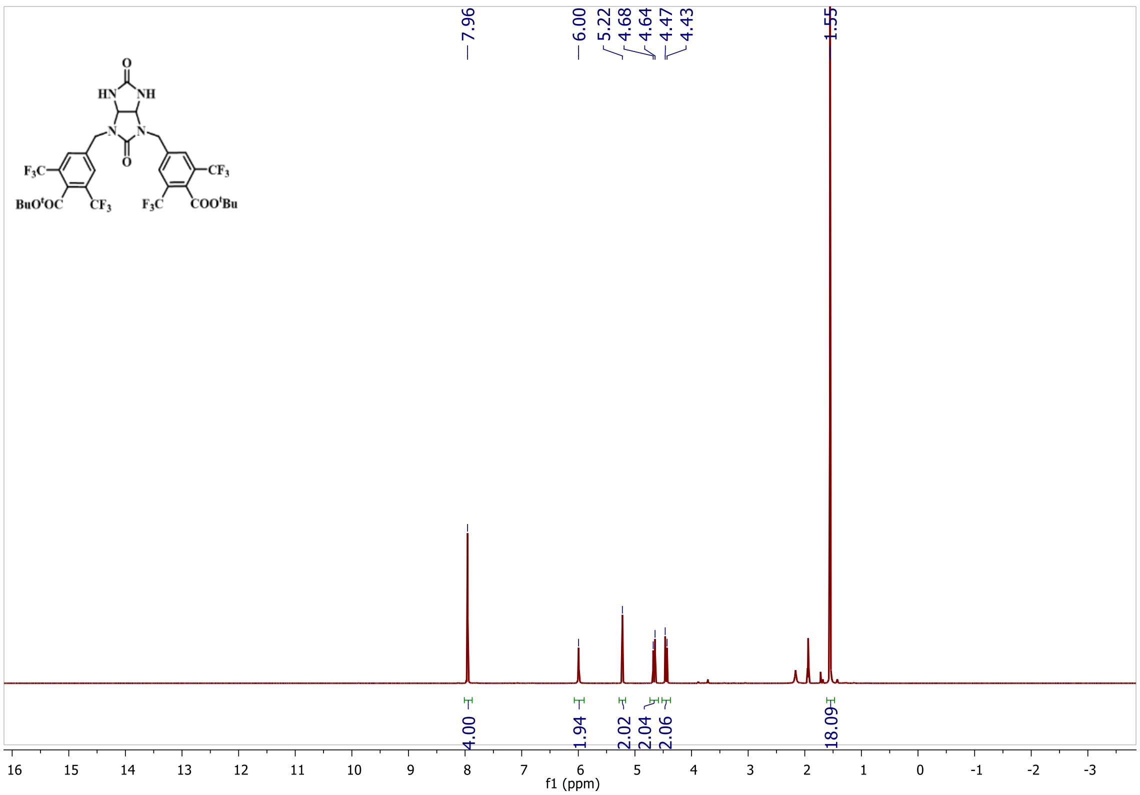

Figure S43: ^{1}H NMR spectrum of **4** (500 MHz, CD_3CN).

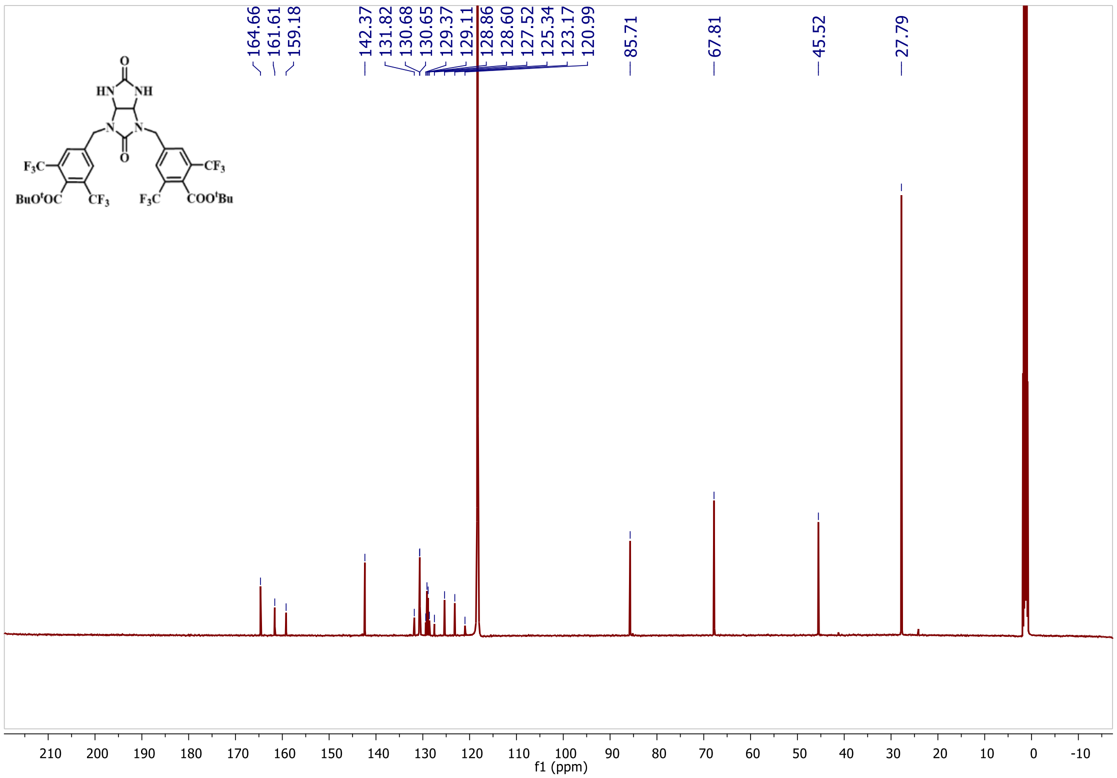

Figure S44: $^{13}C\{^{1}H\}$ NMR spectrum of **4** (125 MHz, CD_3CN).

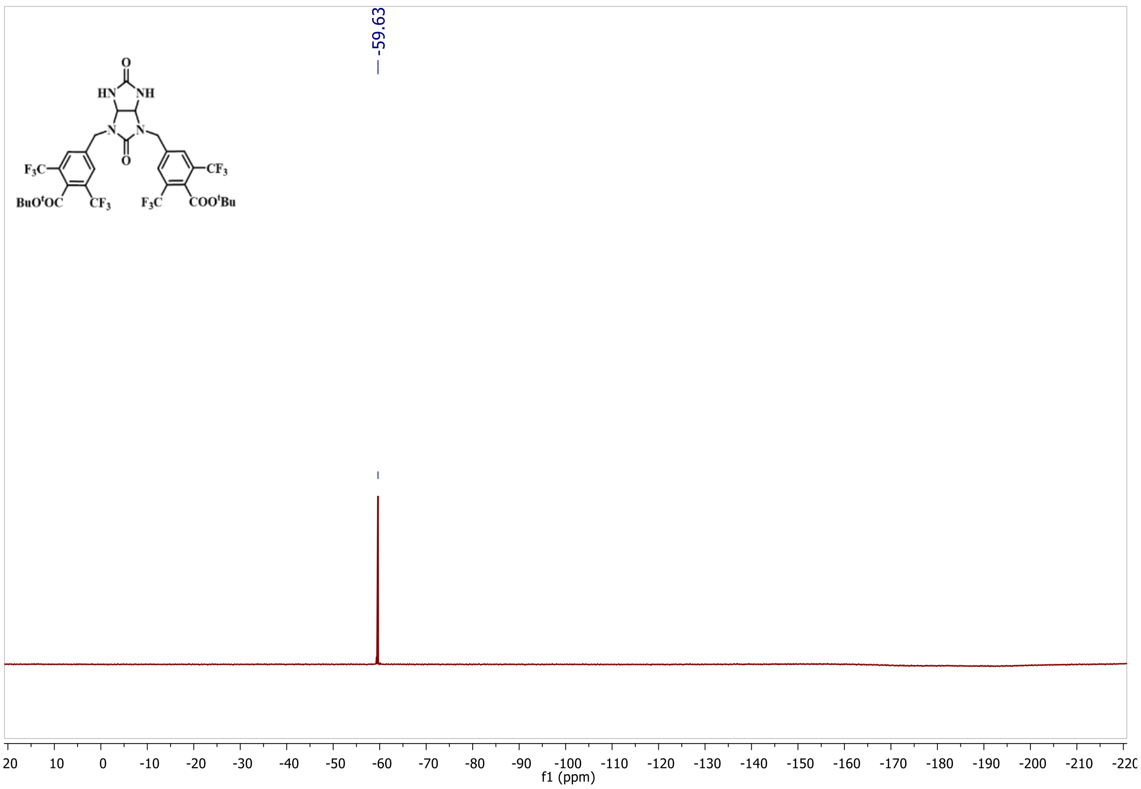

Figure S45: ^{19}F NMR spectrum of **4** (471 MHz, CD_3CN).

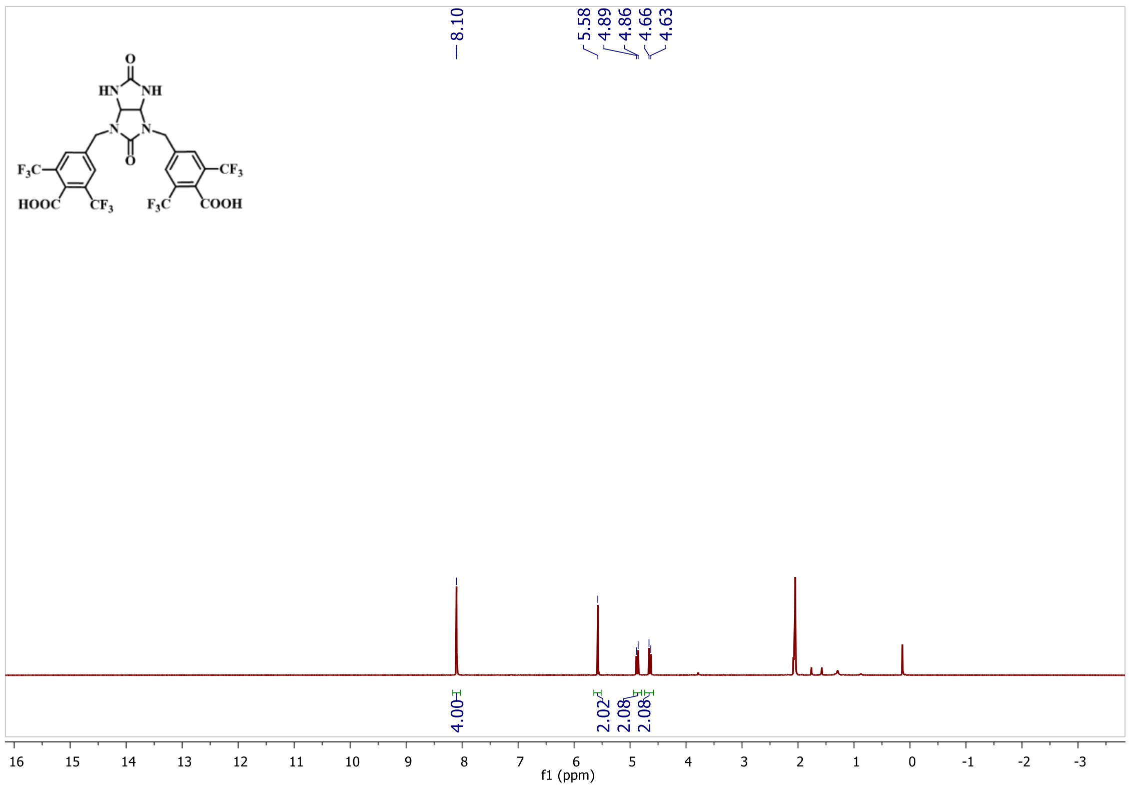

Figure S46: ^{1}H NMR spectrum of **5** (500 MHz, $(CD_3)_2CO$).

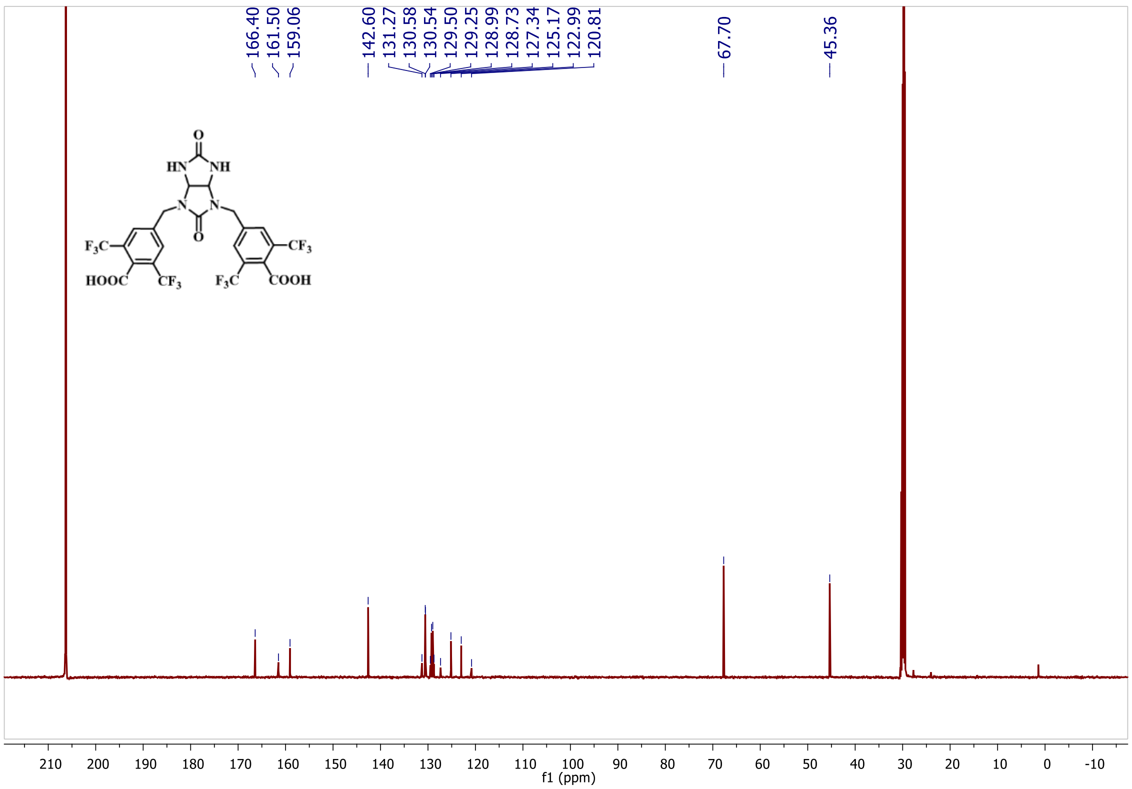

Figure S47: $^{13}C\{^{1}H\}$ NMR spectrum of **5** (125 MHz, $(CD_3)_2CO$).

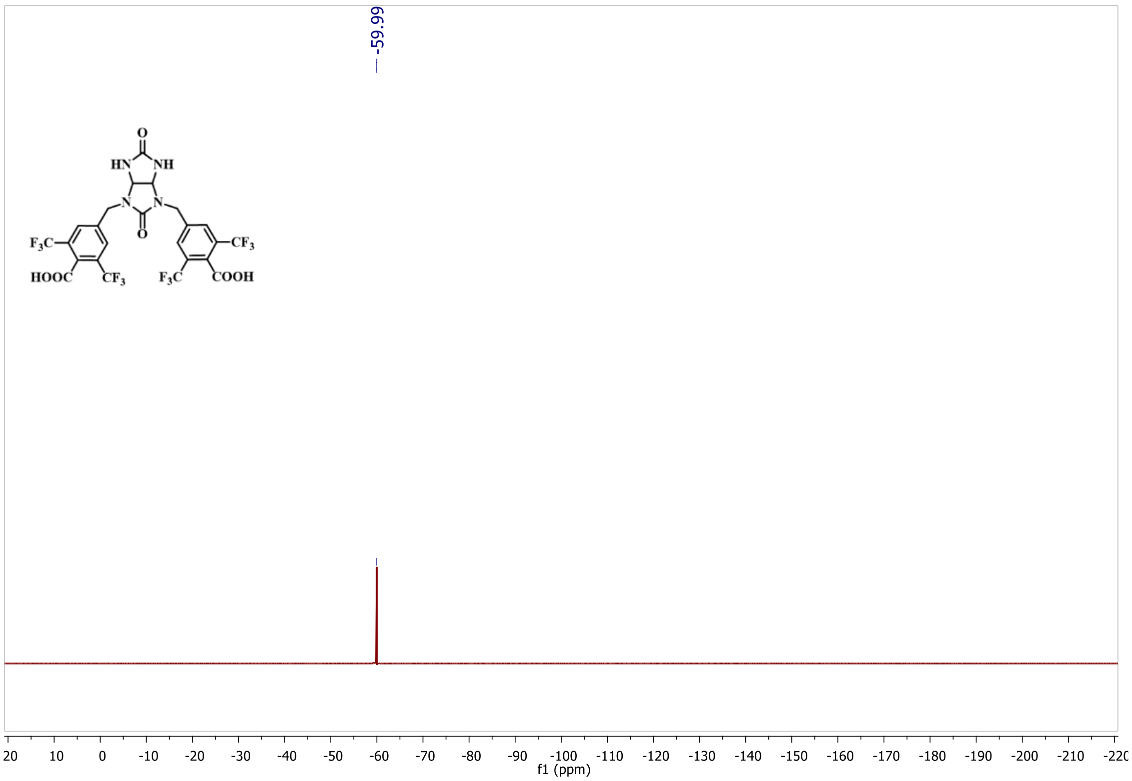

Figure S48: ^{19}F NMR spectrum of **5** (471 MHz, $(CD_3)_2CO$).

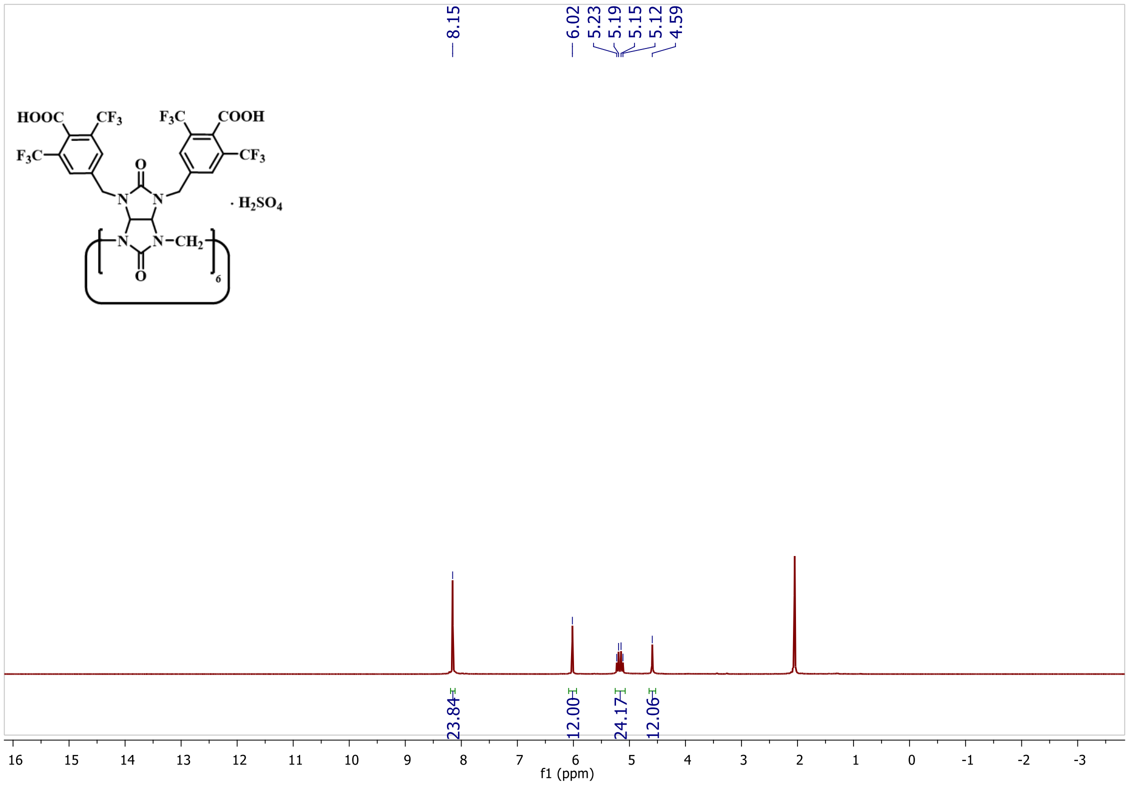

Figure S49: ^{1}H NMR spectrum of $HSO_4^- \subset$BU2 (500 MHz, $(CD_3)_2CO$).

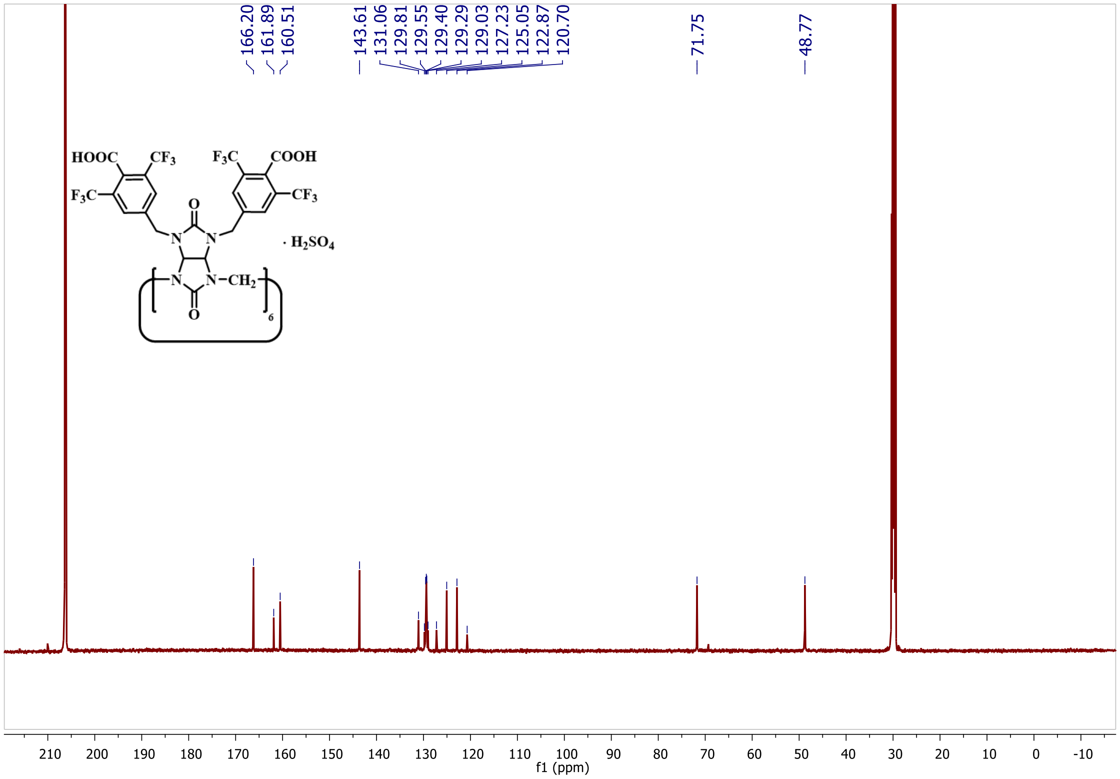

Figure S50: $^{13}C\{^{1}H\}$ NMR spectrum of $HSO_4^- \subset$BU2 (125 MHz, $(CD_3)_2CO$).

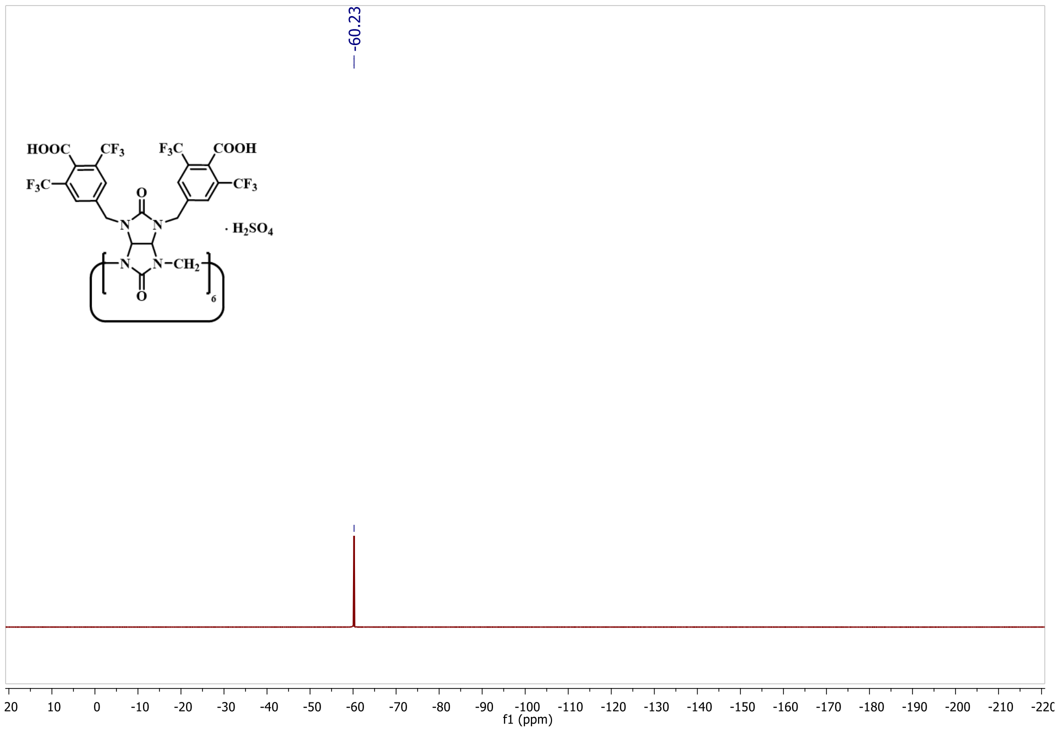

Figure S51: ^{19}F NMR spectrum of HSO_4^-⊂BU2 (471 MHz, $(CD_3)_2CO$).

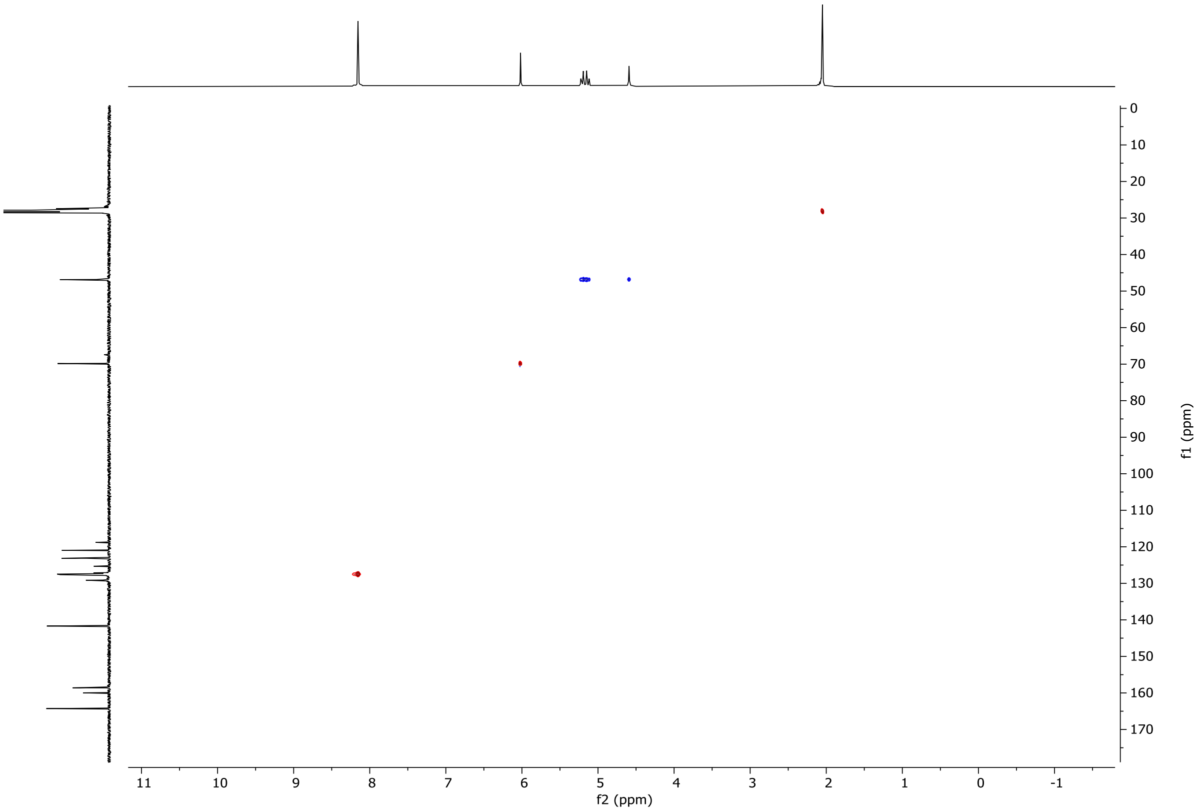

Figure S52: ^{1}H-^{13}C HSQC NMR spectrum of HSO_4^-⊂BU2 (500 MHz, 125 MHz, $(CD_3)_2CO$).

References:

(1) Havel, V.; Sadilová, T.; Šindelář, V. Unsubstituted Bambusurils: Post-Macrocyclization Modification of Versatile Intermediates. *ACS Omega* **2018**, *3* (4), 4657–4663. https://doi.org/10.1021/acsomega.8b00497.

(2) De Simone, N. A.; Chvojka, M.; Lapešová, J.; Martínez-Crespo, L.; Slávik, P.; Sokolov, J.; Butler, S. J.; Valkenier, H.; Šindelář, V. Monofunctionalized Fluorinated Bambusurils and Their Conjugates for Anion Transport and Extraction. *J. Org. Chem.* **2022**, *87* (15), 9829–9838. https://doi.org/10.1021/acs.joc.2c00870.
